# Simultaneous effect of different chromatographic conditions on the chromatographic retention of pentapeptide derivatives (HGRFG and NPNPT)

**DOI:** 10.3389/fchem.2023.1171824

**Published:** 2023-04-18

**Authors:** Huan Peng, Xiangrong Yang, Huanle Fang, Zhongqi Zhang, Jinli Zhao, Te Zhao, Jianli Liu, Yan Li

**Affiliations:** ^1^ Center for Brain Science, The First Affiliated Hospital of Xi’ an Jiaotong University, Xi’an, Shaanxi, China; ^2^ College of Life Science, Northwest University, Xi’an, Shaanxi, China; ^3^ Kangya of Ningxia Pharmaceutical Co., Ltd., Yinchuan, China; ^4^ Medical College, Peihua University, Xi’an, Shaanxi, China; ^5^ Department of Polypeptide Engineering, Active Protein and Polypeptide Engineering Center of Xi’an Hui Kang, Xi’an, Shaanxi, China; ^6^ College of Electronic Engineering, Xidian University, Xi’an, Shaanxi, China

**Keywords:** chromatographic retention, pentapeptides, six-parameter model, retention factor, prediction capacity

## Abstract

**Introduction:** Oligopeptides exhibit great prospects for clinical application and its separation is of great importance in new drug development.

**Methods:** To accurately predict the retention of pentapeptides with analogous structures in chromatography, the retention times of 57 pentapeptide derivatives in seven buffers at three temperatures and four mobile phase compositions were measured via reversed-phase high-performance liquid chromatography. The parameters ( $k_{HA}$ , $k_{A}$ , and pKa ) of the acid–base equilibrium were obtained by fitting the data corresponding to a sigmoidal function. We then studied the dependence of these parameters on the temperature (T), organic modifier composition (φ, methanol volume fraction), and polarity ( $P_{m}^{N}$ parameter). Finally, we proposed two six-parameter models with (1) pH and T and (2) pH and φ or $P_{m}^{N}$ as the independent variables. These models were validated for their prediction capacities by linearly fitting the predicted retention factor k-value and the experimental k-value.

**Results:** The results showed that $\log k_{HA}$ and $\log k_{A}$ exhibited linear relationships with 1/T , φ or $P_{m}^{N}$ for all pentapeptides, especially for the acid pentapeptides. In the model of pH and T, the correlation coefficient (R^2^) of the acid pentapeptides was 0.8603, suggesting a certain prediction capability of chromatographic retention. Moreover, in the model of pH and φ or $P_{m}^{N}$ , the R^2^ values of the acid and neutral pentapeptides were greater than 0.93, and the average root mean squared error was approximately 0.3, indicating that the k-values could be effectively predicted.

**Discussion:** In summary, the two six-parameter models were appropriate to characterize the chromatographic retention of amphoteric compounds, especially the acid or neutral pentapeptides, and could predict the chromatographic retention of pentapeptide compounds.

## Graphical Abstract

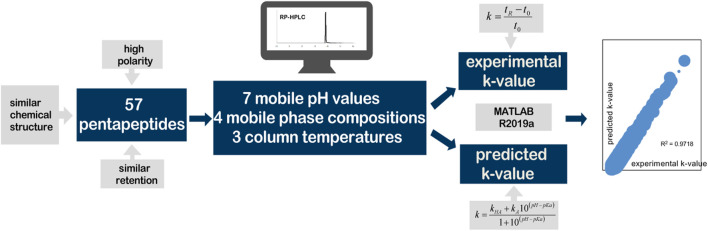

## 1 Introduction

Active peptides are common small-molecule compounds in nature and generally possess invaluable medicinal value (Abdelhedi and Nasri, 2019; Suo et al., 2022). Peptides have a wide range of bioactivities and can be divided into two categories according to different sources: (1) endogenous peptides from precursor proteins and secreted cells and (2) exogenous peptides from enzymatic hydrolysis or synthesis (Wang et al., 2022). The oligopeptides produced by the enzymatic hydrolysis of animal proteins have been reported to exhibit outstanding hypotensive effects by inhibiting the angiotensin-I converting enzyme (Abdelhedi et al., 2017; Qiao et al., 2022). Moreover, the oligopeptides extracted from tea and brewer’s spent grain had excellent hypolipidemic activities (Ferreira et al., 2022; Ye et al., 2023), and the oligopeptides isolated from Siberian sturgeon cartilage could treat chronic diseases caused by oxidative stress (Sheng et al., 2022). More importantly, oligopeptides exhibit great prospects for clinical application due to their high degree of affinity and specificity and easy absorption (Zhang et al., 2020; Sitkov et al., 2021).

Oligopeptide separations by reversed-phase high-performance liquid chromatography (RP-HPLC) are extremely common (Ofosu et al., 2021; Samtiya et al., 2021; Waili et al., 2021), and the chromatographic retention of oligopeptides in RP-HPLC is driven by hydrophobic interactions (Sousa et al., 2021). Combining the molecular structure of compounds with the parameters describing the properties of chromatographic mobile and stationary phases, functional relationships could be obtained (Nie et al., 2022). These relationships can be used to analyze and predict the chromatographic behavior of other compounds (Janicka et al., 2020; Nie et al., 2022) and evaluate the pharmacokinetics and biochemical properties of drugs, such as absorption, distribution, metabolism, and excretion (ADME) *in vivo* (Langyan et al., 2021). It can also preliminarily determine the solubility, lipophilicity, bioaccumulation, and toxicity of compounds *in vivo*, which is of great significance in the field of new drug molecule development, especially in the analysis of the chemical properties of peptides *in vivo*.

The acid dissociation constant (pKa) is an elementary parameter in the analysis of drugs and strongly affects their pharmacokinetics and biochemical properties by characterizing the degree of ionization of drug molecules in solution at different pH values (Besleaga et al., 2021). pKa determines the existing form of compounds in the medium and their solubility, lipophilicity, permeability, bioaccumulation, and toxicity (Konçe et al., 2019; Xie et al., 2022); these characteristics play a particularly important role in the drug development process (Bergazin et al., 2021). Accurate prediction of the pKa value of organic compounds is highly important in numerous fields, especially in the development of new drugs (Xiong et al., 2022; Zhang et al., 2022). However, accurate prediction of the pKa for drug-like molecules is also a tremendous challenge in chemistry (Zhang et al., 2022).

Due to its high-resolution ratio, selectivity, and reproducibility, RP-HPLC is the most extensive and central technique in the analysis and separation of a wide range of compounds and the study of the pKa values of drug molecules (D'Archivio, 2019; Yılmaz Ortak and Cubuk Demiralay, 2019). Apart from molecular structure, numerous factors in chromatographic analysis programs have an important influence on retention time, such as the pH of the mobile phase, column temperature, mobile phase composition, and type of chromatographic column (Huang et al., 2019; Tsui et al., 2019; Annadi et al., 2022; Shi et al., 2022). The chromatographic conditions can be adjusted and optimized to achieve satisfactory separation of mixtures and symmetric peak shapes. Furthermore, an increasing number of studies have reported the combined effect of two or more factors on the retention time (Phyo et al., 2018; Biancolillo et al., 2020; Kaczmarski and Chutkowski, 2021; Yilmaz, 2021). Comprehensive models that consider the influence of different chromatographic conditions are more accurate in predicting the retention times of compounds. However, previous studies have generally predicted the chromatographic retention or lipophilicity by using the quantitative structure–retention relationship (QSRR) models (Yang X. et al., 2020; Fouad et al., 2022; Xu et al., 2023). The QSRR models mainly focus on the molecular descriptors of the solutes, with less emphasis on the influence of different chromatographic conditions. Recently, models based on empirical or semiempirical equations and thermodynamic properties have rarely been reported to investigate the simultaneous effect of diverse chromatographic conditions on retention.

Herein, this study aims to provide multiparameter models that combine the effects of pH, temperature (T), organic modifier composition (φ), and polarity ( $P_{m}^{N}$ ) to predict the retention factors of amphoteric compounds (pentapeptides) under different chromatographic conditions in RP-HPLC. Initially, the retention factors of 57 pentapeptides under seven mobile phase pH values, three column temperatures, and four methanol compositions were measured *via* RP-HPLC. Then, the multiparameter models derived from the sigmoidal function, Van’t Hoff equation, and empirical formula between log k and the solvent polarity or solvent composition were built. Finally, the multiparameter models were evaluated by comparing the agreement between experimental k-values and predicted k-values.

## 2 Materials and methods

### 2.1 Chemicals

RP-HPLC-grade methanol was purchased from Fisher Scientific, and trifluoroacetic acid (TFA) was purchased from Fluka (Buchs, Switzerland). All other reagents were from Kermel (Tianjin, China); these included citric acid, sodium citrate, disodium hydrogen phosphate, and sodium dihydrogen phosphate.

The pentapeptides (HGRFG and NPNPT) were isolated from *Carapax Trionycis* and showed high anti-fibrosis activity (Supplementary Figure S1). The C- or N-termini of the pentapeptides of HGRFG and NPNPT were replaced with the remaining 19 amino acids to obtain the sequences of the derived pentapeptides. Then, the derived pentapeptides were synthesized by solid-phase synthesis (SPPS) and purified by RPLC. In this study, the sequences of the 57 analyzed pentapeptides are as follows: NPNPA, NPNPC, NPNPD, NPNPE, NPNPG, NPNPH, NPNPI, NPNPK, NPNPM, NPNPN, NPNPP, NPNPQ, NPNPR, NPNPS, NPNPT, NPNPV, NPNPY, APNPT, CPNPT, DPNPT, EPNPT, GPNPT, HPNPT, IPNPT, KPNPT, LPNPT, MPNPT, PPNPT, QPNPT, RPNPT, SPNPT, TPNPT, VPNPT, YPNPT, HGRFA, HGRFD, HGRFE, HGRFG, HGRFH, HGRFK, HGRFN, HGRFQ, HGRFR, HGRFS, HGRFT, AGRFG, DGRFG, EGRFG, GGRFG, KGRFG, NGRFG, PGRFG, QGRFG, RGRFG, SGRFG, TGRFG, and VGRFG.

### 2.2 Instruments

RP-HPLC was conducted *via* a Shimadzu Prominence LC-2030 Plus (Kyoto, Japan) instrument equipped with a SIL-20AC autosampler and two LC-20AD pumps. An SPD-20AV dual-wavelength detector at 215 nm and 254 nm was used to detect the pentapeptides. Instrument control, data acquisition, and processing were performed with LabSolutions software for RP-HPLC. A Shimadzu Shim-pack GIST C18 4.6 × 250 mm i. d., 5 μm particle size column was used as the stationary phase and was stable within the pH range of 1–10.

A PHS-25 pH meter purchased from INESA (Shanghai, China) was used to measure the pH values, combined with an E-201F-type composite electrode. Potassium hydrogen phthalate, mixed phosphate, and sodium tetraborate from INESA (Shanghai, China) were used for electrode calibration.

### 2.3 Chromatographic procedure

Mobile phases were prepared with water (A)–methanol (B) components, degassed, and mixed online. The pentapeptides were analyzed under isocratic elution of organic solvent B. The analysis procedures were, respectively, as follows: a: 8–14 v/v (increment 2 v/v) (NPNPA, NPNPD, NPNPE, NPNPG, NPNPH, NPNPK, NPNPN, NPNPQ, NPNPR, NPNPS, NPNPT, APNPT, DPNPT, EPNPT, GPNPT, HPNPT, KPNPT, PPNPT, RPNPT, SPNPT, and TPNPT); b: 20–26 v/v (increment 2 v/v) (HGRFA, HGRFD, HGRFE, HGRFG, HGRFH, HGRFK, HGRFN, HGRFQ, HGRFR, HGRFS, HGRFT, AGRFG, DGRFG, EGRFG, GGRFG, KGRFG, NGRFG, PGRFG, QGRFG, RGRFG, SGRFG, TGRFG, VGRFG, NPNPC, NPNPI, NPNPM, NPNPP, NPNPV, NPNPY, CPNPT, IPNPT, LPNPT, MPNPT, QPNPT, VPNPT, and YPNPT). The retention times were separately obtained at temperatures of 25°C, 35°C, and 45°C. The aqueous phase was prepared at 25°C by diluting stock solutions of buffer salt.

The parameters pHWWW and pHWWS of the RP-HPLC mobile phase were associated with the chromatographic retention of ionizable compounds through their thermodynamic acid-base constants in the methanol–water mixture. The pHWWW and pHWWS values were recorded before and after mixing water with the organic phase after the electrode was calibrated with the pH calibration solution at the working temperature, and the pHSSS values were calculated according to Eqs 2, 3. The pHWWS and pHSSS values at different temperatures and organic modifier compositions are shown in Table 1. All pH values are named according to IUPAC nomenclature.

**TABLE 1:** pHWWS and pHSSS in the mobile phase of high-performance liquid chromatography.

pHWWS													
Aqueous buffer		25°C				35°C				45°C			
		8%	10%	12%	14%	8%	10%	12%	14%	8%	10%	12%	14%
A	13.46 mM TFA	1.73	1.75	1.75	1.76	1.75	1.77	1.77	1.78	1.87	1.89	1.89	1.89
B	1.08 mM H_3_Cit+0.04 mM Na_3_Cit	3.07	3.09	3.10	3.15	3.09	3.12	3.13	3.14	3.12	3.16	3.18	3.19
C	1.92 mM H_3_Cit+0.31 mM Na_3_Cit	4.02	4.03	4.07	4.11	4.02	4.06	4.08	4.10	4.07	4.09	4.12	4.13
D	0.74 mM H_3_Cit+0.26 mM Na_3_Cit	5.07	5.09	5.12	5.12	5.05	5.09	5.13	5.14	5.05	5.11	5.14	5.19
E	0.55 mM Na_2_HPO_4_+12.51 mM NaH_2_PO_4_	6.04	6.08	6.15	6.18	6.03	6.08	6.13	6.15	6.04	6.08	6.13	6.17
F	1.36 mM Na_2_HPO_4_+2.63 mM NaH_2_PO_4_	7.09	7.12	7.18	7.24	7.08	7.10	7.15	7.17	7.02	7.09	7.15	7.18
G	2.11 mM Na_2_HPO_4_+1.02 mM NaH_2_PO_4_	8.08	8.15	8.17	8.20	8.04	8.10	8.11	8.21	8.00	8.03	8.07	8.12
Aqueous buffer		25°C				35°C				45°C			
		20%	22%	24%	26%	20%	22%	24%	26%	20%	22%	24%	26%
A	13.46 mM TFA	1.76	1.76	1.77	1.79	1.79	1.80	1.81	1.84	1.91	1.94	1.97	1.98
B	1.08 mM H_3_Cit+0.04 mM Na_3_Cit	3.17	3.18	3.21	3.23	3.21	3.21	3.23	3.23	3.22	3.24	3.27	3.29
C	1.92 mM H_3_Cit+0.31 mM Na_3_Cit	4.17	4.19	4.21	4.25	4.17	4.22	4.25	4.30	4.21	4.24	4.26	4.31
D	0.74 mM H_3_Cit+0.26 mM Na_3_Cit	5.25	5.27	5.31	5.35	5.22	5.26	5.32	5.36	5.27	5.33	5.37	5.41
E	0.55 mM Na_2_HPO_4_+12.51 mM NaH_2_PO_4_	6.27	6.31	6.38	6.41	6.24	6.29	6.34	6.38	6.27	6.33	6.37	6.42
F	1.36 mM Na_2_HPO_4_+2.63 mM NaH_2_PO_4_	7.32	7.37	7.41	7.46	7.29	7.33	7.40	7.41	7.30	7.34	7.40	7.45
G	2.11 mM Na_2_HPO_4_+1.02 mM NaH_2_PO_4_	8.33	8.40	8.43	8.45	8.30	8.33	8.38	8.45	8.25	8.31	8.35	8.36

pHSSS													
Aqueous buffer		25°C				35°C				45°C			
		8%	10%	12%	14%	8%	10%	12%	14%	8%	10%	12%	14%
A	13.46 mM TFA	1.72	1.74	1.74	1.74	1.74	1.76	1.76	1.76	1.86	1.88	1.88	1.87
B	1.08 mM H_3_Cit+0.04 mM Na_3_Cit	3.06	3.08	3.09	3.13	3.08	3.11	3.12	3.12	3.11	3.15	3.17	3.17
C	1.92 mM H_3_Cit+0.31 mM Na_3_Cit	4.01	4.02	4.06	4.09	4.01	4.05	4.07	4.08	4.06	4.08	4.11	4.11
D	0.74 mM H_3_Cit+0.26 mM Na_3_Cit	5.06	5.08	5.11	5.10	5.04	5.08	5.12	5.12	5.04	5.10	5.13	5.17
E	0.55 mM Na_2_HPO_4_+12.51 mM NaH_2_PO_4_	6.03	6.07	6.14	6.16	6.02	6.07	6.12	6.13	6.03	6.07	6.12	6.15
F	1.36 mM Na_2_HPO_4_+2.63 mM NaH_2_PO_4_	7.08	7.11	7.17	7.22	7.07	7.09	7.14	7.15	7.01	7.08	7.14	7.16
G	2.11 mM Na_2_HPO_4_+1.02 mM NaH_2_PO_4_	8.07	8.14	8.16	8.18	8.03	8.09	8.10	8.19	7.99	8.02	8.06	8.10
Aqueous buffer		25°C				35°C				45°C			
		20%	22%	24%	26%	20%	22%	24%	26%	20%	22%	24%	26%
A	13.46 mM TFA	1.73	1.73	1.73	1.75	1.76	1.77	1.77	1.80	1.88	1.91	1.93	1.94
B	1.08 mM H_3_Cit+0.04 mM Na_3_Cit	3.14	3.15	3.17	3.19	3.18	3.18	3.19	3.19	3.19	3.21	3.23	3.25
C	1.92 mM H_3_Cit+0.31 mM Na_3_Cit	4.14	4.16	4.17	4.21	4.14	4.19	4.21	4.26	4.18	4.21	4.22	4.27
D	0.74 mM H_3_Cit+0.26 mM Na_3_Cit	5.22	5.24	5.27	5.31	5.19	5.23	5.28	5.32	5.24	5.30	5.33	5.37
E	0.55 mM Na_2_HPO_4_+12.51 mM NaH_2_PO_4_	6.24	6.28	6.34	6.37	6.21	6.26	6.30	6.34	6.24	6.30	6.33	6.38
F	1.36 mM Na_2_HPO_4_+2.63 mM NaH_2_PO_4_	7.29	7.34	7.37	7.42	7.26	7.30	7.36	7.37	7.27	7.31	7.36	7.41
G	2.11 mM Na_2_HPO_4_+1.02 mM NaH_2_PO_4_	8.30	8.37	8.39	8.41	8.27	8.30	8.34	8.41	8.22	8.28	8.31	8.32

The solutes were initially dissolved in pure water at a concentration lower than 1 mg/ml and then filtered through a 0.45 µm nylon mobile phase filter. The flow rate of the chromatographic system was maintained at 1.0 ml/min, and the injection volume was 10 μL.

### 2.4 Data statistics and analysis

Both non-linear regressions of the chromatographic retention factor k with pH or other parameters in the multiparameter equation and linear regression were performed using MATLAB R2019a (Version 9.6.0; MathWorks, Natick, MA, USA).

## 3 Theory

### 3.1 Influence of pH

The theoretical sigmoidal function of pH and retention factor k derived from chromatographic theory has been widely used for ionizable compounds (Konçe et al., 2019). Previous studies have verified the wide applicability of ionizable compounds in chromatographic analysis (Yang et al., 2018; Soriano-Meseguer et al., 2019). Thus, according to Equation 1, the acid–base equilibrium determined by the acidity constant Ka , i.e., the retention factor of the monoprotic acid solute, depends on the pH of the mobile phase.

$$k=\frac{k_{HA}+k_{A}10^{\left(pH-pKa\right)}}{1+10^{\left(pH-pKa\right)}},$$ (1)

where $k_{HA}$ and $k_{A}$ represent the limiting retention factors of the protonated and dissociated forms of the analyte, respectively. The pKa is the acid-base equilibrium constant of the solute at a given mobile phase composition and temperature. Most important, the pH here is pHSSS because this has been widely studied and it has been found that the fitting ability of this equation can be ensured only when the pH and pKa correspond to the real values. pHSSS can be calculated using Equation 2, as follows (Alvarez-Segura et al., 2019; Soriano-Meseguer et al., 2019):

pHSSS=pHWWS−δ, (2)

where the empirical formula could be used to estimate δ from solvent composition as follows:

$$\delta =\frac{0.09\varphi_{MeOH}-0.11\varphi_{MeOH}^{2}}{1-3.15\varphi_{MeOH}+3.51\varphi_{MeOH}^{2}-1.35\varphi_{MeOH}^{3}},$$ (3)

where $\varphi_{MeOH}$ is the volume fraction of methanol in the mixed mobile phase.

### 3.2 Influence of temperature

For a reversible process of chromatographic analysis, the dissociation of the analyte and buffer and the solute migration during retention, which could be affected by the column temperature change, are applicable to the Van’t Hoff equation (Faisal et al., 2018; Marchetti et al., 2019; Yuan et al., 2020) as follows:

$$logk=-\frac{∆H^{0}}{2.3RT}+\frac{∆S^{0}}{2.3R}+\log\Phi ,$$ (4)

where $∆H^{0}$ and $∆S^{0}$ represent the D-values of enthalpy and entropy, respectively, when the solute is transferred from the mobile to the stationary phase; R is the gas constant; and Φ represents the phase ratio. Here, we assume that the enthalpy and entropy of this equilibrium process are definite constants in the studied temperature range and that the phase ratio Φ is free of the effect.

Similarly, for the reversible process $HA⇌H^{+}+A^{-}$ , the acid dissociation constant pKa is the negative logarithm of the equilibrium constant Ka ; thus, the correlation between pKa and temperature is described by the Van’t Hoff equation as follows:

$$pK_{a}=\frac{∆H_{a}^{0}}{2.3RT}-\frac{∆S_{a}^{0}}{2.3R}-\log\Phi ,$$ (5)

where $∆H_{a}^{0}$ and $∆S_{a}^{0}$ are the changes in enthalpy and entropy caused by solute dissociation, respectively.

### 3.3 Simultaneous influence of pH and temperature

Introducing Eqs 4, and 5 into Eq. 1 produces the following equation:

$$k=\frac{10^{a+\frac{b}{T}}+10^{c+\frac{d}{T}}10^{\left(pH-e-\frac{f}{T}\right)}}{1+10^{\left(pH-e-\frac{f}{T}\right)}},$$ (6)

where the fitting parameter includes the thermodynamic quantities related to the dissociation and transformation of the analyzed compound, i.e., the function composed of these quantities: $a=\left(\frac{△S_{HA}^{0}}{2.3R}+\log\Phi \right),b=\frac{-△H_{HA}^{0}}{2.3R},c=\left(\frac{△S_{A}^{0}}{2.3R}+\log\Phi \right),d=\frac{-△H_{A}^{0}}{2.3R},e=\left(\frac{△S_{a}^{0}}{2.3R}+\log\Phi \right),f=\frac{-△H_{a}^{0}}{2.3R}$ , and the subscripts HA and A apart represent the protonation and deprotonation forms of acid–base solutes.

### 3.4 Influence of the organic modifier composition

The composition of the mobile phase is the main variable used to optimize retention and selectivity in RP-HPLC. The Soczewiński–Wachtmeister equation is commonly used to describe the relationship between k and the change in mobile phase (Flieger et al., 2020; Lin et al., 2022).

$$logk=-S\varphi +\log k_{W},$$ (7)

where $\log k_{W}$ is the intercept and represents the retention coefficient of solute in pure water, S is the slope of the equation and represents the sensitivity of solute molecules to solvent strength, and φ is the volume fraction of organic modifier in the mobile phase.

Considering the influence of the polarity of the solute, stationary phase, and mobile phase on k, another linear model was proposed to accurately describe k, which represents the linear relationship between the retention rate and the polarity of the eluent (Gisbert-Alonso et al., 2021; Zhu et al., 2022); the relationship is as follows:

$$logk={\left(logk\right)}_{0}-p\left(P_{m}^{N}-P_{s}^{N}\right),$$ (8)

where p is the parameter describing the polarity of the solute, $P_{m}^{N}$ and $P_{s}^{N}$ are the standard polarity parameters of the mobile and stationary phases, and ${\left(logk\right)}_{0}$ is the retention factor when the polarity of the mobile phase is the same as that of the stationary phase. Numerous experimental studies have shown that for a specific column and water-methanol mobile phase, the parameters of Eq. 8 can be obtained by measuring the retention rate logk of a group of solutes, where ${\left(logk\right)}_{0}$ and $P_{s}^{N}$ are the system constants. In addition, the logk value in the model is linear with respect to $P_{m}^{N}$ over the entire range of water-methanol mobile phase compositions (0–100%) and intersects at a common extrapolation point in the majority of cases.

For the water–methanol system, the relationship between $P_{m}^{N}$ and φ is as follows (Zhu et al., 2022):

$$P_{m}^{N}=1.00-\frac{1.33\varphi }{1+0.47\varphi }.$$ (9)

To eliminate the limit of all logk and $P_{m}^{N}$ lines that must cross at the same point, a deformation of Eq. 8 is proposed to represent the solute in the model by two descriptors (q and p) (den Uijl et al., 2021) and shown as follows:

$$logk=q+pP_{m}^{N},$$ (10)

where fitting parameters concerning the solute are twice those before, which improves the accuracy of model prediction.

The mobile phase composition affects not only the retention rate but also the ionization degree of the acid–base solute, and the addition of an organic solvent to the aqueous solution containing ionizable compounds changes the value of pKa . For a specific solute, the solute parameters are constant, and pKa only depends on the solvent properties or temperature, whereas when using mixed solvents (such as the mobile phase), the solvent properties and pKa change monotonically with the mobile phase composition. Therefore, the relationship between the pKa value and the solvent volume fraction can usually be expressed as follows:

pKa=E+Fφ. (11)

Similarly, the relationship between pKa and mobile phase polarity parameters can be expressed as follows:

$$pKa=E+FP_{m}^{N}.$$ (12)

### 3.5 Simultaneous influence of pH and organic modifier composition

Based on the aforementioned analysis, combined with the model of pH and different mobile phase compositions, the six-parameter model is obtained as follows:

$$k=\frac{10^{A+BX}+10^{C+DX}10^{\left(pH-E-FX\right)}}{1+10^{\left(pH-E-FX\right)}},$$ (13)

and

$$\log k_{HA}=A+BX,$$ (14)

$$\log k_{A}=C+DX,$$ (15)

pKa=E+FX, (16)

where X is the variable describing the change in the mobile phase, representing φ or $P_{m}^{N}$ in the two-parameter solvent model. Parameters A, B, C, D, E, and F of the model have a simple chemical interpretation. If X is the volume fraction φ of the organic modifier in the mobile phase, then A, C, and E are the extrapolation of the logarithm of k in acidic and alkaline forms and the pKa of the compound in pure water, respectively, while B, D, and F are the changes in these parameters from pure water to pure organic solvent. Similarly, if the polarity parameter $P_{m}^{N}$ is fitted as the X variable, then A, C, and E are the reserves in a non-polar medium, and the pKa , and B, D, and F are the changes from the medium to pure water (defined as $P_{m}^{N}$ =1).

## 4 Results and discussion

Small-molecular oligopeptides commonly participate in multiple physiological and pathological processes, including the transmission of signals and the regulation of immune and inflammatory responses (Yang J. et al., 2020; Gao et al., 2022). RP-HPLC is a common approach to separate small-molecular peptides by adjusting the chromatographic conditions (Liu et al., 2022). The retention factors of 57 ionizable solute derivatives of pentapeptides of NPNPT and HGRFG were determined at seven mobile pH values, four mobile phase compositions, and three column temperatures (84 data points for each solute). Some comparative chromatograms are shown in Supplementary Figure S2. We selected the pentapeptides with high polarity, similar retention, and a similar chemical structure for the model’s establishment and evaluation, and the 57 pentapeptides could be divided into five groups according to the acidity or basicity of the isoelectric points and the polarity of the pentapeptides. The groups included the following: (1) 8%–14% methanol acid pentapeptides: NPNPD, NPNPE, DPNPT, and EPNPT; (2) 8%–14% methanol basic pentapeptides: NPNPK, NPNPR, KPNPT, and RPNPT; (3) 8%–14% methanol neutral pentapeptides: NPNPA, NPNPG, NPNPH, NPNPN, NPNPQ, NPNPS, APNPT, GPNPT, HPNPT, NPNPT, PPNPT, SPNPT, and TPNPT; (4) 20%–26% methanol basic pentapeptides: HGRFA, HGRFG, HGRFH, HGRFK, HGRFN, HGRFQ, HGRFR, HGRFS, HGRFT, AGRFG, GGRFG, KGRFG, NGRFG, PGRFG, QGRFG, RGRFG, SGRFG, TGRFG, and VGRFG; and (5) 20%–26% methanol neutral pentapeptides: NPNPC, NPNPI, NPNPM, NPNPP, NPNPV, NPNPY, CPNPT, IPNPT, LPNPT, MPNPT, QPNPT, VPNPT, YPNPT, HGRFD, HGRFE, DGRFG, and EGRFG.

### 4.1 Function of the retention factor k and pH

**FIGURE 1:**
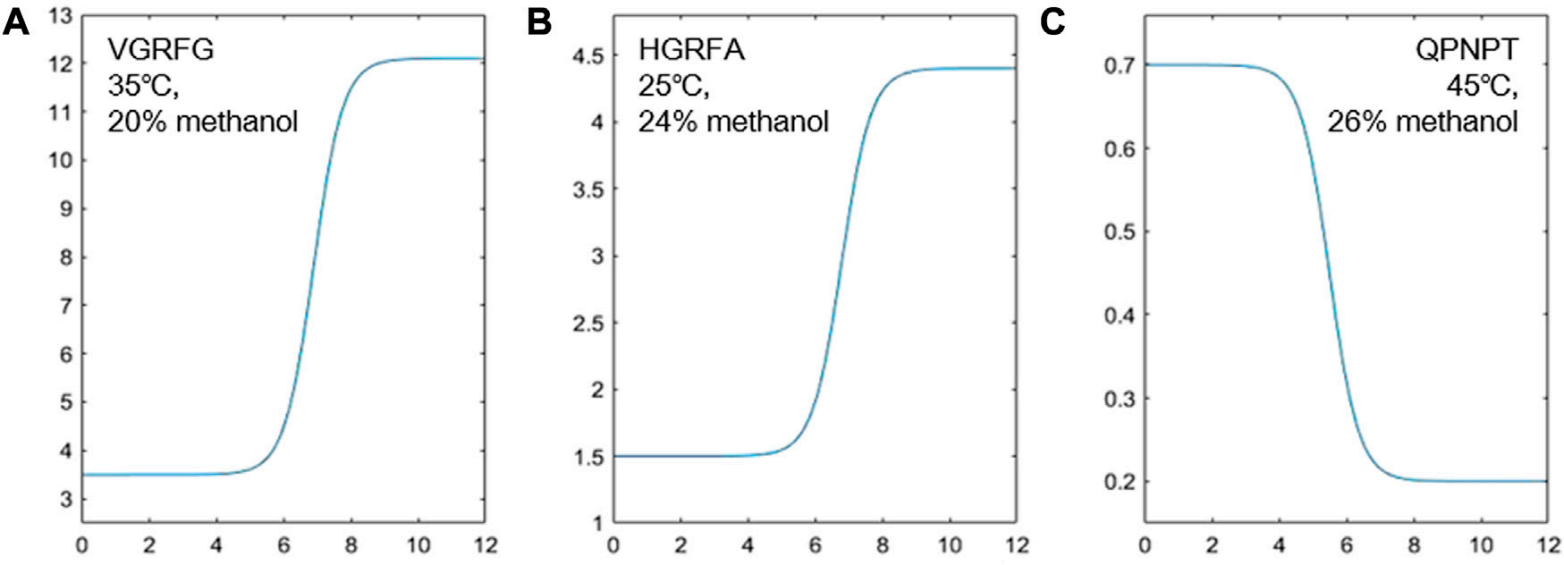
Fitting of the experimental retention and different pH values of VGRFG at 35°C and 20% methanol **(A)**, HGRFA at 25°C and 24% methanol **(B)**, and QPNPT at 45°C and 26% methanol **(C)**.

The pH of the mobile phase is one of the critical factors affecting the retention of compounds in chromatography due to its interference with the ionization efficiency and change in the protonation of analytes (Fan et al., 2022; Guo et al., 2023). Because the increased or decreased degree of chromatographic retention for compounds was different with the change in pH, adjusting the pH of the mobile phase was capable of separating the compounds with similar structures or confirming the absence of unrelated impurities (Fan et al., 2022; Tengattini et al., 2022). MATLAB R2019a was used to fit the S-curve of different pH values and the experimental retention factors (k) under the same temperature and the same mobile phase composition. From Eq. 1, we obtained the parameters $k_{HA}$ , $k_{A}$ , and $pK_{a}$ , as shown in Table 2 Table 3 Table 4. With the analysis of k and their corresponding pH values, the $pK_{a}$ determination by the inflection point in the curve was the most established method for its calculation for a compound (Numviyimana et al., 2019). However, the retention times in RP-HPLC were generally short owing to the high polarity of solutes, leading to unsatisfactory fitting outcomes of the S-curve and predictions for inflection point $pK_{a}$ . Only the $pK_{a}$ -values of the acid and basic compounds from fitting were close to the software calculation results (Figure 1), consistent with the study by Rook et al. (2021) on particular sidechains.

**TABLE 2:** Parameter results of pentapeptides at 25°C.

Pentapeptide sequence	8.0%			10.0%			12.0%			14.0%		
	$k_{HA}$	$k_{A}$	$pK_{a}$	$k_{HA}$	$k_{A}$	$pK_{a}$	$k_{HA}$	$k_{A}$	$pK_{a}$	$k_{HA}$	$k_{A}$	$pK_{a}$
APNPT	52.5	1.8	1.0	40.9	1.2	0.9	13.5	0.9	1.3	4.5	0.7	1.9
DPNPT	10.8	0.9	2.6	6.8	0.6	2.6	4.7	0.5	2.6	3.2	0.4	2.7
EPNPT	15.7	0.9	2.4	9.2	0.5	2.5	6.2	0.3	2.5	4.0	0.2	2.6
GPNPT	19.9	1.4	1.4	18.0	1.0	1.2	6.2	0.8	1.7	82.8	0.6	0.1
HPNPT	69.6	3.4	0.9	41.4	2.1	0.9	32.0	1.4	0.8	20.8	1.0	0.8
KPNPT	37.0	1.0	0.9	24.4	0.7	0.9	90.0	0.6	0.1	12.1	0.4	0.9
NPNPT	30.0	1.5	1.2	27.7	1.0	1.0	11.3	0.7	1.3	88.8	0.5	0.1
PPNPT	90.4	3.2	1.0	53.8	2.1	1.0	17.6	1.4	1.4	27.1	1.0	0.9
RPNPT	74.5	1.9	1.0	53.6	1.3	0.9	33.4	1.0	0.9	22.0	0.7	0.9
SPNPT	13.4	1.5	1.8	19.4	1.0	1.2	8.5	0.7	1.5	91.3	0.5	0.1
TPNPT	62.7	2.5	1.1	47.2	1.6	1.0	37.4	1.1	0.9	10.6	0.8	1.4
NPNPA	20.1	1.5	1.4	17.9	1.0	1.2	6.1	0.7	1.7	81.4	0.6	0.1
NPNPD	5.5	0.5	2.5	3.5	0.3	2.6	2.4	0.2	2.7	1.7	0.1	2.9
NPNPE	9.5	0.6	2.5	6.0	0.4	2.5	4.0	0.3	2.5	2.7	0.2	2.7
NPNPG	29.5	1.1	0.8	109.0	0.8	0.0	48.4	0.6	0.2	1.7	0.4	2.4
NPNPH	31.8	1.2	0.8	92.8	0.8	0.1	61.4	0.6	0.1	92.0	0.4	-0.2
NPNPK	28.0	0.8	0.8	85.4	0.5	0.1	18.9	0.4	0.6	65.0	0.3	-0.1
NPNPN	98.5	0.9	0.0	44.0	0.6	0.2	10.9	0.4	0.7	4.6	0.3	1.0
NPNPQ	10.1	1.0	1.4	83.7	0.7	0.1	2.2	0.5	2.2	1.4	0.3	2.7
NPNPR	44.4	1.4	0.9	27.0	1.0	0.9	95.3	0.7	0.1	53.7	0.5	0.2
NPNPS	10.4	0.9	1.4	91.3	0.6	0.1	2.3	0.4	2.3	1.5	0.3	2.6

Pentapeptide sequence	20.0%			22.0%			24.0%			26.0%		
	$k_{HA}$	$k_{A}$	$pK_{a}$	$k_{HA}$	$k_{A}$	$pK_{a}$	$k_{HA}$	$k_{A}$	$pK_{a}$	$k_{HA}$	$k_{A}$	$pK_{a}$
HGRFA	47.7	3.2	0.9	42.0	2.4	0.8	1.5	4.4	6.8	1.2	3.7	6.9
HGRFD	57.7	1.2	0.9	42.3	0.9	0.9	29.1	0.7	0.9	21.9	0.6	0.9
HGRFE	55.0	1.1	0.9	39.9	0.9	0.9	27.3	0.7	0.9	25.0	0.6	0.8
HGRFG	41.0	2.5	0.9	29.8	2.0	0.9	1.2	3.6	6.8	1.0	2.9	6.8
HGRFH	1.4	9.1	6.7	1.0	6.7	6.7	0.8	5.4	6.8	0.6	4.3	6.8
HGRFK	1.2	5.3	6.6	1.0	4.1	6.6	0.7	3.7	6.6	0.5	3.3	6.6
HGRFN	1.4	4.5	6.7	1.1	3.4	6.7	0.8	2.8	6.7	0.7	2.3	6.8
HGRFQ	35.0	2.1	0.8	1.3	3.7	6.8	1.0	3.0	6.8	0.8	2.4	6.8
HGRFR	1.6	7.8	6.6	1.2	5.9	6.6	0.8	5.2	6.6	0.6	4.6	6.6
HGRFS	34.0	2.1	0.9	25.2	1.6	0.9	1.0	3.0	6.7	0.8	2.5	6.7
HGRFT	53.0	2.8	0.9	38.2	2.2	0.9	29.0	1.8	0.8	1.1	3.3	6.8
AGRFG	40.2	2.1	0.9	30.5	1.7	0.9	26.8	1.4	0.8	24.8	1.2	0.7
DGRFG	42.5	1.5	0.9	32.4	1.2	0.9	28.9	1.0	0.8	23.0	0.8	0.8
EGRFG	49.3	1.5	0.9	37.0	1.2	0.9	26.9	1.0	0.9	25.7	0.8	0.8
GGRFG	32.2	1.7	0.9	29.8	1.4	0.8	26.0	1.2	0.7	85.3	1.1	0.0
KGRFG	38.0	2.3	0.9	1.4	4.8	6.5	1.0	4.5	6.6	0.7	4.2	6.6
NGRFG	38.0	1.6	0.9	35.0	1.3	0.8	25.2	1.1	0.8	110.0	0.9	0.0
PGRFG	2.8	8.7	7.0	2.2	6.9	7.0	30.2	2.0	0.8	1.3	4.9	7.0
QGRFG	44.2	1.8	0.9	40.7	1.5	0.8	30.1	1.2	0.8	23.6	1.0	0.8
RGRFG	45.9	1.6	1.0	40.5	1.3	0.9	32.0	1.2	0.8	23.5	1.0	0.8
SGRFG	38.6	1.6	0.9	35.7	1.3	0.8	25.8	1.1	0.8	20.4	0.9	0.8
TGRFG	41.0	2.1	0.9	30.7	1.7	0.9	26.6	1.4	0.8	24.5	1.2	0.7
VGRFG	4.6	15.0	7.1	3.6	11.2	7.0	2.6	8.7	7.0	2.0	6.9	7.0
NPNPC	21.9	0.4	1.5	17.9	0.4	1.3	13.1	0.3	1.2	3.4	0.2	1.9
NPNPI	55.2	2.6	1.1	40.8	2.0	1.1	45.0	1.6	0.9	10.2	1.2	1.7
NPNPM	11.8	1.2	1.6	15.6	1.0	1.2	6.2	0.7	1.7	90.8	0.6	0.1
NPNPP	2.9	0.4	2.5	2.1	0.3	2.6	1.5	0.2	2.8	1.2	0.2	2.8
NPNPV	17.3	1.0	1.2	8.4	0.8	1.5	99.1	0.6	0.1	2.7	0.5	2.3
NPNPY	18.7	1.5	1.4	19.2	1.1	1.2	10.5	0.9	1.4	4.0	0.7	2.1
CPNPT	2.4	0.5	2.3	1.7	0.4	2.5	1.3	0.3	2.6	1.0	0.2	2.9
IPNPT	52.3	2.5	0.9	35.4	1.9	0.9	30.9	1.4	0.8	22.1	1.1	0.8
LPNPT	59.0	3.0	1.0	48.1	2.3	0.9	34.4	1.7	0.9	30.2	1.3	0.8
MPNPT	39.9	1.7	0.9	34.0	1.3	0.8	24.7	1.0	0.8	80.9	0.8	0.1
QPNPT	2.0	0.5	4.5	1.4	0.3	5.2	1.1	0.2	5.4	0.9	0.1	5.6
VPNPT	27.0	1.0	0.8	83.5	0.8	0.1	50.1	0.6	0.2	2.2	0.5	1.9
YPNPT	50.9	2.3	0.9	33.8	1.7	0.9	29.5	1.2	0.8	20.9	0.9	0.8

**TABLE 3:** Parameter results of pentapeptides at 35°C.

Pentapeptide sequence	8.0%			10.0%			12.0%			14.0%		
	$k_{HA}$	$k_{A}$	$pK_{a}$	$k_{HA}$	$k_{A}$	$pK_{a}$	$k_{HA}$	$k_{A}$	$pK_{a}$	$k_{HA}$	$k_{A}$	$pK_{a}$
APNPT	49.0	1.7	0.9	33.0	1.2	0.9	15.5	0.8	1.1	87.5	0.6	0.1
DPNPT	8.0	0.8	2.6	5.3	0.6	2.6	3.5	0.3	2.8	2.5	0.2	2.8
EPNPT	10.9	0.8	2.5	7.1	0.5	2.5	4.6	0.3	2.6	3.2	0.2	2.6
GPNPT	17.7	1.3	1.3	25.2	0.9	0.9	93.6	0.7	0.1	2.8	0.5	2.0
HPNPT	49.0	2.7	0.9	31.0	1.7	0.9	24.4	1.1	0.8	72.4	0.8	0.1
KPNPT	30.5	0.9	0.9	25.0	0.7	0.8	21.2	0.5	0.7	87.5	0.4	-0.1
NPNPT	16.1	1.3	1.4	26.1	0.9	0.9	99.3	0.6	0.1	3.4	0.4	1.8
PPNPT	66.7	2.9	1.0	50.8	1.9	0.9	32.3	1.3	0.9	25.6	1.0	0.8
RPNPT	53.8	1.6	1.0	40.7	1.1	0.9	25.6	0.8	0.9	99.2	0.5	0.1
SPNPT	10.3	1.3	1.8	13.2	0.9	1.3	4.1	0.6	2.0	3.8	0.5	1.7
TPNPT	57.9	2.2	1.0	18.8	1.5	1.4	29.7	1.0	0.9	6.6	0.7	1.6
NPNPA	13.6	1.3	1.5	10.5	0.9	1.4	93.8	0.7	0.1	2.5	0.5	2.2
NPNPD	4.0	0.4	2.6	2.7	0.2	2.7	1.9	0.2	2.7	1.5	0.1	2.6
NPNPE	6.5	0.5	2.6	4.3	0.3	2.6	3.0	0.2	2.6	2.1	0.1	2.7
NPNPG	23.1	0.9	0.8	60.0	0.6	0.2	5.9	0.4	1.2	6.3	0.3	1.0
NPNPH	24.7	0.9	0.8	76.4	0.6	0.1	86.3	0.4	-0.1	79.0	0.3	-0.2
NPNPK	100.9	0.7	0.1	19.9	0.4	0.7	80.2	0.3	-0.1	61.0	0.2	-0.1
NPNPN	63.5	0.7	0.1	2.0	0.5	2.0	5.4	0.3	1.0	1.9	0.2	1.5
NPNPQ	20.9	0.9	0.8	52.7	0.6	0.2	1.9	0.4	2.1	5.3	0.3	1.0
NPNPR	32.2	1.1	0.9	25.8	0.7	0.8	78.0	0.5	0.1	83.5	0.4	-0.1
NPNPS	12.6	0.8	1.1	58.5	0.5	0.2	11.2	0.4	0.8	5.7	0.3	1.0

Pentapeptide sequence	20.0%			22.0%			24.0%			26.0%		
	$k_{HA}$	$k_{A}$	$pK_{a}$	$k_{HA}$	$k_{A}$	$pK_{a}$	$k_{HA}$	$k_{A}$	$pK_{a}$	$k_{HA}$	$k_{A}$	$pK_{a}$
HGRFA	37.2	2.4	0.9	27.4	1.9	0.9	1.2	3.3	6.7	1.0	2.7	6.8
HGRFD	41.3	0.8	0.9	30.7	0.7	0.9	22.8	0.6	0.9	12.1	0.5	1.1
HGRFE	41.0	0.8	0.9	30.2	0.7	0.9	23.0	0.5	0.9	12.2	0.4	1.1
HGRFG	31.8	1.8	0.9	29.3	1.4	0.8	25.9	1.2	0.7	96.0	0.9	0.0
HGRFH	1.1	6.1	6.7	0.8	4.7	6.7	0.6	3.6	6.7	0.5	2.8	6.7
HGRFK	1.0	3.6	6.6	0.8	3.0	6.6	0.6	2.6	6.6	0.5	2.2	6.6
HGRFN	1.0	3.2	6.7	0.8	2.5	6.7	0.6	2.0	6.7	0.5	1.6	6.7
HGRFQ	1.2	3.4	6.7	0.9	2.7	6.7	0.7	2.1	6.8	54.9	0.7	0.1
HGRFR	1.1	5.0	6.5	0.9	4.3	6.6	0.6	3.7	6.6	0.5	3.0	6.6
HGRFS	31.1	1.5	0.8	23.3	1.2	0.8	100.6	0.9	0.0	59.2	0.8	0.1
HGRFT	38.8	2.1	0.9	28.2	1.7	0.9	25.4	1.3	0.8	23.0	1.1	0.7
AGRFG	31.5	1.6	0.9	29.8	1.3	0.8	22.6	1.1	0.8	104.4	0.9	0.0
DGRFG	32.7	1.1	0.9	25.5	0.9	0.9	24.7	0.7	0.8	91.3	0.6	0.1
EGRFG	37.9	1.1	0.9	29.1	0.9	0.9	22.7	0.7	0.9	102.7	0.6	0.1
GGRFG	31.2	1.3	0.8	24.0	1.1	0.8	104.0	0.9	0.0	65.6	0.8	0.1
KGRFG	29.7	1.7	0.9	1.0	3.7	6.6	0.7	3.2	6.6	0.6	2.9	6.6
NGRFG	36.0	1.2	0.8	27.7	1.0	0.8	21.7	0.8	0.8	78.5	0.7	0.1
PGRFG	2.1	7.6	7.0	1.6	6.2	7.0	1.3	5.1	7.0	1.0	4.1	6.9
QGRFG	41.0	1.4	0.8	31.7	1.2	0.8	24.6	1.0	0.8	93.0	0.8	0.1
RGRFG	41.6	1.2	0.9	30.2	1.1	0.9	27.5	0.9	0.8	91.8	0.8	0.1
SGRFG	30.4	1.2	0.9	28.6	1.0	0.8	22.5	0.8	0.8	76.7	0.8	0.1
TGRFG	32.2	1.6	0.9	30.1	1.3	0.8	22.7	1.1	0.8	103.3	0.9	0.0
VGRFG	3.5	12.1	6.9	2.6	9.3	6.9	2.0	7.2	6.9	1.6	5.7	6.9
NPNPC	41.6	0.4	0.9	32.7	0.3	0.8	6.0	0.2	1.5	3.7	0.2	1.6
NPNPI	17.2	2.3	1.8	34.9	1.8	1.1	12.4	1.4	1.6	16.1	1.1	1.3
NPNPM	14.4	1.0	1.3	7.3	0.8	1.6	99.0	0.6	0.1	3.8	0.5	1.8
NPNPP	2.3	0.3	2.6	1.8	0.3	2.5	1.5	0.2	2.3	1.3	0.1	2.2
NPNPV	12.6	0.9	1.3	5.2	0.7	1.8	86.2	0.6	0.1	2.6	0.4	2.2
NPNPY	8.3	1.1	1.8	10.3	0.9	1.4	5.4	0.7	1.7	3.1	0.5	2.1
CPNPT	6.9	0.5	1.1	1.8	0.4	2.0	1.2	0.3	2.3	1.0	0.2	2.3
IPNPT	41.7	2.4	0.9	29.4	1.8	0.9	30.0	1.4	0.7	20.0	1.1	0.8
LPNPT	56.0	2.9	0.9	39.4	2.2	0.9	28.2	1.6	0.9	27.0	1.3	0.8
MPNPT	38.4	1.5	0.8	28.3	1.1	0.8	20.1	0.9	0.8	74.9	0.7	0.1
QPNPT	1.6	0.4	4.8	1.2	0.2	5.3	1.0	0.2	4.4	0.8	0.2	4.4
VPNPT	21.9	1.0	0.8	70.2	0.8	0.1	42.4	0.6	0.2	12.0	0.5	0.7
YPNPT	34.9	1.8	0.9	30.0	1.3	0.8	20.8	1.0	0.8	77.4	0.7	0.1

**TABLE 4:** Parameter results of pentapeptides at 45°C.

Pentapeptide sequence	8.0%			10.0%			12.0%			14.0%		
	$k_{HA}$	$k_{A}$	$pK_{a}$	$k_{HA}$	$k_{A}$	$pK_{a}$	$k_{HA}$	$k_{A}$	$pK_{a}$	$k_{HA}$	$k_{A}$	$pK_{a}$
APNPT	43.0	1.5	1.0	33.6	1.1	0.9	22.8	0.8	0.9	71.7	0.6	0.2
DPNPT	6.8	0.7	2.6	4.3	0.5	2.7	2.9	0.3	2.9	2.0	0.2	3.0
EPNPT	9.1	0.7	2.5	5.6	0.5	2.6	3.6	0.3	2.7	2.6	0.2	2.7
GPNPT	38.1	1.1	0.9	10.2	0.8	1.4	101.9	0.6	0.1	2.2	0.5	2.2
HPNPT	43.4	2.1	0.9	32.8	1.4	0.8	21.7	0.9	0.8	55.5	0.6	0.2
KPNPT	28.9	1.0	0.9	23.0	0.7	0.8	60.0	0.5	0.2	17.5	0.4	0.6
NPNPT	39.7	1.1	0.9	10.7	0.7	1.4	85.3	0.5	0.2	3.9	0.4	1.6
PPNPT	66.0	2.7	1.0	41.0	1.8	1.0	38.0	1.3	0.8	24.6	1.0	0.8
RPNPT	56.0	1.5	0.9	34.9	1.0	0.9	22.5	0.7	0.9	79.0	0.6	0.1
SPNPT	10.7	1.1	1.7	11.0	0.8	1.4	107.6	0.6	0.1	2.7	0.4	2.0
TPNPT	57.8	1.9	1.0	18.5	1.3	1.4	29.9	0.9	0.9	8.1	0.7	1.4
NPNPA	26.7	1.1	1.1	10.6	0.8	1.4	103.0	0.6	0.1	2.1	0.5	2.3
NPNPD	3.6	0.4	2.5	2.2	0.2	2.8	1.6	0.1	2.8	1.2	0.1	2.8
NPNPE	5.6	0.5	2.5	3.4	0.2	2.7	2.4	0.2	2.7	1.7	0.1	2.8
NPNPG	25.0	0.8	0.8	62.2	0.6	0.2	2.1	0.4	2.0	6.4	0.3	1.0
NPNPH	24.0	0.8	0.8	60.3	0.5	0.2	99.5	0.4	-0.2	72.6	0.3	-0.2
NPNPK	107.1	0.6	0.1	24.0	0.4	0.6	78.8	0.3	-0.1	60.2	0.2	-0.1
NPNPN	58.3	0.6	0.2	1.9	0.4	2.0	5.5	0.3	1.0	1.0	0.2	2.1
NPNPQ	5.8	0.7	1.6	2.4	0.5	2.1	7.2	0.4	1.0	5.3	0.3	1.0
NPNPR	29.1	1.0	0.9	102.8	0.7	0.1	22.9	0.5	0.6	19.0	0.4	0.5
NPNPS	15.8	0.7	1.0	2.9	0.5	1.9	2.4	0.3	1.8	5.6	0.3	1.0

Pentapeptide sequence	20.0%			22.0%			24.0%			26.0%		
	$k_{HA}$	$k_{A}$	$pK_{a}$	$k_{HA}$	$k_{A}$	$pK_{a}$	$k_{HA}$	$k_{A}$	$pK_{a}$	$k_{HA}$	$k_{A}$	$pK_{a}$
HGRFA	1.3	4.6	6.7	1.1	3.6	6.7	0.8	2.9	6.7	0.7	2.4	6.8
HGRFD	28.7	0.7	0.9	22.6	0.6	0.9	11.9	0.5	1.1	11.2	0.4	1.0
HGRFE	29.6	0.7	0.9	23.1	0.6	0.9	12.1	0.5	1.1	11.3	0.4	1.0
HGRFG	1.1	3.6	6.7	0.9	2.9	6.8	26.2	1.0	0.8	0.5	1.9	6.8
HGRFH	0.7	4.9	6.7	0.6	3.8	6.7	0.5	3.0	6.7	0.4	2.5	6.8
HGRFK	0.6	5.0	6.7	0.5	4.1	6.8	0.4	3.4	6.8	0.3	3.0	6.8
HGRFN	0.7	2.9	6.7	0.5	2.3	6.7	0.4	1.9	6.7	0.3	1.6	6.8
HGRFQ	0.8	3.0	6.7	0.6	2.4	6.7	0.5	2.0	6.8	0.4	1.6	6.9
HGRFR	0.8	3.8	6.9	0.5	4.4	6.6	0.4	3.9	6.7	0.3	3.4	6.7
HGRFS	0.9	3.0	6.7	0.7	2.5	6.8	0.5	2.0	6.7	0.4	1.7	6.8
HGRFT	1.2	4.2	6.7	1.0	3.3	6.7	0.8	2.7	6.8	0.6	2.2	6.8
AGRFG	29.5	1.4	0.8	24.4	1.1	0.8	0.8	2.1	6.9	0.6	1.8	6.9
DGRFG	27.0	0.9	0.9	22.7	0.7	0.9	104.4	0.6	0.1	11.6	0.5	1.0
EGRFG	31.8	0.8	0.9	25.4	0.7	0.9	21.5	0.5	0.9	25.7	0.4	0.7
GGRFG	24.0	1.1	0.8	90.0	0.9	0.1	70.4	0.8	0.1	41.4	0.7	0.2
KGRFG	0.8	5.4	6.8	0.6	4.9	6.8	0.4	4.5	6.8	0.3	4.2	6.8
NGRFG	29.0	1.0	0.8	22.7	0.9	0.8	87.5	0.7	0.1	52.8	0.6	0.2
PGRFG	1.4	7.6	7.0	1.1	6.1	7.0	0.9	5.0	7.0	0.7	4.2	7.0
QGRFG	32.6	1.2	0.8	26.6	1.0	0.8	20.6	0.9	0.8	69.6	0.8	0.1
RGRFG	35.0	1.1	0.8	27.5	0.9	0.8	96.5	0.8	0.1	68.0	0.7	0.1
SGRFG	29.9	1.0	0.8	23.5	0.9	0.8	90.9	0.7	0.1	55.3	0.6	0.2
TGRFG	31.0	1.3	0.8	24.4	1.1	0.8	90.1	0.9	0.1	64.9	0.8	0.1
VGRFG	2.6	10.6	6.9	1.9	8.0	6.8	1.4	6.3	6.8	1.1	5.1	6.9
NPNPC	15.8	0.3	1.3	12.6	0.3	1.2	2.7	0.2	2.0	1.8	0.2	2.0
NPNPI	44.8	2.0	1.1	39.0	1.5	1.1	11.1	1.2	1.7	33.9	1.0	0.9
NPNPM	13.4	0.9	1.3	7.9	0.7	1.5	90.5	0.5	0.2	3.4	0.4	1.8
NPNPP	2.0	0.3	2.6	2.6	0.3	1.8	1.3	0.2	2.4	6.0	0.2	1.0
NPNPV	10.8	0.8	1.4	5.1	0.6	1.8	2.8	0.5	2.2	8.1	0.4	1.2
NPNPY	14.0	0.9	1.3	7.0	0.7	1.6	90.7	0.5	0.2	2.0	0.4	2.5
CPNPT	2.0	0.5	2.0	6.1	0.3	1.1	2.0	0.2	1.7	9.1	0.2	0.7
IPNPT	39.7	2.1	0.9	28.2	1.7	0.9	27.0	1.2	0.8	105.2	1.0	0.0
LPNPT	44.1	2.5	1.0	39.5	1.9	0.9	14.3	1.4	1.3	26.0	1.1	0.8
MPNPT	36.5	1.3	0.8	27.9	1.0	0.8	97.8	0.8	0.1	59.3	0.6	0.2
QPNPT	1.5	0.4	4.6	1.1	0.3	5.1	0.9	0.2	5.4	0.7	0.2	5.5
VPNPT	22.2	0.9	0.8	78.6	0.7	0.1	41.5	0.5	0.3	7.1	0.4	1.0
YPNPT	38.1	1.4	0.8	28.5	1.0	0.8	96.3	0.8	0.1	45.5	0.6	0.3

In addition, the *k*-values calculated by the parameters $k_{HA}$ , $k_{A}$ , $pK_{a}$ , and different pH values as described in Eq. 1 were fitted with 1/T , φ, and $P_{m}^{N}$ to examine the consistency of the parameter results in the S-curve according to Eqs 4, 5, and Eq. 7 (Supplementary Tables S1, S2). For the independent variable 1/T , we obtained 1,596 correlation coefficients (R^2^), and 73.1% of the R^2^ values were greater than 0.9. For the independent variable of φ or $P_{m}^{N}$ , 1197 R^2^ values were obtained, and 95.4% of the R^2^ values were greater than 0.9 for the independent variable. The results suggested that the parameters conformed to the relationship described in Eqs 4, 5, and Eq. 7 and further indicated the applicability of Eq. 1.

### 4.2 Linear relationships between $k_{HA}$ , $k_{A}$ , and $pK_{a}$ with respect to 1/T

**FIGURE 2:**
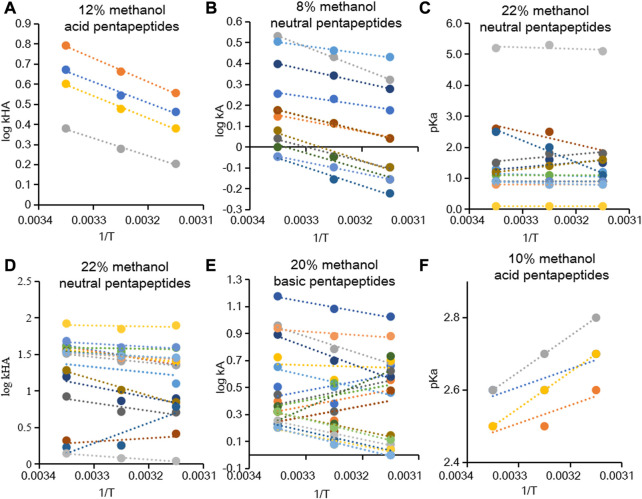
Dependence of $\log k_{HA}$ , $\log k_{A}$ , and $pK_{a}$ of acid pentapeptides in 12% methanol **(A)**, neutral pentapeptides in 8% methanol **(B)**, neutral pentapeptides in 22% methanol **(C)**, neutral pentapeptides in 22% methanol **(D)**, basic pentapeptides in 20% methanol **(E)**, and acid pentapeptides in 10% methanol **(F)** with respect to 1/T .

The temperature of the column could alter the density of the mobile phase, solute diffusion coefficients, and solute–stationary phase interactions and then affect the chromatographic retention (Nagase et al., 2021). The retention of solutes generally decreased as the column temperature was increased due to the accelerated molecular movement in RP-HPLC (Caltabiano et al., 2018; Idroes et al., 2020). Moreover, the $k_{HA}$ , $k_{A}$ , and $pK_{a}$ of solutes were theoretically correlated with the Van’t Hoff equation since they similarly represented an equilibrium state of ionization. Therefore, the relationships between $\log k_{HA},\log k_{A}$ , and $pK_{a}$ with respect to 1/T of each compound under different mobile phase compositions were characterized, 684 R^2^ values (Supplementary Table S3), and 60-line charts of each parameter with respect to 1/T were obtained, and some representative graphs are shown in Figure 2. In this study, the plots of $\log k_{HA},\log k_{A}$ , and $pK_{a}$ with respect to 1/T showed linear relationships consistent with Eqs 4 and 5 for most pentapeptides, indicating that the effect of the temperature on $∆H^{0}$ , $∆S^{0}$ , and log⁡Φ could be commonly disregarded. The linear correlations of acid pentapeptides for the plots of $\log k_{HA}$ vs. 1/T were the most evident, with each R^2^ greater than 0.92 under different mobile phase compositions. The neutral pentapeptides displayed the most apparent linear correlation between $\log k_{A}$ and 1/T , with 63% of the R^2^ > 0.9 and 97.5% of the R^2^ > 0.75. The linear correlations of all pentapeptides between $\log k_{HA},\log k_{A}$ with respect to 1/T (43.1% of the R^2^ > 0.9 and 56.1% of the R^2^ > 0.9) were better than $pK_{a}$ vs. 1/T (18.4% of the R^2^ > 0.9), especially for the basic pentapeptides. Furthermore, the correlations from the same group of compounds in different methanol concentrations were quite different due to the influence of the mobile phase compositions on the retention of the pentapeptides, and the linear correlations of the acid pentapeptides were slightly better than those of the neutral and basic pentapeptides.

The linear relationship between $k_{HA}$ , $k_{A}$ , and $pK_{a}$ with respect to 1/T depended on the $∆H^{0}$ , $∆S^{0}$ , and Φ under different chromatographic conditions. However, the contributions of enthalpy and entropy to the retention process varied with the change in temperature. Enthalpy-driven effects substituted entropy-driven effects at a higher column temperature and affected the linearity of the Van’t Hoff equation (Tanase et al., 2019). Moreover, the Φ value was affected by mobile phase composition and continuously increased with an increase in temperature in the range of 5–50°C (Flieger et al., 2019). A previous study reported the linear dependence of acid compounds and the non-linear behavior of basic compounds with the Van’t Hoff equation (Galaon and David, 2011; Yılmaz Ortak and Cubuk Demiralay, 2019). Except for the compounds with unique structures, other compounds potentially participated in multiple interactions with the stationary phase, likely causing the deviations from the Van’t Hoff equation. In summary, the $\log k_{HA}$ and $\log k_{A}$ of acidic and neutral pentapeptides followed the description of Van’t Hoff equation and had apparent linear relationships with 1/T . Therefore, in this study, only a limited prediction capacity of $pK_{a}$ was observed with a minimal deviation contribution to the overall retention due to the assumption of linearity.

### 4.3 Linear relationships between $k_{HA}$ , $k_{A}$ , and $pK_{a}$ with respect to X

**FIGURE 3:**
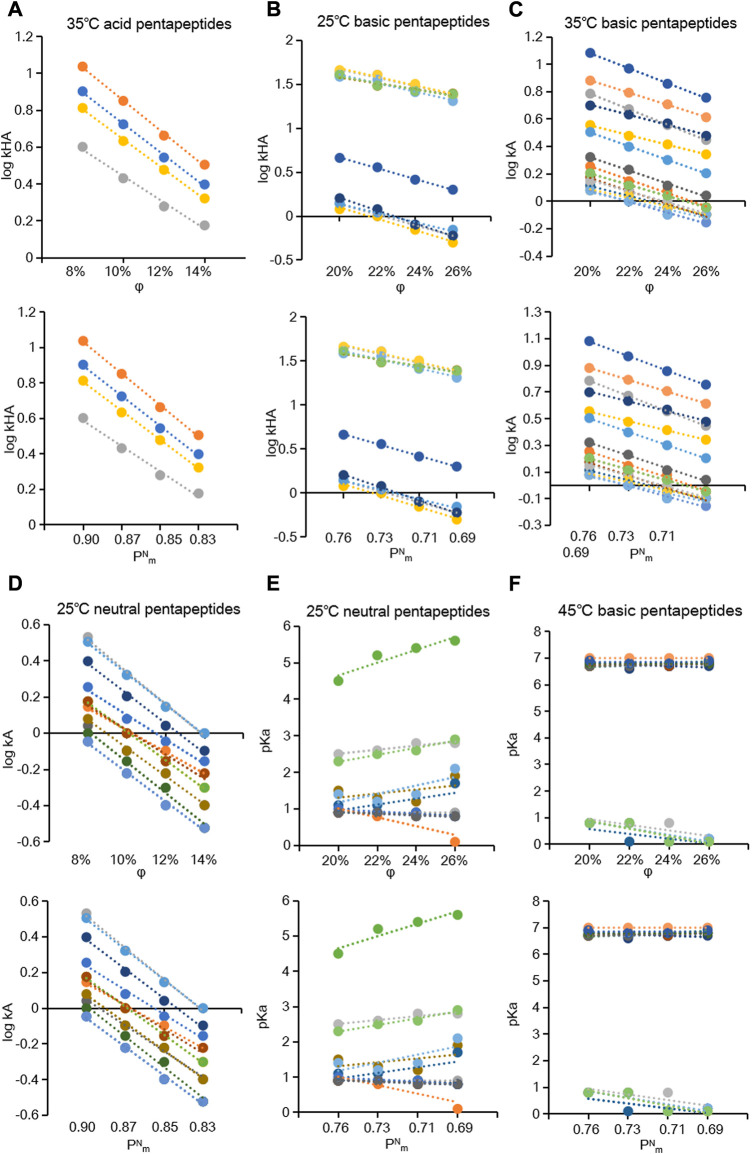
Plots of the $\log k_{HA}$ , $\log k_{A}$ , and $pK_{a}$ of 8%–14% methanol acid pentapeptides at 35°C **(A)**, 20%–26% methanol basic pentapeptides at 25°C **(B)**, 20%–26% methanol basic pentapeptides at 35°C **(C)**, 8%–14% methanol neutral pentapeptides at 25°C **(D)**, and 20%–26% methanol neutral pentapeptides at 25°C **(E)**, 20%–26% methanol basic pentapeptides at 45°C **(F)** as a function of φ or $P_{m}^{N}$ .

An appropriate composition of the mobile phase is beneficial for chromatographic separation and improving the chromatographic peak profile and efficiencies (Guo et al., 2018; Attwa et al., 2023). Adjusting the proportion of organic modifiers in the mobile phase is the most frequently used approach to achieve the separation of a series of compounds (Hong et al., 2020; Oney-Montalvo et al., 2022). Furthermore, the methanol volume fraction φ and polarity parameter $P_{m}^{N}$ are commonly used to characterize the composition of the mobile phase. Hence, in this study, the two factors were considered simultaneously. We examined the influence of the composition of the mobile phase on $\log k_{HA}$ , $\log k_{A}$ , and $pK_{a}$ at a constant temperature. 90-line charts of five groups and 1026 R^2^ values (Supplementary Table S4) were obtained, and some representative charts were selected as shown in Figure 3. Identical R^2^ values were acquired depending on the descriptor (φ or $P_{m}^{N}$ ) of the composition of the mobile phase at the same temperature; these results indicated that the relationship between φ and $P_{m}^{N}$ could be regarded as linear in a narrow range of mobile phase compositions. Moreover, all plots of $\log k_{HA}$ , $\log k_{A}$ , and $pK_{a}$ with respect to X exhibited linear correlations for the five groups of pentapeptides, which was consistent with Eq. 7; Eq. 10; Eqs 11, 12. More importantly, the linear model was highly suitable for describing the relationship of acid pentapeptides between $\log k_{HA}$ and $\log k_{A}$ with respect to X , with each R^2^ greater than 0.98 or 0.9. For neutral or basic pentapeptides, the linear fitting results of $\log k_{A}$ and X (90% of the R^2^ > 0.9 and 79.7% of the R^2^ > 0.9, respectively) surpassed those of $\log k_{HA}$ and $pK_{a}$ . Due to the influence of temperature on chromatographic retention, there were differences in the correlations of the same group of compounds at different temperatures, and the R^2^ values were generally lower with increasing temperature. These results indicated that the two functional relationships of Eqs 11, 12 were appropriate for studying the change in the chromatographic retention of the 57 pentapeptides in the range of mobile phase compositions, and it was very difficult to compare the superiority of the independent variables of φ and $P_{m}^{N}$ based on the current results. Specifically, $\log k_{HA}$ , $\log k_{A}$ , and $pK_{a}$ had evident linear relationships with φ or $P_{m}^{N}$ , and there was basically no difference regardless of whether φ or $P_{m}^{N}$ was used as the descriptor of the composition of the mobile phase.

The coefficient $\log k_{W}$ of the Soczewiński–Wachtmeister equation is related to not only the length of the alkyl chain in the molecule but also the latitude of the mobile phase composition (Żesławska et al., 2022). Generally, a wider range of methanol concentrations correlates with more accurate coefficients. A previous study reported the linear relationship of k and φ at 60%–80% methanol (Elmansi et al., 2019). However, in our study, the pentapeptides analyzed generally had short carbon chains with high polarity and showed low retention in RP-HPLC. No retention occurred at a high proportion of methanol, and a broad chromatographic peak with long tailing was observed at a low proportion of methanol. Hence, we used a narrow range of methanol concentrations to produce symmetrical and sharp peaks, which was crucial for accurately recording the retention time. Furthermore, we classified the pentapeptides into two groups to investigate their linear dependence at different ranges of methanol concentrations. The linear relationships need to be verified at a wider range of methanol concentrations in future studies.

### 4.4 Six-parameter model of pH and T for the prediction of the chromatographic retention factor

We combined the two variables of temperature and pH into a six-parameter model (as shown in Eq. 6) to explore the combined effect of temperature and pH on chromatographic retention. The fitting parameters a, b, c, d, e, and f (Table 5) of the 57 pentapeptides under the four mobile phase compositions were calculated by an established six-parameter model with pH and T as independent variables and k as the dependent variable to predict the k-value according to different pH and T values. All fitting parameters varied with the change in the mobile phase composition except pH and T. Moreover, a higher proportion of methanol in the mobile phase correlated to a smaller parameter of the acid pentapeptides. Only parameter c displayed an inversely proportional relationship with the proportion of methanol for neutral and basic pentapeptides, and there were no clear trends for other parameters in most cases.

**TABLE 5:** Parameter results of pH and T under different mobile phase compositions.

φ	8%						10%					
parameter	a	b	c	d	e	f	a	b	c	d	e	f
APNPT	1.541	4.630	0.093	4.208	0.942	0.810	1.398	5.076	0.009	1.912	0.900	0.000
DPNPT	0.583	11.223	-0.279	5.938	2.600	0.000	0.398	10.949	-0.370	4.007	2.787	-5.061
EPNPT	0.662	13.270	-0.279	5.938	2.645	-5.870	0.496	11.822	-0.301	0.000	2.687	-5.061
GPNPT	1.796	-13.863	-0.068	5.561	0.408	26.113	0.879	11.300	-0.208	5.275	1.400	-7.692
HPNPT	1.371	11.615	0.083	11.401	0.900	0.000	1.355	6.135	-0.060	9.655	0.713	5.061
KPNPT	1.319	6.110	-0.026	0.371	0.900	0.000	1.346	1.213	-0.155	0.000	0.655	5.870
NPNPT	1.548	-3.969	-0.112	7.320	0.755	13.563	0.656	21.115	-0.316	8.210	1.689	-19.433
PPNPT	1.624	7.983	0.343	4.080	1.000	0.000	1.496	6.173	0.172	3.740	0.942	0.810
RPNPT	1.559	7.418	0.044	5.800	0.813	5.061	1.312	10.398	-0.141	6.354	0.900	0.000
SPNPT	0.878	5.871	-0.112	7.320	1.613	5.061	0.731	13.824	-0.208	5.275	1.632	-10.931
TPNPT	1.711	2.069	0.143	6.481	0.855	5.870	0.682	23.823	0.019	4.791	1.979	-23.482
NPNPA	1.435	-4.867	-0.112	7.320	0.897	14.372	0.694	13.391	-0.208	5.275	1.689	-11.741
NPNPD	0.316	10.435	-0.538	5.689	2.558	-0.810	0.102	11.117	-0.954	10.337	3.032	-10.931
NPNPE	0.453	12.951	-0.416	4.648	2.558	-0.810	0.233	13.655	-1.033	16.246	2.932	-10.931
NPNPG	1.274	4.498	-0.267	7.705	0.800	0.000	1.432	14.429	-0.403	7.334	0.489	-11.741
NPNPH	1.211	7.074	-0.322	9.923	0.800	0.000	1.569	10.159	-0.551	11.342	0.287	-5.061
NPNPK	2.858	-33.993	-0.364	6.792	-0.913	41.093	0.535	33.019	-0.538	5.689	1.382	-30.162
NPNPN	1.457	13.071	-0.438	9.795	0.432	-10.931	-1.684	79.934	-0.597	9.553	4.605	-105.668
NPNPQ	0.737	9.634	-0.316	8.210	1.426	-5.263	-1.076	79.689	-0.467	7.937	3.895	-102.024
NPNPR	1.224	10.416	-0.188	8.243	0.900	0.000	2.505	-29.224	-0.379	9.093	-0.653	41.296
NPNPS	1.405	-9.866	-0.279	5.938	0.479	22.672	-0.950	77.379	-0.416	4.648	3.521	-91.903

φ	12%						14%					
parameter	a	b	c	d	e	f	a	b	c	d	e	f
APNPT	1.590	-12.004	-0.171	3.003	0.437	21.862	3.646	-71.281	-0.319	3.930	-2.318	100.607
DPNPT	0.206	11.649	-0.844	13.024	3.276	-16.802	0.062	11.198	-1.135	17.672	3.318	-15.992
EPNPT	0.276	12.998	-0.523	0.000	2.932	-10.931	0.196	10.253	-0.699	0.000	2.787	-5.061
GPNPT	3.746	-71.073	-0.364	6.792	-2.216	93.927	-1.877	91.647	-0.416	4.648	5.124	-121.660
HPNPT	1.122	9.490	-0.273	10.559	0.800	0.000	2.428	-25.956	-0.471	12.012	-0.726	36.032
KPNPT	1.262	13.996	-0.416	4.648	0.634	-9.919	1.880	-15.067	-0.398	0.000	-0.239	23.279
NPNPT	3.240	-52.069	-0.467	7.937	-1.450	65.385	-1.408	80.165	-0.538	5.689	3.887	-89.676
PPNPT	2.023	-19.052	0.067	1.889	-0.011	34.413	1.340	2.327	0.000	0.000	0.655	5.870
RPNPT	1.136	9.618	-0.346	8.624	0.900	0.000	2.758	-33.394	-0.365	4.571	-1.058	46.964
SPNPT	2.806	-53.225	-0.319	3.930	-0.826	66.802	-1.696	88.564	-0.482	4.904	4.576	-109.109
TPNPT	1.333	5.730	-0.145	4.746	0.900	0.000	0.688	7.578	-0.239	3.404	1.516	-1.619
NPNPA	3.766	-71.731	-0.280	3.388	-2.216	93.927	-1.933	92.633	-0.416	4.648	5.426	-128.340
NPNPD	-0.008	9.733	-1.261	15.234	2.887	-5.061	-0.084	8.095	-1.000	0.000	2.539	7.490
NPNPE	0.115	12.239	-0.954	10.337	2.932	-10.931	-0.007	11.052	-1.436	17.672	2.887	-5.061
NPNPG	-1.390	76.360	-0.653	10.337	4.142	-99.190	1.636	-33.743	-0.704	7.334	-1.026	82.186
NPNPH	2.265	-11.807	-0.653	10.337	-0.576	16.802	1.733	5.741	-0.704	7.334	-0.200	0.000
NPNPK	2.798	-36.462	-0.704	7.334	-1.113	41.093	1.735	1.909	-0.954	10.337	-0.100	0.000
NPNPN	0.306	17.503	-0.704	7.334	1.434	-17.611	-0.798	36.650	-0.954	10.337	3.345	-59.717
NPNPQ	1.268	-25.543	-0.538	5.689	-0.100	61.538	1.561	-33.940	-0.523	0.000	-1.461	99.798
NPNPR	0.772	32.043	-0.513	8.578	1.034	-25.304	0.998	21.283	-0.538	5.689	0.587	-12.753
NPNPS	0.794	-6.502	-0.631	6.323	0.497	37.449	1.581	-33.647	-0.523	0.000	-1.316	93.927

φ	20%						22%					
parameter	a	b	c	d	e	f	a	b	c	d	e	f
HGRFA	-1.307	80.053	0.727	-6.964	11.737	-293.522	-1.440	81.555	0.650	-8.090	11.882	-299.393
HGRFD	1.110	16.525	-0.460	13.272	0.900	0.000	1.037	14.904	-0.438	9.795	0.900	0.000
HGRFE	1.164	14.650	-0.405	11.054	0.900	0.000	1.087	12.992	-0.438	9.795	0.900	0.000
HGRFG	-1.387	80.417	0.611	-6.859	11.737	-293.522	-1.370	76.981	0.513	-6.912	11.866	-297.773
HGRFH	-0.477	16.082	0.356	15.012	6.700	0.000	-0.471	12.012	0.277	13.711	6.700	0.000
HGRFK	-0.529	15.875	0.580	2.641	6.787	-5.061	-0.619	16.019	0.534	1.098	6.974	-10.121
HGRFN	-0.501	16.418	0.211	10.855	6.700	0.000	-0.678	18.449	0.137	9.672	6.700	0.000
HGRFQ	-2.370	94.907	0.733	-9.534	15.239	-346.356	-0.606	18.287	0.138	10.622	6.555	5.870
HGRFR	-0.453	16.552	0.197	17.369	7.103	-14.372	-0.704	20.253	0.453	7.560	6.600	0.000
HGRFS	-1.438	80.133	0.527	-6.656	11.679	-292.713	-1.526	79.036	0.494	-8.797	11.866	-297.773
HGRFT	-1.428	84.350	0.704	-7.900	11.737	-293.522	-1.450	81.131	0.607	-8.005	11.737	-293.522
AGRFG	1.292	7.659	-0.075	9.868	0.713	5.061	1.297	4.986	-0.190	10.511	0.655	5.870
DGRFG	1.194	10.893	-0.316	12.318	0.900	0.000	1.162	8.662	-0.431	12.858	0.900	0.000
EGRFG	1.271	10.561	-0.412	14.907	0.900	0.000	1.203	9.112	-0.431	12.858	0.900	0.000
GGRFG	1.261	6.571	-0.190	10.511	0.655	5.870	2.317	-23.532	-0.273	10.559	-0.508	35.425
KGRFG	-1.615	85.720	0.978	-17.695	11.924	-298.583	-0.626	19.806	0.633	0.462	7.118	-15.992
NGRFG	1.347	6.131	-0.250	11.342	0.655	5.870	1.134	10.339	-0.250	9.005	0.800	0.000
PGRFG	-0.188	16.246	0.796	3.446	7.000	0.000	-0.300	16.354	0.712	3.085	7.000	0.000
QGRFG	1.380	6.955	-0.137	9.795	0.655	5.870	1.202	10.227	-0.209	9.696	0.800	0.000
RGRFG	1.417	6.305	-0.172	9.247	0.568	10.931	1.220	9.540	-0.226	8.669	0.713	5.061
SGRFG	1.319	6.453	-0.250	11.342	0.713	5.061	1.158	9.970	-0.250	9.005	0.800	0.000
TGRFG	1.325	6.994	-0.135	11.497	0.713	5.061	1.296	5.117	-0.190	10.511	0.655	5.870
VGRFG	0.131	13.501	0.840	8.386	6.611	11.741	-0.044	15.190	0.729	8.049	6.568	10.931
NPNPC	1.237	4.919	-0.631	6.323	0.779	14.980	1.119	5.598	-0.704	7.334	0.824	9.109
NPNPI	1.279	8.689	0.171	6.198	1.505	-5.668	1.535	1.541	0.041	6.693	1.100	0.000
NPNPM	1.225	-3.495	-0.200	6.964	0.866	17.611	0.450	17.625	-0.346	8.624	1.992	-18.421
NPNPP	0.103	8.982	-0.704	7.334	2.745	-5.870	0.457	-4.152	-0.523	0.000	1.047	41.296
NPNPV	0.776	11.470	-0.208	5.275	1.632	-10.931	0.399	12.654	-0.364	6.792	2.234	-17.611
NPNPY	0.833	9.218	-0.316	12.318	1.445	1.822	0.308	24.366	-0.376	10.640	2.063	-21.862
CPNPT	0.498	0.293	-0.301	0.000	1.045	24.899	1.282	-28.282	-0.631	6.323	-0.405	74.899
IPNPT	1.438	6.855	0.246	3.976	0.900	0.000	1.318	5.651	0.175	2.635	0.900	0.000
LPNPT	1.522	6.581	0.321	4.126	0.942	0.810	1.472	5.031	0.196	4.355	0.900	0.000
MPNPT	1.519	2.092	-0.019	6.336	0.655	5.870	1.325	4.991	-0.141	6.354	0.800	0.000
QPNPT	0.011	7.108	-0.538	5.689	4.861	-7.490	-0.088	5.842	-0.625	1.426	5.071	4.251
VPNPT	1.220	5.038	-0.085	2.316	0.800	0.000	1.829	1.939	-0.205	2.935	0.100	0.000
YPNPT	1.377	7.693	-0.103	11.773	0.713	5.061	1.361	4.168	-0.268	12.606	0.655	5.870

φ	24%						26%					
parameter	a	b	c	d	e	f	a	b	c	d	e	f
HGRFA	-0.390	14.601	0.233	10.174	6.555	5.870	-0.404	12.487	0.138	10.622	6.655	5.870
HGRFD	0.677	20.511	-0.467	7.937	1.274	-10.121	0.647	16.824	-0.597	9.553	1.203	-6.680
HGRFE	0.733	18.486	-0.513	8.578	1.274	-10.121	0.573	19.975	-0.653	10.337	1.347	-12.551
HGRFG	3.354	-78.572	-0.759	32.016	-7.942	353.036	0.585	-0.816	-0.175	13.408	2.863	55.061
HGRFH	-0.551	11.342	0.153	14.344	6.555	5.870	-0.597	9.553	0.086	13.428	6.742	0.810
HGRFK	-0.648	12.842	0.411	3.099	6.974	-10.121	-0.716	11.227	0.339	3.521	6.974	-10.121
HGRFN	-0.732	16.246	0.048	9.706	6.700	0.000	-0.927	19.806	-0.024	9.252	6.742	0.810
HGRFQ	-0.652	16.489	0.058	10.166	6.800	0.000	0.404	0.364	-0.259	13.244	3.108	49.190
HGRFR	-0.732	16.246	0.397	7.520	6.787	-5.061	-0.830	15.875	0.310	8.147	6.787	-5.061
HGRFS	0.597	-0.981	-0.155	13.145	2.821	54.251	0.423	0.099	-0.201	12.483	3.066	48.381
HGRFT	-1.484	79.378	0.502	-7.767	12.011	-303.644	0.314	2.631	-0.087	12.775	3.268	49.393
AGRFG	-1.464	77.778	0.415	-8.063	12.197	-308.704	-1.264	76.742	0.336	-7.900	11.879	-308.097
DGRFG	2.464	-27.677	-0.504	12.481	-0.508	35.425	1.153	10.196	-0.551	11.342	0.768	-4.453
EGRFG	1.205	5.522	-0.652	16.489	0.900	0.000	1.758	-4.872	-0.732	16.246	0.208	10.729
GGRFG	2.572	-26.768	-0.322	9.923	-0.826	36.032	1.278	16.812	-0.405	11.054	0.432	-10.931
KGRFG	-0.833	21.393	0.567	1.199	6.974	-10.121	-0.881	19.164	0.530	1.302	6.974	-10.121
NGRFG	2.374	-26.833	-0.405	11.054	-0.508	35.425	1.361	17.318	-0.438	9.795	0.432	-10.931
PGRFG	-2.162	88.276	1.280	-23.430	15.974	-363.968	-0.454	14.528	0.520	4.015	6.942	0.810
QGRFG	1.120	9.045	-0.200	6.964	0.800	0.000	2.595	-28.593	-0.237	5.689	-0.913	41.093
RGRFG	2.363	-23.727	-0.322	9.923	-0.508	35.425	2.576	-28.144	-0.346	8.624	-0.913	41.093
SGRFG	2.399	-27.198	-0.405	11.054	-0.508	35.425	2.452	-26.575	-0.404	9.326	-0.726	36.032
TGRFG	2.375	-26.256	-0.273	10.559	-0.508	35.425	2.541	-26.471	-0.322	9.923	-0.826	36.032
VGRFG	-0.153	14.528	0.630	7.760	6.568	10.931	-0.240	13.924	0.546	7.316	6.755	5.870
NPNPC	-0.361	37.458	-0.954	10.337	2.868	-42.915	0.037	13.681	-0.699	0.000	1.913	-2.632
NPNPI	0.193	35.296	-0.063	6.792	2.800	-46.154	2.098	-28.002	-0.091	4.313	-0.026	43.725
NPNPM	3.664	-68.663	-0.467	7.937	-2.029	88.866	-1.505	83.357	-0.597	9.553	4.261	-99.798
NPNPP	0.060	3.145	-0.699	0.000	1.763	24.291	1.405	-35.654	-0.873	2.437	-0.911	95.951
NPNPV	-0.933	78.877	-0.370	4.007	4.024	-106.275	1.313	-24.013	-0.538	5.689	0.187	56.478
NPNPY	2.604	-45.051	-0.586	13.802	-0.668	58.300	-0.024	16.131	-0.694	13.483	2.847	-20.243
CPNPT	0.443	-9.186	-0.852	8.912	0.745	47.976	1.792	-48.534	-0.699	0.000	-1.558	116.194
IPNPT	1.373	3.069	0.021	3.388	0.742	0.810	2.585	-33.942	-0.036	2.095	-0.695	40.486
LPNPT	0.774	19.991	0.058	4.480	1.647	-20.243	1.330	3.685	-0.022	3.672	0.800	0.000
MPNPT	2.458	-29.520	-0.208	5.275	-0.508	35.425	1.637	7.098	-0.364	6.792	0.287	-5.061
QPNPT	-0.145	4.746	-0.699	0.000	4.821	8.097	-0.279	5.938	-0.263	-17.672	4.718	14.777
VPNPT	1.505	4.726	-0.370	4.007	0.387	-5.061	1.720	-31.716	-0.482	4.904	-0.476	55.263
YPNPT	2.342	-24.773	-0.296	9.553	-0.508	35.425	2.281	-21.702	-0.438	9.795	-0.539	30.972

**FIGURE 4:**
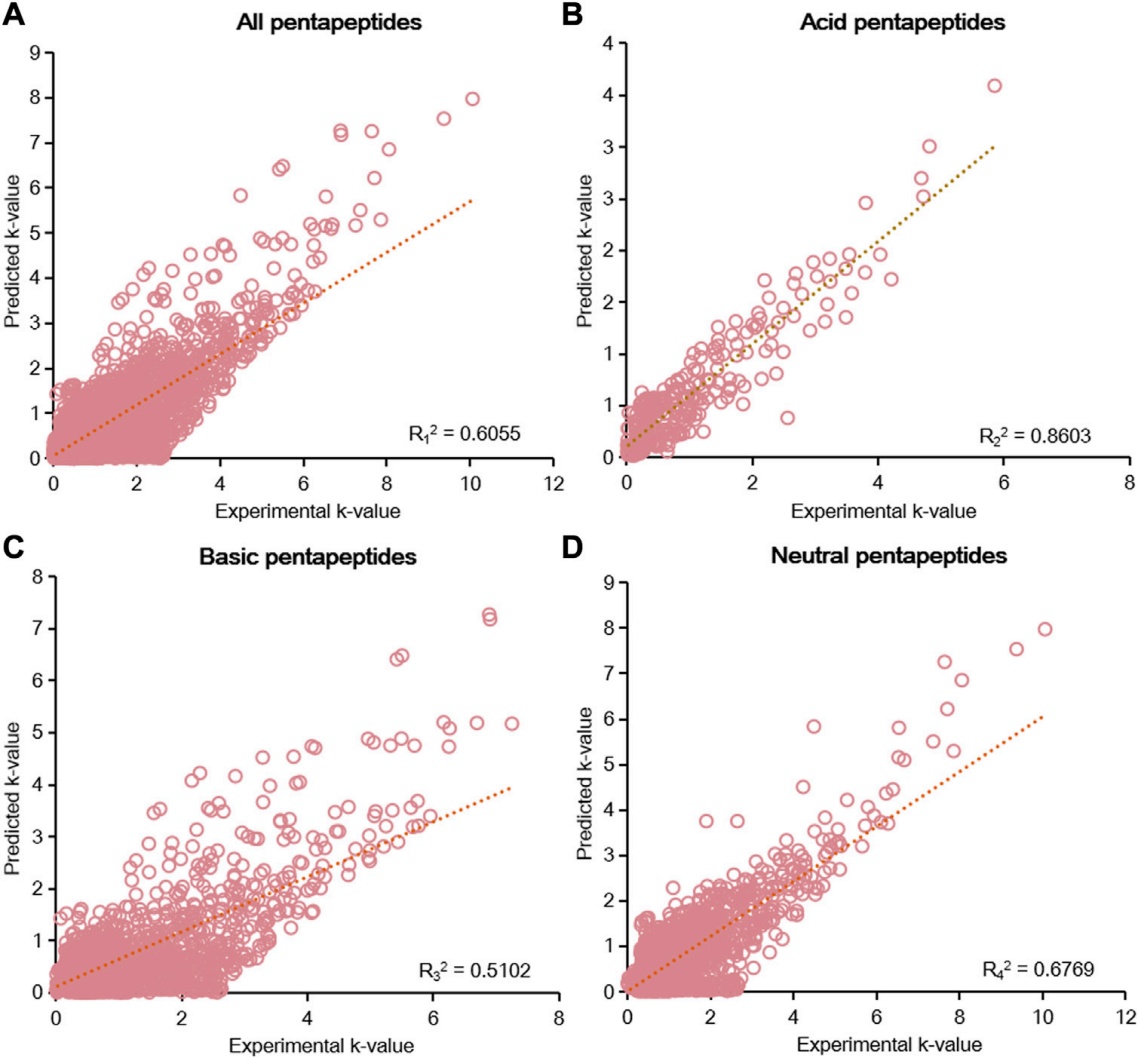
Linear fitting results of the predicted k-value and experimental k-value for all pentapeptides **(A)**, acid pentapeptides **(B)**, basic pentapeptides **(C)**, and neutral pentapeptides **(D)** in the six-parameter model of T and pH.

According to the results of the six-parameter model, linear fitting of the experimental k-value and predicted k-value was conducted to assess the prediction capability of chromatographic retention. The R^2^ calculated by linear fitting was used as an evaluation criterion. A random error was present for all data, but the residuals were symmetrically distributed around the axis of y = 0 (Supplementary Figure S3). We then fitted the data from five groups of pentapeptides, and the R_1_^2^ value was just 0.6055, showing the unsatisfactory capacity to predict the chromatographic retention of the studied pentapeptides (Figure 4A). Further classifying the data according to their acid–base properties and fittings, the R_2_^2^ value was 0.8603 for the acid pentapeptides (Figure 4B), while both the R_3_^2^ and R_4_^2^ values were lower than 0.7 for the basic and neutral pentapeptides (Figures 4C, D). The results indicated that the six-parameter model had a certain prediction capability for the chromatographic retentions for the acid pentapeptides but was unable to characterize the chromatographic retentions for the basic or neutral pentapeptides. In addition, the R^2^ values fitted by the experimental k-value and predicted k-value under different chromatographic conditions in the six-parameter model of T and pH are shown in Supplementary Table S5. The R^2^ decreased with the increase in column temperature or the methanol volume fraction, indicating that this model was suitable for compounds with higher chromatographic retention.

Internal validation is a commonly used method for evaluating models free of experimental and environmental conditions’ limitations (Luo et al., 2020; Vasconcelos et al., 2023). In this study, we used 10-fold cross validation to conduct internal validation. The root mean squared error (RMSE) obtained from 10-fold cross validation was used to evaluate the prediction capability of the models in this study. The average RMSE from the 10 test sets was used to minimize the biased prediction results. The residuals of all pentapeptides and acid pentapeptides were randomly distributed around the y = 0 axis (Supplementary Figure S5). Moreover, the average RMSE of all pentapeptides and acid pentapeptides was 0.48 and 0.20 in the 10 tests (Supplementary Table S7), respectively, indicating that the six-parameter model had both random error and certain prediction capability.

### 4.5 The six-parameter model of pH and mobile phase compositions for the prediction of the chromatographic retention factor

We considered the combined influence of the mobile phase composition and pH on chromatographic retention by substituting pH and X into Eq. 13 and obtained the fitting parameters A, B, C, D, E, and F of the 57 pentapeptides at three temperatures (Tables 6, 7). X represented φ or $P_{m}^{N}$ in the solvent model and was used as the variable to describe the change in the mobile phase. Hence, a six-parameter model was constructed with pH and X as the independent variables and k as the dependent variable, and this model was applied for the prediction of the k-value based on different pH and X values. All fitting parameters varied with the change in temperature expect pH and φ or $P_{m}^{N}$ . More importantly, parameter A of acid pentapeptides and parameter C of neutral pentapeptides increased with a decrease in column temperature when φ was the descriptor for the mobile phase composition, while there was no distinct tendency from the other parameters. The six parameters of the 57 pentapeptides deviated from a positive or negative correlation when $P_{m}^{N}$ was the descriptor for the mobile phase composition.

**TABLE 6:** Parameter results of fitting with φ and pH at different temperatures.

T	25°C						35°C						45°C					
Parameter	A	B	C	D	E	F	A	B	C	D	E	F	A	B	C	D	E	F
APNPT	3.30	-18.41	0.78	-6.78	-0.43	15.50	1.35	2.14	0.84	-7.66	1.96	-11.00	1.32	2.49	0.71	-6.66	2.07	-12.00
DPNPT	1.72	-8.73	0.38	-5.68	2.46	1.50	1.57	-8.48	0.77	-10.54	2.26	4.00	1.53	-8.83	0.60	-9.27	2.03	7.00
EPNPT	1.96	-9.76	0.81	-10.91	2.17	3.00	1.75	-8.93	0.71	-10.14	2.33	2.00	1.67	-9.12	0.60	-9.27	2.24	3.50
GPNPT	0.55	6.97	0.62	-6.00	2.97	-17.00	2.27	-9.16	0.65	-6.77	0.36	6.50	2.73	-13.58	0.49	-5.76	-0.28	13.00
HPNPT	2.50	-8.43	1.22	-8.85	1.07	-2.00	1.38	2.02	1.13	-8.87	2.05	-12.50	1.48	0.70	1.05	-9.12	1.83	-10.50
KPNPT	1.99	-4.45	0.50	-6.30	1.14	-4.00	0.82	6.51	0.44	-6.01	2.28	-15.50	1.59	-1.19	0.52	-6.70	1.45	-7.50
NPNPT	0.92	5.12	0.80	-7.93	2.55	-15.00	2.08	-7.23	0.80	-8.56	0.83	2.00	2.45	-10.61	0.60	-7.32	0.53	4.50
PPNPT	2.72	-10.27	1.17	-8.46	1.02	0.50	2.41	-7.22	1.07	-7.76	1.23	-3.00	2.33	-6.59	0.99	-7.18	1.34	-4.00
RPNPT	2.60	-8.97	0.84	-7.07	1.09	-1.50	1.36	2.98	0.87	-8.27	2.21	-13.50	1.49	1.29	0.69	-6.74	2.02	-12.00
SPNPT	0.15	10.71	0.80	-7.93	3.79	-24.00	1.83	-9.03	0.67	-7.11	1.48	2.00	1.58	-4.02	0.62	-7.21	1.52	-2.00
TPNPT	2.85	-12.08	1.04	-8.24	0.66	4.00	2.78	-13.15	1.01	-8.34	0.51	6.50	2.65	-11.76	0.85	-7.30	0.79	3.50
NPNPA	0.57	6.77	0.69	-6.74	2.97	-17.00	1.82	-6.28	0.65	-6.77	0.86	4.00	2.48	-11.63	0.49	-5.76	-0.04	11.50
NPNPD	1.41	-8.47	0.62	-11.37	1.96	6.50	1.16	-7.15	0.29	-9.03	2.65	0.00	1.16	-7.85	0.38	-10.54	2.23	4.50
NPNPE	1.70	-9.08	0.40	-7.78	2.22	3.00	1.46	-8.14	0.62	-11.37	2.46	1.50	1.41	-8.52	0.48	-10.48	2.18	4.50
NPNPG	3.59	-20.35	0.62	-7.21	-1.90	25.00	2.66	-13.50	0.59	-8.04	-0.08	8.00	2.87	-16.23	0.49	-7.27	-0.32	12.00
NPNPH	1.14	6.02	0.70	-7.78	1.85	-15.00	0.92	7.84	0.59	-8.04	1.91	-16.00	0.84	8.30	0.43	-6.87	2.02	-17.00
NPNPK	1.37	2.21	0.43	-6.87	1.56	-11.00	1.78	-0.25	0.52	-8.79	0.92	-7.00	1.90	-1.17	0.40	-7.78	0.84	-6.50
NPNPN	3.86	-22.99	0.59	-8.04	-1.45	17.50	3.06	-20.70	0.60	-9.27	-0.61	16.00	3.36	-24.18	0.40	-7.78	-1.26	23.50
NPNPQ	3.14	-20.77	0.70	-8.57	-1.70	30.00	2.79	-16.15	0.59	-8.04	-0.35	12.50	0.48	1.80	0.32	-6.00	3.02	-14.50
NPNPR	1.26	3.98	0.75	-7.48	2.12	-14.50	0.74	8.61	0.60	-7.32	2.46	-18.50	2.19	-6.04	0.52	-6.70	0.91	-3.50
NPNPS	3.15	-20.61	0.59	-8.04	-1.59	29.00	2.13	-8.76	0.43	-6.87	0.61	1.50	1.49	-7.17	0.35	-6.63	1.48	-0.50
HGRFA	8.07	-31.23	0.00	2.26	-23.75	120.00	7.75	-30.35	-0.05	1.97	-23.20	117.50	1.06	-4.72	1.60	-4.71	6.38	1.50
HGRFD	3.19	-7.12	1.08	-5.06	0.90	0.00	3.37	-8.64	0.59	-3.40	0.26	3.00	2.96	-7.52	0.66	-4.04	0.40	2.50
HGRFE	2.91	-5.96	0.94	-4.49	1.22	-1.50	3.34	-8.49	0.97	-5.25	0.26	3.00	3.01	-7.68	0.66	-4.04	0.40	2.50
HGRFG	7.96	-31.17	-0.09	2.24	-23.29	118.00	0.00	6.93	1.22	-4.85	3.82	-14.00	-0.22	2.18	1.81	-6.48	11.83	-28.50
HGRFH	1.34	-6.00	2.02	-5.35	6.29	2.00	1.18	-5.76	1.92	-5.65	6.70	0.00	0.66	-4.04	1.66	-4.90	6.38	1.50
HGRFK	1.40	-6.48	1.37	-3.31	6.60	0.00	1.03	-5.14	1.26	-3.52	6.60	0.00	0.79	-5.00	1.44	-3.73	6.43	1.50
HGRFN	1.18	-5.21	1.60	-4.79	6.38	1.50	1.03	-5.14	1.50	-5.00	6.70	0.00	1.04	-6.00	1.31	-4.29	6.38	1.50
HGRFQ	6.18	-25.18	0.34	0.41	-15.40	90.00	-5.20	24.36	2.78	-10.84	27.73	-98.50	0.88	-4.91	1.37	-4.49	5.97	3.50
HGRFR	1.66	-7.27	1.61	-3.71	6.60	0.00	1.25	-6.02	1.43	-3.65	6.23	1.50	1.25	-6.87	0.81	-0.99	7.30	-2.50
HGRFS	7.94	-31.43	-0.22	2.50	-22.88	116.00	-0.04	7.37	1.11	-4.72	3.76	-14.50	1.16	-6.01	1.31	-4.18	6.52	1.00
HGRFT	7.15	-25.84	0.24	0.63	-17.89	88.00	2.29	-3.63	1.28	-4.79	1.63	-3.50	1.09	-5.00	1.55	-4.65	6.29	2.00
AGRFG	2.27	-3.43	1.13	-4.07	1.63	-3.50	-0.07	7.21	1.02	-4.11	3.73	-13.50	8.18	-32.80	-0.51	3.04	-24.21	122.00
DGRFG	2.47	-4.25	1.07	-4.49	1.31	-2.00	0.05	6.62	0.94	-4.49	3.55	-12.50	1.97	-2.19	0.78	-4.16	1.30	-2.50
EGRFG	2.66	-4.94	1.07	-4.49	1.22	-1.50	0.23	5.95	0.94	-4.49	3.46	-12.00	1.81	-1.75	0.97	-5.25	1.54	-3.00
GGRFG	0.19	6.05	0.85	-3.17	3.82	-14.00	-0.17	8.03	0.83	-3.60	3.76	-14.50	1.01	3.02	0.67	-3.20	2.37	-9.00
KGRFG	6.55	-26.75	-0.29	3.78	-14.63	86.00	6.30	-26.19	-0.29	3.16	-14.49	85.50	1.36	-7.27	1.09	-1.82	6.80	0.00
NGRFG	0.21	6.21	1.02	-4.11	3.73	-13.50	0.51	4.55	0.88	-4.00	3.04	-10.50	0.05	6.83	0.79	-3.87	3.35	-12.50
PGRFG	0.44	0.69	2.17	-6.43	12.58	-31.00	1.38	-5.28	1.77	-4.44	7.32	-1.50	1.14	-4.95	1.74	-4.30	7.00	0.00
QGRFG	2.62	-4.74	1.12	-4.31	1.17	-1.50	0.52	4.78	0.96	-4.04	3.04	-10.50	0.51	4.39	0.64	-2.87	3.04	-10.50
RGRFG	2.66	-4.87	0.84	-3.24	1.68	-3.50	0.49	4.95	0.70	-3.08	3.55	-12.50	0.08	7.05	0.67	-3.20	3.67	-14.00
SGRFG	2.58	-4.86	1.02	-4.11	1.17	-1.50	0.28	5.51	0.69	-3.13	3.41	-12.00	0.04	6.94	0.79	-3.87	3.35	-12.50
TGRFG	2.32	-3.67	1.13	-4.07	1.63	-3.50	-0.02	6.98	1.02	-4.11	3.73	-13.50	-0.10	7.65	0.83	-3.60	3.67	-14.00
VGRFG	1.89	-6.13	2.29	-5.61	7.37	-1.50	1.67	-5.67	2.17	-5.46	6.90	0.00	1.66	-6.27	2.07	-5.28	6.85	0.00
NPNPC	4.01	-12.81	0.68	-5.14	0.21	5.50	5.59	-19.45	0.66	-5.40	-2.02	14.00	4.77	-17.50	0.20	-3.52	-1.71	14.50
NPNPI	3.98	-10.79	1.52	-5.52	-0.64	8.00	1.89	-2.68	1.43	-5.35	2.60	-5.00	2.50	-4.54	1.29	-5.00	1.20	0.00
NPNPM	-1.34	11.29	1.14	-5.29	5.75	-20.00	1.84	-3.02	1.03	-5.14	1.20	0.00	1.97	-3.64	1.16	-6.01	0.97	1.00
NPNPP	1.75	-6.48	0.66	-5.40	1.41	5.50	1.17	-4.11	1.16	-8.04	4.01	-7.00	-0.90	5.65	0.20	-3.52	6.78	-21.00
NPNPV	2.70	-6.74	1.03	-5.14	-0.91	9.50	2.00	-4.18	1.09	-5.62	0.20	5.00	1.50	-3.18	0.88	-4.91	1.88	-1.00
NPNPY	3.66	-11.36	1.25	-5.40	-1.12	11.50	2.59	-7.82	1.19	-5.68	0.37	6.00	2.70	-7.11	1.16	-6.01	-1.13	11.00
CPNPT	1.63	-6.29	1.04	-6.59	0.39	9.50	3.39	-13.46	1.04	-6.59	-2.56	19.50	-1.13	7.45	1.02	-6.85	5.17	-16.50
IPNPT	2.88	-5.91	1.60	-6.01	1.31	-2.00	2.56	-4.74	1.50	-5.63	1.40	-2.50	0.19	6.25	1.44	-5.59	3.87	-14.00
LPNPT	2.79	-5.09	1.70	-6.10	1.59	-3.00	2.82	-5.48	1.64	-5.92	1.22	-1.50	2.75	-5.65	1.60	-6.01	1.23	-1.00
MPNPT	0.71	3.91	1.32	-5.48	3.41	-12.00	0.72	3.61	1.25	-5.40	3.04	-10.50	0.34	5.89	1.22	-5.52	3.35	-12.50
QPNPT	1.43	-5.73	1.98	-11.37	1.15	17.50	1.18	-4.91	0.41	-4.52	7.14	-10.50	1.25	-5.40	0.66	-5.40	1.70	15.00
VPNPT	5.36	-17.44	1.03	-5.14	-3.16	17.00	2.63	-5.01	1.03	-5.14	0.68	-1.00	3.45	-8.81	1.16	-6.01	-0.37	4.00
YPNPT	2.91	-6.09	1.74	-6.87	1.31	-2.00	0.55	4.39	1.60	-6.72	3.41	-12.00	0.80	3.80	1.34	-6.00	3.03	-11.00

**TABLE 7:** Parameter results of fitting with $P_{m}^{N}$ and pH at different temperatures.

T	25°C						35°C						45°C					
Parameter	A	B	C	D	E	F	A	B	C	D	E	F	A	B	C	D	E	F
APNPT	-12.02	15.42	-5.05	5.90	12.16	-12.62	2.62	-1.20	-5.70	6.60	-6.83	8.79	3.07	-1.71	-4.97	5.73	-7.79	9.91
DPNPT	-5.75	7.54	-4.54	4.98	3.67	-1.21	-5.67	7.31	-8.11	8.95	5.60	-3.36	-6.02	7.63	-7.25	7.92	7.96	-5.98
EPNPT	-6.40	8.45	-8.54	9.45	4.76	-2.62	-5.87	7.70	-7.94	8.73	4.00	-1.68	-6.14	7.90	-7.25	7.92	5.29	-3.08
GPNPT	5.87	-5.28	-4.53	5.20	-10.83	13.83	-4.58	6.78	-5.14	5.85	4.54	-4.02	-8.64	11.45	-4.46	5.00	10.34	-10.65
HPNPT	-4.74	7.32	-6.37	7.67	-0.60	1.68	2.64	-1.20	-6.47	7.68	-8.11	10.19	1.76	-0.23	-6.71	7.84	-6.82	8.69
KPNPT	-1.60	3.59	-4.88	5.44	-2.52	3.74	5.91	-5.06	-4.68	5.17	-10.47	12.80	0.70	0.89	-5.22	5.81	-5.10	6.64
NPNPT	4.79	-3.84	-5.98	6.85	-9.74	12.34	-3.11	5.10	-6.47	7.34	1.21	-0.19	-6.56	9.09	-5.71	6.39	4.33	-3.83
PPNPT	-6.21	9.05	-6.05	7.30	1.64	-0.65	-3.73	6.19	-5.59	6.73	-1.36	2.62	-3.36	5.75	-5.17	6.23	-2.00	3.36
RPNPT	-4.99	7.66	-5.21	6.11	-0.28	1.40	3.46	-2.06	-6.15	7.09	-8.87	11.12	2.10	-0.54	-5.13	5.89	-7.68	9.72
SPNPT	8.87	-8.75	-5.98	6.85	-16.42	20.37	-5.58	7.43	-5.43	6.16	2.67	-1.12	-1.12	2.62	-5.50	6.18	-1.28	2.99
TPNPT	-7.18	10.08	-6.02	7.13	3.68	-2.99	-8.54	11.44	-6.10	7.18	6.14	-5.70	-7.55	10.32	-5.41	6.32	4.00	-3.27
NPNPA	5.71	-5.10	-5.13	5.89	-10.83	13.83	-2.93	4.70	-5.14	5.85	3.23	-2.24	-7.04	9.55	-4.46	5.00	9.04	-9.07
NPNPD	-5.85	7.33	-9.02	9.73	7.43	-5.51	-4.98	6.21	-7.49	7.88	2.81	-0.19	-5.61	6.84	-8.78	9.28	6.35	-4.21
NPNPE	-6.06	7.83	-6.25	6.71	4.65	-2.43	-5.50	7.03	-9.02	9.73	3.67	-1.21	-5.91	7.40	-8.64	9.24	6.14	-4.02
NPNPG	-12.14	15.64	-5.50	6.18	17.13	-18.88	-8.13	10.79	-6.29	6.95	5.96	-5.98	-10.41	13.33	-5.68	6.23	9.38	-9.72
NPNPH	6.67	-5.64	-5.95	6.71	-11.41	13.46	8.07	-7.30	-6.29	6.95	-12.26	14.39	8.31	-7.60	-5.51	6.01	-12.91	15.14
NPNPK	3.59	-2.29	-5.51	6.01	-8.19	9.91	0.76	1.14	-7.04	7.65	-4.20	5.05	0.18	1.84	-6.25	6.71	-3.99	4.77
NPNPN	-15.62	19.65	-6.29	6.95	13.29	-14.86	-15.87	19.31	-7.25	7.92	14.85	-15.89	-18.34	22.07	-6.25	6.71	20.11	-21.78
NPNPQ	-13.03	16.09	-6.56	7.33	21.59	-23.18	-10.40	13.23	-6.29	6.95	9.81	-10.19	1.58	-1.04	-4.83	5.20	-8.49	11.50
NPNPR	4.42	-3.16	-5.62	6.43	-10.03	12.24	7.80	-7.09	-5.71	6.39	-13.04	15.61	-2.16	4.27	-5.22	5.81	-2.94	4.02
NPNPS	-12.86	15.92	-6.29	6.95	20.95	-22.43	-4.26	6.29	-5.51	6.01	0.69	0.09	-5.54	7.23	-5.36	5.78	2.47	-1.21
HGRFA	-18.11	26.30	1.80	-1.76	76.64	-100.75	-17.78	25.68	1.52	-1.55	75.20	-98.79	-2.95	4.05	-2.43	4.07	7.60	-1.21
HGRFD	-2.88	6.12	-3.27	4.40	0.90	0.00	-3.90	7.32	-2.29	2.90	2.71	-2.43	-3.43	6.46	-2.76	3.45	2.53	-2.15
HGRFE	-2.21	5.19	-2.89	3.87	0.00	1.21	-3.81	7.20	-3.45	4.45	2.71	-2.43	-3.52	6.59	-2.76	3.45	2.53	-2.15
HGRFG	-18.26	26.37	1.73	-1.79	75.56	-99.25	5.57	-5.51	-2.91	4.17	-7.77	11.59	2.10	-2.52	-3.80	5.71	-13.97	26.64
HGRFH	-3.80	5.20	-2.57	4.64	7.97	-1.68	-3.76	5.00	-2.90	4.86	6.70	0.00	-2.76	3.45	-2.52	4.24	7.60	-1.21
HGRFK	-4.06	5.49	-1.50	2.92	6.60	0.00	-3.35	4.42	-1.75	3.04	6.60	0.00	-3.44	4.26	-1.76	3.24	7.79	-1.40
HGRFN	-3.27	4.50	-2.51	4.16	7.60	-1.21	-3.35	4.42	-2.76	4.31	6.70	0.00	-4.10	5.20	-2.36	3.71	7.60	-1.21
HGRFQ	-16.33	23.14	0.97	-0.74	66.07	-84.11	14.44	-19.43	-6.25	9.04	-52.52	79.72	-3.33	4.26	-2.45	3.87	8.87	-2.90
HGRFR	-4.51	6.23	-1.60	3.27	6.60	0.00	-3.85	5.15	-1.66	3.12	7.59	-1.40	-4.67	6.01	0.07	0.71	4.90	2.52
HGRFS	-18.49	26.58	1.78	-1.97	74.29	-97.57	6.09	-6.13	-2.92	4.08	-8.42	12.24	-3.96	5.17	-2.25	3.60	7.43	-0.93
HGRFT	-14.17	21.27	0.58	-0.26	53.80	-71.21	-0.88	3.23	-2.81	4.13	-1.27	2.90	-3.14	4.26	-2.42	4.01	7.97	-1.68
AGRFG	-0.72	3.04	-2.35	3.52	-1.27	2.90	5.72	-5.72	-2.48	3.54	-7.41	11.12	-19.34	27.64	1.96	-2.44	77.99	-102.62
DGRFG	-1.18	3.69	-2.76	3.87	-0.37	1.68	5.29	-5.15	-2.89	3.87	-6.69	10.19	0.38	1.50	-2.80	3.62	-1.10	2.52
EGRFG	-1.59	4.32	-2.76	3.87	0.00	1.21	4.88	-4.54	-2.89	3.87	-6.32	9.72	0.22	1.64	-3.45	4.45	-0.91	2.43
GGRFG	5.05	-4.80	-1.88	2.77	-7.77	11.59	6.51	-6.68	-2.24	3.10	-8.42	12.24	4.19	-3.45	-2.08	2.79	-5.91	8.60
KGRFG	-17.26	24.44	3.21	-3.64	63.09	-80.19	-17.11	24.07	2.72	-3.15	62.91	-79.91	-4.81	6.23	-0.47	1.58	6.80	0.00
NGRFG	5.16	-4.87	-2.48	3.54	-7.41	11.12	4.02	-3.41	-2.53	3.44	-5.52	8.50	5.74	-5.70	-2.48	3.29	-7.16	10.56
PGRFG	1.37	-1.07	-3.45	5.73	-15.48	28.97	-3.13	4.56	-2.01	3.82	6.10	1.21	-3.08	4.26	-1.93	3.71	7.00	0.00
QGRFG	-1.36	3.99	-2.55	3.70	-0.19	1.40	4.22	-3.60	-2.46	3.45	-5.52	8.50	3.92	-3.32	-1.82	2.50	-5.52	8.50
RGRFG	-1.43	4.11	-1.93	2.81	-1.35	3.08	4.32	-3.73	-1.90	2.62	-6.69	10.19	5.93	-5.86	-2.08	2.79	-8.06	11.78
SGRFG	-1.49	4.09	-2.48	3.54	-0.19	1.40	4.68	-4.35	-2.01	2.74	-6.51	9.91	5.82	-5.79	-2.48	3.29	-7.16	10.56
TGRFG	-0.86	3.24	-2.35	3.52	-1.27	2.90	5.58	-5.52	-2.48	3.54	-7.41	11.12	6.25	-6.36	-2.24	3.10	-8.06	11.78
VGRFG	-3.31	5.25	-2.50	4.85	6.01	1.40	-3.18	4.90	-2.48	4.70	6.90	0.00	-3.69	5.41	-2.45	4.57	6.71	0.19
NPNPC	-6.62	10.63	-3.57	4.24	4.38	-4.02	-10.73	16.40	-3.99	4.71	9.57	-11.59	-9.93	14.77	-2.75	2.96	10.34	-12.06
NPNPI	-4.97	8.96	-3.18	4.75	5.79	-6.36	-0.08	1.87	-3.12	4.60	-2.20	5.05	-1.53	4.13	-3.00	4.34	1.47	-0.37
NPNPM	7.84	-9.12	-3.35	4.53	-10.87	16.64	-0.51	2.29	-3.35	4.42	0.93	0.37	-0.83	2.72	-3.96	5.17	1.47	-0.37
NPNPP	-3.79	5.60	-3.99	4.71	6.12	-4.77	-2.36	3.58	-5.47	6.62	-1.92	5.98	3.73	-4.60	-2.75	2.96	-11.15	18.13
NPNPV	-2.74	5.38	-3.35	4.42	6.61	-7.38	-1.38	3.35	-3.67	4.80	4.19	-3.93	-1.64	3.34	-3.33	4.26	1.79	-0.19
NPNPY	-5.70	9.34	-3.38	4.68	8.07	-9.07	-3.79	6.33	-3.61	4.83	4.86	-4.30	-3.01	5.63	-3.96	5.17	7.61	-8.60
CPNPT	-3.75	5.44	-4.53	5.60	8.45	-8.13	-8.45	12.11	-4.53	5.60	14.69	-17.66	5.38	-6.64	-4.94	6.07	-9.23	14.67
IPNPT	-2.19	5.14	-3.52	5.17	-0.37	1.68	-1.51	4.12	-3.31	4.86	-0.73	2.15	5.08	-4.78	-3.31	4.79	-7.59	11.40
LPNPT	-1.55	4.38	-3.49	5.24	-0.99	2.62	-1.92	4.81	-3.41	5.10	0.00	1.21	-2.12	4.94	-3.52	5.17	0.46	0.75
MPNPT	3.74	-2.95	-3.35	4.73	-6.51	9.91	3.42	-2.58	-3.38	4.68	-5.52	8.50	5.22	-4.89	-3.48	4.75	-7.16	10.56
QPNPT	-3.50	4.99	-7.66	9.73	16.45	-15.61	-3.03	4.26	-3.67	4.22	-1.15	8.13	-3.38	4.68	-3.99	4.71	14.74	-13.27
VPNPT	-8.30	13.35	-3.35	4.42	9.80	-12.52	-0.88	3.25	-3.35	4.42	-1.04	2.06	-3.11	6.29	-3.96	5.17	2.04	-2.06
YPNPT	-2.33	5.31	-4.11	5.90	-0.37	1.68	3.97	-3.34	-4.12	5.78	-6.51	9.91	3.96	-3.17	-3.80	5.20	-6.25	9.35

**FIGURE 5:**
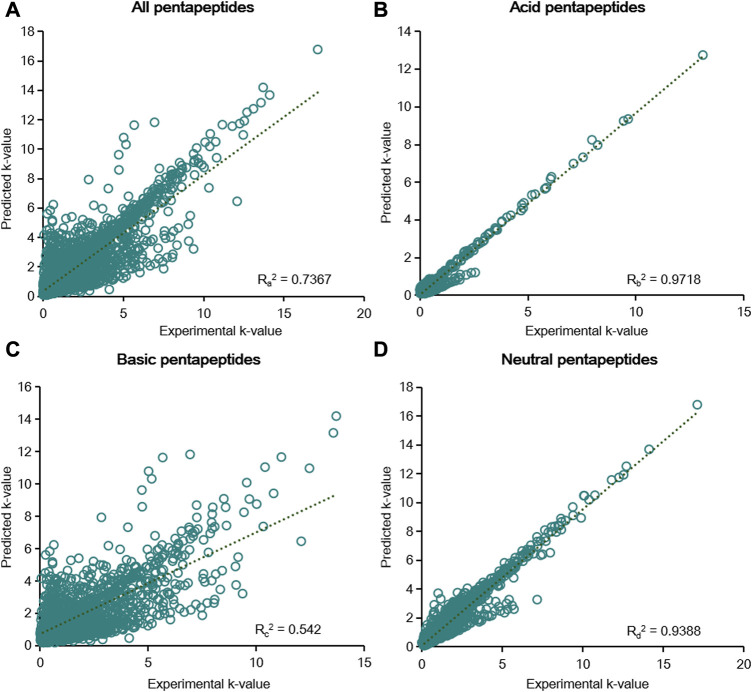
Linear fitting results of the predicted k-value and experimental k-value for all pentapeptides **(A)**, acid pentapeptides **(B)**, basic pentapeptides **(C)**, and neutral pentapeptides **(D)** in the six-parameter model of φ and pH.

**FIGURE 6:**
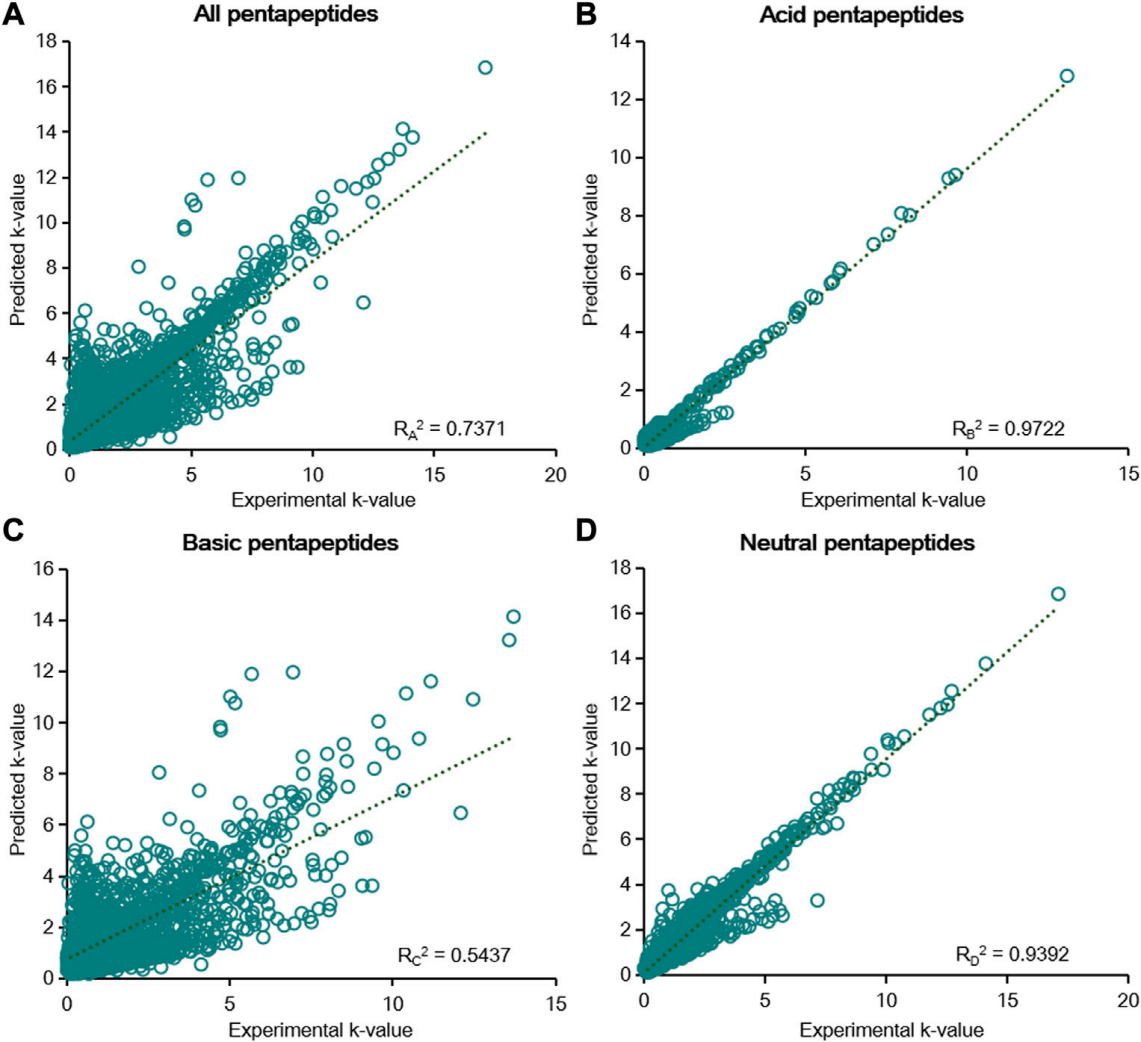
Linear fitting results of the predicted k-value and experimental k-value for all pentapeptides **(A)**, acid pentapeptides **(B)**, basic pentapeptides **(C)**, and neutral pentapeptides **(D)** in the six-parameter model of $P_{m}^{N}$ and pH.

The retention factors of the 57 pentapeptides under different elution conditions were predicted with φ or $P_{m}^{N}$ as the independent variables according to the six-parameter model, and 4,788 predicted k-values were obtained. We found that all data were distributed regularly around the axis of y = 0 by showing a residual scatter diagram depending on the used independent variable (φ or $P_{m}^{N}$ ) (Supplementary Figures S4A, B). We further fitted the data from the 57 pentapeptides between the experimental k-values and predicted k-values, and the R^2^ values were used to represent the correlation. When φ was used as the variable to describe the change in the mobile phase, the R_a_^2^ value was 0.7367 for all pentapeptides, indicating that the six-parameter model was not extremely suitable for the prediction of the chromatographic retention (Figure 5A). However, the six-parameter model exhibited extraordinary prediction capacity for acid and neutral pentapeptides since the R_b_^2^ value was 0.9718 for acid pentapeptides and the R_d_^2^ value was 0.9388 for neutral pentapeptides (Figures 5B, D). The R_c_^2^ value was lower than 0.6, indicating that the model was inappropriate for the basic pentapeptides. When $P_{m}^{N}$ was used as the variable to describe the change in the mobile phase, the results were very similar to the fitting results of φ (Figure 6A, R_A_^2^ = 0.7371; Figure 6B, R_B_^2^ = 0.9722; Figure 6C, R_C_^2^ = 0.7371; Figure 6D, R_D_^2^ = 0.7371). The correlation between the experimental k-values and predicted k-values was very good, and the model had an excellent capacity to predict the retention of the pentapeptides in RP-HPLC at different mobile phase compositions and pH values, especially for the acid and neutral pentapeptides. Moreover, the model results were always ideal regardless of the used variable (φ or $P_{m}^{N}$ ) to describe the change in the mobile phase, and the results of $P_{m}^{N}$ and pH were slightly better than those of φ and pH, consistent with a previous study. In addition, the prediction ability of the six-parameter model was evaluated under different chromatographic conditions (Supplementary Table S6). Higher T and $P_{m}^{N}$ correlated with more relevant results. The correlation of the six-parameter model was inversely proportional to φ. We reached the same conclusion as Section 4.4 specifically, that higher chromatographic retention was beneficial for the prediction capacity of the six-parameter model. Furthermore, 10-fold cross validation was conducted as described in Section 4.4. The residuals of the training and testing sets were randomly distributed around the y = 0 axis (Supplementary Figure S4), and the average RMSE of all pentapeptides was less than 0.8 in the six-parameter model. Moreover, the average RMSE of acid and neutral pentapeptides was approximately 0.3 (Supplementary Table S7), indicating the excellent prediction capacity of the six-parameter model for acid and neutral pentapeptides.

Retention behavior prediction of oligopeptides is valuable for efficient separation and purification. Previous studies have proposed various models to investigate the change in retention based on molecular descriptors or chromatographic theories (Park et al., 2020; Al Musaimi et al., 2023). There are five most commonly used models in studying the effect of mobile phase composition on retention behavior: (1) the linear-solvent-strength model, (2) the quadratic model, (3) the log–log (adsorption) model, (4) the mixed-mode model, and (5) the Neue–Kuss model (den Uijl et al., 2021). These models were able to predict the retention behavior at the first- and second-order levels. However, there was a clear deviation from linearity, especially in the lower organic modifier volume (Baeza-Baeza and García-Alvarez-Coque, 2020). Furthermore, the QSRR model displayed excellent prediction capacity for ionizable compounds but was limited to the type and calculation method of the molecular descriptors (Kumari et al., 2023). In addition, previous studies have reported the combined influence of temperature and mobile phase composition on chromatographic retention (Arkell et al., 2018; Caltabiano et al., 2018), and the simultaneous effect of pH and temperature or mobile phase composition has been less reported. The six-parameter model of pH and φ or $P_{m}^{N}$ performed better in predicting the capacity of acidic compounds than basic compounds in the mixture of water–acetonitrile (Agrafiotou et al., 2011). The combined effect of pH and mobile phase composition also depended on the molecular structure *via* response surface methodology (D’Archivio and Maggi, 2017). Here, we further proved that the six-parameter model was also suitable for acid or neutral compounds in a water–methanol system regardless of the pH and T or pH and φ or $P_{m}^{N}$ were used as independent variables.

## 5 Conclusion

Herein, we established six-parameter models *via* RP-HPLC data for predicting the retention factors of pentapeptides under different chromatographic conditions. The relationships of the three parameters pKa , $k_{HA}$ , and $k_{A}$ for all solutes derived from the sigmoidal model were studied against 1/T , φ, or $P_{m}^{N}$ . The results showed that good linear correlations existed between the $\log k_{HA}$ and $\log k_{A}$ with respect to either φ or $P_{m}^{N}$ for the acid and neutral pentapeptides, and 90% of the R^2^ values were greater than 0.9. Notably, the linear correlations with φ or $P_{m}^{N}$ as an independent variable were nearly identical. We then discussed in detail the effect of the distinct elements on the prediction capacity of the models by fitting the experimental k-values and predicted k-values in different groups of pentapeptides or chromatographic conditions. The R^2^ value was 0.8603 and the average RMSE was 0.2 for acid pentapeptides; the R^2^ values were less than 0.7 for the basic and neutral pentapeptides in the six-parameter model of pH and T, indicating that the model was not suitable to predict the change in the chromatographic retention for the basic and neutral pentapeptides at different pH and T. Moreover, the R^2^ values from the models with pH and X as independent variables were greater than 0.93, and the average RMSE was approximately 0.3 for the acid and neutral pentapeptides, indicating an effective prediction capacity of the chromatographic retention.

In this study, there are also some limitations. First, the fitting results of the model would be more reliable with more temperature gradients of chromatographic conditions, but only 3 gradients of column temperature were used in this study. Second, higher chromatographic retention correlated to better fitting results. However, we cannot ensure evident retention results for all studied pentapeptides under other diverse elution conditions. Third, we selected a narrow range of methanol concentrations to produce symmetrical and sharp chromatographic peaks. The methanol concentrations outside this range were undefined as to whether they followed the Soczewiński–Wachtmeister equation. Finally, both six-parameter models showed unsatisfactory prediction capability for the basic pentapeptides, which needs further research.

In conclusion, our study determined that the six-parameter model of pH and φ or $P_{m}^{N}$ was able to predict the chromatographic retention of the acid and neutral pentapeptides by analyzing the effect of various elements. Furthermore, our study could provide a methodological reference for the analysis and separation of pentapeptides with similar structures and polarities.

## Data Availability

The original contributions presented in the study are included in the article/Supplementary Material; further inquiries can be directed to the corresponding authors.

## Abbreviations

ADME, absorption, distribution, metabolism, and excretion; RP-HPLC, reversed-phase high-performance liquid chromatography; RPLC, reversed-phase liquid chromatography; SPPS, solid-phase synthesis; TFA, trifluoroacetic acid; pKa, acid dissociation constant; T, temperature; φ, organic modifier volume fraction; pHWWW , the pH measured in the aqueous buffer; pHWWS , the pH measured in the methanol–water mixture; pHSSS , the pH relative to the organic modifier–water solvent; $\log k_{w}$ , extrapolation retention factor; *k*, retention factor; $k_{HA}$ , the limiting retention factor of the protonated form; $k_{A}$ , the limiting retention factor of the dissociated form; $∆H^{0}$ , the D-value of enthalpy; $∆S^{0}$ , the D-value of entropy; Φ, the phase ratio; $P_{m}^{N}$ , the standard polarity parameter of the mobile phase; $P_{s}^{N}$ , the standard polarity parameter of the stationary phase; ${\left(logk\right)}_{0}$ , the retention factor when the polarity of the mobile phase is the same as that of the stationary phase; X, the variable describing the change in the mobile phase; R^2^, the correlation coefficient; and RMSE, the root mean squared error.
