# Supplementary material for: Simultaneous effect of different chromatographic conditions on the chromatographic retention of pentapeptide derivatives (HGRFG and NPNPT)
Source: Front Chem. 2023 Apr 18;11:1171824. doi: 10.3389/fchem.2023.1171824 (PMC10151710; doi:10.3389/fchem.2023.1171824)
Supplement: Supplementary file 1 [file DataSheet1.DOCX]

***Supplementary Material***

**Simultaneous effect of different chromatographic conditions on** **the** **chromatographic retention of pentapeptide derivatives (HGRFG, NPNPT)**

**Huan Peng^a,b^, Xiangrong Yang^b,f^, Huanle Fang^c^, Zhongqi Zhang^d^, Jinli Zhao^d^, Te Zhao^e^, Jianli Liu^b,c,*^，and Yan Li^a,*^**

**^*^Corresponding author:**

Yan Li, M.D. and Ph.D.: E-mail: [liyanxjtu@xjtu.edu.cn](mailto:liyanxjtu@xjtu.edu.cn)

Jianli Liu, Professor, PhD.: E-mail: [jlliu@nwu.edu.cn](mailto:jlliu@nwu.edu.cn)

**Supplementary Table 1.** The fitting results of $logk$ to $1/T$

| Pentapeptide sequence | pH | 8% | | | 10% | | | 12% | | | 14% | | |
| --- | --- | --- | --- | --- | --- | --- | --- | --- | --- | --- | --- | --- | --- |
|  |  | Slope | Inter-cept | R^2^ | Slope | Inter-cept | R^2^ | Slope | Inter-cept | R^2^ | Slope | Inter-cept | R^2^ |
| APNPT | pH=2 | 10.062 | 0.594 | 0.998 | 9.430 | 0.422 | 0.981 | 9.287 | 0.263 | 0.989 | 8.520 | 0.135 | 0.986 |
| DPNPT |  | 12.122 | 0.501 | 0.999 | 11.302 | 0.337 | 0.987 | 11.066 | 0.185 | 0.993 | 10.175 | 0.061 | 0.990 |
| EPNPT |  | 13.343 | 0.586 | 0.997 | 12.078 | 0.423 | 0.988 | 12.046 | 0.249 | 0.990 | 10.991 | 0.119 | 0.988 |
| GPNPT |  | 10.678 | 0.442 | 0.998 | 10.112 | 0.283 | 0.981 | 9.823 | 0.142 | 0.991 | 9.047 | 0.026 | 0.988 |
| HPNPT |  | 15.946 | 0.458 | 0.980 | 14.460 | 0.286 | 0.967 | 14.081 | 0.112 | 0.968 | 12.928 | -0.023 | 0.964 |
| KPNPT |  | 9.960 | 0.367 | 0.976 | 9.566 | 0.196 | 0.934 | 9.521 | 0.047 | 0.950 | 8.709 | -0.069 | 0.935 |
| NPNPT |  | 12.154 | 0.423 | 0.999 | 11.253 | 0.263 | 0.988 | 11.003 | 0.113 | 0.994 | 9.950 | -0.004 | 0.991 |
| PPNPT |  | 12.096 | 0.752 | 0.997 | 10.953 | 0.570 | 0.988 | 10.818 | 0.387 | 0.990 | 9.748 | 0.245 | 0.989 |
| RPNPT |  | 15.958 | 0.504 | 0.975 | 14.685 | 0.325 | 0.961 | 14.280 | 0.151 | 0.963 | 13.144 | 0.015 | 0.959 |
| SPNPT |  | 11.095 | 0.461 | 0.995 | 10.251 | 0.305 | 0.985 | 9.960 | 0.158 | 0.990 | 9.138 | 0.038 | 0.988 |
| TPNPT |  | 11.707 | 0.685 | 0.991 | 10.462 | 0.521 | 0.991 | 10.623 | 0.337 | 0.986 | 9.738 | 0.200 | 0.984 |
| NPNPA |  | 10.435 | 0.459 | 0.998 | 9.468 | 0.304 | 0.996 | 8.787 | 0.170 | 0.984 | 8.519 | 0.041 | 0.986 |
| NPNPD |  | 11.617 | 0.215 | 1.000 | 11.033 | 0.060 | 0.996 | 10.222 | -0.060 | 0.989 | 10.035 | -0.183 | 0.993 |
| NPNPE |  | 13.714 | 0.368 | 1.000 | 12.982 | 0.198 | 0.995 | 11.891 | 0.071 | 0.988 | 11.628 | -0.064 | 0.990 |
| NPNPG |  | 9.984 | 0.217 | 1.000 | 9.478 | 0.064 | 0.995 | 8.688 | -0.054 | 0.989 | 8.467 | -0.175 | 0.993 |
| NPNPH |  | 12.437 | 0.161 | 0.991 | 11.965 | -0.010 | 0.974 | 11.158 | -0.139 | 0.961 | 10.753 | -0.261 | 0.966 |
| NPNPK |  | 10.224 | 0.168 | 0.989 | 9.790 | -0.001 | 0.967 | 9.085 | -0.132 | 0.947 | 8.835 | -0.257 | 0.956 |
| NPNPN |  | 9.701 | 0.046 | 1.000 | 8.963 | -0.097 | 0.996 | 8.174 | -0.214 | 0.992 | 7.892 | -0.332 | 0.994 |
| NPNPQ |  | 11.039 | 0.155 | 1.000 | 10.139 | 0.005 | 0.996 | 9.322 | -0.122 | 0.992 | 8.859 | -0.243 | 0.995 |
| NPNPR |  | 14.800 | 0.264 | 0.988 | 13.981 | 0.085 | 0.974 | 12.942 | -0.054 | 0.965 | 12.434 | -0.191 | 0.968 |
| NPNPS |  | 10.998 | 0.160 | 1.000 | 10.289 | 0.011 | 0.995 | 10.262 | -0.129 | 0.996 | 9.010 | -0.227 | 0.993 |
| APNPT | pH=3 | 6.935 | 0.067 | 0.969 | 5.168 | -0.058 | 0.972 | 5.136 | -0.169 | 0.969 | 5.670 | -0.277 | 0.925 |
| DPNPT |  | 10.193 | 0.099 | 0.996 | 8.179 | -0.015 | 0.994 | 7.691 | -0.144 | 1.000 | 7.061 | -0.254 | 0.989 |
| EPNPT |  | 11.123 | 0.101 | 0.990 | 8.570 | -0.019 | 0.976 | 7.666 | -0.156 | 0.990 | 6.778 | -0.273 | 1.000 |
| GPNPT |  | 7.491 | -0.065 | 0.977 | 5.792 | -0.146 | 0.976 | 5.805 | -0.233 | 0.956 | 6.145 | -0.328 | 0.899 |
| HPNPT |  | 8.285 | -0.334 | 0.959 | 20.161 | -0.886 | 0.887 | 23.402 | -1.167 | 0.847 | 40.821 | -1.937 | 0.966 |
| KPNPT |  | 6.967 | -0.400 | 0.868 | 7.030 | -0.489 | 0.833 | 43.067 | -2.060 | 0.814 | 54.074 | -2.611 | 0.711 |
| NPNPT |  | 9.413 | -0.086 | 0.981 | 7.083 | -0.201 | 0.978 | 7.064 | -0.353 | 0.997 | 8.749 | -0.522 | 0.960 |
| PPNPT |  | 9.696 | 0.232 | 0.978 | 7.002 | 0.100 | 0.950 | 6.166 | -0.050 | 0.966 | 5.573 | -0.184 | 1.000 |
| RPNPT |  | 8.372 | -0.335 | 0.973 | 21.577 | -0.928 | 0.865 | 30.454 | -1.421 | 0.957 | 46.448 | -2.165 | 0.916 |
| SPNPT |  | 9.134 | -0.037 | 0.990 | 7.070 | -0.153 | 0.983 | 6.029 | -0.257 | 0.999 | 9.706 | -0.496 | 0.988 |
| TPNPT |  | 8.549 | 0.164 | 0.968 | 6.001 | 0.040 | 0.927 | 5.540 | -0.104 | 0.990 | 5.762 | -0.218 | 0.965 |
| NPNPA |  | 7.547 | -0.029 | 0.962 | 5.618 | -0.132 | 0.999 | 5.094 | -0.213 | 0.955 | 5.583 | -0.311 | 0.915 |
| NPNPD |  | 9.854 | -0.225 | 0.996 | 7.513 | -0.305 | 0.996 | 11.675 | -0.570 | 0.980 | 15.094 | -0.812 | 0.948 |
| NPNPE |  | 11.887 | -0.109 | 0.988 | 10.075 | -0.242 | 0.993 | 8.337 | -0.332 | 1.000 | 12.205 | -0.595 | 0.996 |
| NPNPG |  | 8.450 | -0.243 | 0.999 | 6.457 | -0.313 | 0.986 | 14.164 | -0.721 | 0.971 | 17.003 | -0.925 | 0.912 |
| NPNPH |  | 18.413 | -0.927 | 0.893 | 15.803 | -1.110 | 0.927 | 28.590 | -1.723 | 0.902 | 41.253 | -2.413 | 0.765 |
| NPNPK |  | 15.405 | -1.059 | 0.922 | 31.972 | -1.863 | 0.912 | 48.527 | -2.957 | 0.700 | \ | \ | \ |
| NPNPN |  | 7.823 | -0.352 | 0.996 | 12.520 | -0.674 | 0.973 | 7.418 | -0.697 | 0.909 | 3.999 | -0.697 | 0.447 |
| NPNPQ |  | 8.404 | -0.263 | 0.997 | 6.765 | -0.339 | 0.969 | 14.805 | -0.738 | 0.923 | 18.081 | -0.995 | 0.868 |
| NPNPR |  | 20.307 | -0.856 | 0.901 | 22.809 | -1.119 | 0.878 | 37.590 | -1.775 | 0.998 | 50.799 | -2.445 | 0.861 |
| NPNPS |  | 8.877 | -0.287 | 1.000 | 6.720 | -0.352 | 0.970 | 14.746 | -0.779 | 0.953 | 17.647 | -0.980 | 0.869 |
| APNPT | pH=4 | 6.566 | 0.075 | 0.927 | 5.769 | -0.073 | 0.938 | 4.974 | -0.198 | 0.959 | 4.224 | -0.302 | 1.000 |
| DPNPT |  | 9.170 | 0.010 | 1.000 | 7.820 | -0.110 | 0.997 | 6.977 | -0.221 | 0.981 | 6.134 | -0.320 | 0.956 |
| EPNPT |  | 8.529 | 0.073 | 0.970 | 7.341 | -0.072 | 0.973 | 5.599 | -0.181 | 0.955 | 4.988 | -0.306 | 0.976 |
| GPNPT |  | 7.210 | -0.038 | 0.948 | 6.374 | -0.176 | 0.959 | 5.761 | -0.299 | 0.979 | 5.427 | -0.408 | 0.999 |
| HPNPT |  | 5.229 | -0.135 | 0.934 | 2.816 | -0.260 | 0.999 | 2.496 | -0.382 | 0.625 | -4.067 | -0.369 | 0.486 |
| KPNPT |  | 2.477 | -0.367 | 0.927 | -9.050 | -0.230 | 0.769 | -1.791 | -0.578 | 0.554 | -10.801 | -0.489 | 0.712 |
| NPNPT |  | 8.766 | -0.082 | 0.960 | 8.221 | -0.233 | 0.975 | 7.414 | -0.347 | 0.993 | 8.878 | -0.513 | 0.997 |
| PPNPT |  | 8.008 | 0.230 | 0.937 | 7.035 | 0.063 | 0.946 | 5.948 | -0.076 | 0.949 | 4.968 | -0.206 | 0.959 |
| RPNPT |  | 8.630 | -0.356 | 0.932 | 5.177 | -0.405 | 0.997 | 4.372 | -0.482 | 0.939 | -8.303 | -0.337 | 0.491 |
| SPNPT |  | 7.562 | -0.036 | 0.952 | 6.747 | -0.179 | 0.967 | 5.916 | -0.297 | 0.988 | 5.368 | -0.404 | 0.997 |
| TPNPT |  | 9.383 | 0.127 | 0.898 | 7.230 | 0.016 | 0.946 | 6.329 | -0.121 | 0.966 | 5.718 | -0.254 | 0.978 |
| NPNPA |  | 6.699 | 0.000 | 0.928 | 6.275 | -0.142 | 0.948 | 5.786 | -0.269 | 0.967 | 5.295 | -0.375 | 0.998 |
| NPNPD |  | 8.779 | -0.227 | 0.980 | 8.339 | -0.378 | 0.990 | 7.908 | -0.504 | 0.998 | 8.107 | -0.627 | 0.999 |
| NPNPE |  | 10.175 | -0.133 | 0.967 | 9.509 | -0.288 | 0.978 | 8.784 | -0.419 | 0.986 | 8.232 | -0.536 | 0.998 |
| NPNPG |  | 7.719 | -0.219 | 0.958 | 7.344 | -0.369 | 0.981 | 6.985 | -0.489 | 1.000 | 5.303 | -0.569 | 0.997 |
| NPNPH |  | 5.966 | -0.454 | 0.994 | 7.129 | -0.705 | 0.980 | -0.369 | -0.605 | 0.057 | -0.588 | -0.666 | 0.036 |
| NPNPK |  | 5.105 | -0.583 | 0.867 | -0.463 | -0.568 | 0.098 | -0.069 | -0.661 | 0.001 | -1.327 | -0.943 | 0.035 |
| NPNPN |  | 8.685 | -0.392 | 0.993 | 6.000 | -0.472 | 1.000 | 6.183 | -0.623 | 0.999 | 4.698 | -0.723 | 0.965 |
| NPNPQ |  | 8.556 | -0.293 | 0.978 | 8.463 | -0.436 | 0.999 | 4.388 | -0.457 | 0.927 | 2.517 | -0.539 | 0.455 |
| NPNPR |  | 9.084 | -0.438 | 0.948 | 5.562 | -0.496 | 0.997 | 6.236 | -0.741 | 0.981 | -0.604 | -0.641 | 0.067 |
| NPNPS |  | 8.213 | -0.313 | 0.983 | 6.576 | -0.412 | 0.987 | 5.209 | -0.506 | 0.961 | 5.210 | -0.659 | 0.975 |
| APNPT | pH=5 | 3.497 | 0.031 | 0.993 | 2.564 | -0.091 | 0.987 | 2.325 | -0.206 | 0.952 | 3.910 | -0.370 | 0.410 |
| DPNPT |  | 4.000 | -0.105 | 0.891 | 3.648 | -0.185 | 0.869 | 24.318 | -1.121 | 0.921 | 25.039 | -1.209 | 0.910 |
| EPNPT |  | 1.749 | -0.043 | 0.772 | -10.495 | 0.075 | 0.700 | -0.605 | -0.443 | 0.154 | -2.752 | -0.466 | 0.878 |
| GPNPT |  | 4.685 | -0.077 | 0.990 | 3.790 | -0.182 | 0.966 | 3.776 | -0.278 | 0.956 | 3.931 | -0.359 | 0.981 |
| HPNPT |  | 6.534 | 0.137 | 0.960 | 4.369 | -0.002 | 0.921 | 1.079 | -0.260 | 0.120 | 1.774 | -0.325 | 0.131 |
| KPNPT |  | 0.693 | -0.326 | 0.731 | 0.112 | -0.401 | 0.226 | 0.230 | -0.484 | 0.970 | 0.227 | -0.556 | 0.723 |
| NPNPT |  | 6.304 | -0.128 | 0.998 | 5.319 | -0.254 | 0.999 | 4.603 | -0.375 | 0.999 | 3.636 | -0.472 | 0.996 |
| PPNPT |  | 5.473 | 0.160 | 0.996 | 4.022 | 0.027 | 0.987 | 3.191 | -0.103 | 0.980 | 2.752 | -0.227 | 0.992 |
| RPNPT |  | 4.657 | -0.303 | 0.974 | 3.756 | -0.377 | 0.945 | 3.379 | -0.464 | 0.966 | 2.860 | -0.537 | 0.959 |
| SPNPT |  | 5.026 | -0.103 | 0.999 | 4.101 | -0.227 | 0.996 | 3.269 | -0.341 | 1.000 | 2.546 | -0.443 | 0.993 |
| TPNPT |  | 5.265 | 0.111 | 0.997 | 4.141 | -0.021 | 0.991 | 3.681 | -0.150 | 0.992 | 3.883 | -0.293 | 0.704 |
| NPNPA |  | 4.858 | -0.069 | 0.999 | 3.873 | -0.174 | 0.994 | 3.396 | -0.270 | 0.965 | 2.917 | -0.372 | 0.371 |
| NPNPD |  | 8.082 | -0.423 | 0.179 | -5.306 | -0.292 | 0.611 | 0.897 | -0.610 | 0.420 | -0.889 | -0.629 | 0.262 |
| NPNPE |  | 7.842 | -0.355 | 0.274 | 5.350 | -0.571 | 0.966 | 2.436 | -0.600 | 0.939 | 0.778 | -0.648 | 0.169 |
| NPNPG |  | 6.365 | -0.255 | 1.000 | 5.442 | -0.375 | 1.000 | 4.773 | -0.487 | 1.000 | 4.046 | -0.584 | 0.990 |
| NPNPH |  | 4.447 | -0.378 | 0.945 | 3.883 | -0.461 | 0.970 | 3.491 | -0.536 | 0.972 | 9.613 | -0.885 | 0.711 |
| NPNPK |  | 2.272 | -0.411 | 0.990 | 3.334 | -0.653 | 0.626 | 0.196 | -0.655 | 0.061 | 4.222 | -0.899 | 0.990 |
| NPNPN |  | 5.944 | -0.365 | 1.000 | 4.763 | -0.481 | 1.000 | 3.957 | -0.590 | 0.999 | 2.684 | -0.664 | 0.885 |
| NPNPQ |  | 6.617 | -0.305 | 1.000 | 7.857 | -0.503 | 0.992 | 5.029 | -0.567 | 0.998 | 4.008 | -0.660 | 0.988 |
| NPNPR |  | 5.498 | -0.349 | 0.924 | 4.543 | -0.420 | 0.955 | 4.392 | -0.511 | 0.981 | 3.919 | -0.584 | 0.986 |
| NPNPS |  | 6.048 | -0.342 | 1.000 | 4.735 | -0.452 | 0.999 | 4.193 | -0.566 | 0.992 | 2.856 | -0.640 | 0.961 |
| APNPT | pH=6 | 4.871 | -0.044 | 0.998 | 4.239 | -0.198 | 1.000 | 4.274 | -0.354 | 0.996 | 3.303 | -0.473 | 0.986 |
| DPNPT |  | 7.685 | -0.438 | 0.995 | 7.739 | -0.607 | 0.994 | 7.168 | -0.775 | 1.000 | 8.993 | -0.955 | 0.969 |
| EPNPT |  | -0.097 | -0.231 | 0.003 | -0.459 | -0.426 | 0.125 | -1.270 | -0.561 | 0.855 | -0.435 | -0.762 | 0.014 |
| GPNPT |  | 6.856 | -0.199 | 0.996 | 6.180 | -0.347 | 0.999 | 5.871 | -0.491 | 1.000 | 5.072 | -0.617 | 0.992 |
| HPNPT |  | 11.141 | 0.133 | 0.975 | 10.193 | -0.041 | 0.977 | 9.921 | -0.212 | 0.987 | 10.224 | -0.396 | 0.992 |
| KPNPT |  | 0.630 | -0.350 | 0.337 | -1.930 | -0.448 | 0.346 | -2.337 | -0.650 | 0.547 | 1.063 | -0.868 | 0.059 |
| NPNPT |  | 7.655 | -0.169 | 0.997 | 7.079 | -0.327 | 0.999 | 6.235 | -0.457 | 0.996 | 5.563 | -0.594 | 0.936 |
| PPNPT |  | 6.396 | 0.097 | 0.991 | 5.104 | -0.057 | 0.992 | 3.866 | -0.195 | 0.986 | 3.120 | -0.336 | 0.998 |
| RPNPT |  | 5.089 | -0.150 | 0.991 | 3.857 | -0.313 | 0.950 | 3.291 | -0.472 | 0.922 | 2.088 | -0.595 | 0.328 |
| SPNPT |  | 6.213 | -0.158 | 0.998 | 5.514 | -0.307 | 1.000 | 5.043 | -0.450 | 1.000 | 4.589 | -0.589 | 0.972 |
| TPNPT |  | 5.930 | 0.091 | 0.996 | 5.205 | -0.070 | 1.000 | 4.643 | -0.217 | 1.000 | 3.943 | -0.352 | 0.984 |
| NPNPA |  | 6.112 | -0.135 | 0.994 | 5.293 | -0.262 | 1.000 | 4.921 | -0.399 | 1.000 | 4.594 | -0.528 | 0.996 |
| NPNPD |  | 8.850 | -0.811 | 1.000 | 5.086 | -0.866 | 0.872 | 3.492 | -0.914 | 0.777 | 15.614 | -1.446 | 0.965 |
| NPNPE |  | 9.420 | -0.768 | 0.998 | 8.206 | -0.917 | 0.986 | 3.478 | -0.898 | 0.713 | 7.020 | -1.103 | 0.984 |
| NPNPG |  | 7.503 | -0.307 | 0.999 | 6.869 | -0.456 | 1.000 | 6.911 | -0.618 | 0.999 | 7.039 | -0.774 | 0.994 |
| NPNPH |  | 5.262 | -0.228 | 1.000 | 4.477 | -0.380 | 0.994 | 4.240 | -0.532 | 0.976 | 3.646 | -0.665 | 0.864 |
| NPNPK |  | 3.319 | -0.375 | 0.962 | 2.097 | -0.521 | 0.636 | 2.165 | -0.713 | 0.647 | 2.227 | -0.875 | 0.557 |
| NPNPN |  | 7.600 | -0.435 | 1.000 | 7.194 | -0.607 | 1.000 | 6.768 | -0.762 | 0.996 | 6.404 | -0.913 | 0.970 |
| NPNPQ |  | 8.012 | -0.368 | 0.999 | 7.687 | -0.539 | 1.000 | 8.619 | -0.750 | 1.000 | 6.966 | -0.859 | 0.996 |
| NPNPR |  | 7.742 | -0.233 | 1.000 | 6.731 | -0.400 | 0.999 | 6.017 | -0.555 | 0.989 | 6.298 | -0.738 | 0.958 |
| NPNPS |  | 7.250 | -0.397 | 0.999 | 6.646 | -0.552 | 0.999 | 6.563 | -0.716 | 0.999 | 5.848 | -0.847 | 0.924 |
| APNPT | pH=7 | 3.664 | 0.134 | 0.986 | 3.049 | 0.007 | 0.997 | 2.609 | -0.104 | 0.984 | 2.357 | -0.210 | 0.921 |
| DPNPT |  | 7.492 | -0.622 | 0.776 | 6.841 | -0.785 | 0.994 | 6.343 | -0.963 | 0.914 | 27.149 | -1.926 | 0.825 |
| EPNPT |  | 6.559 | -0.604 | 0.736 | 2.329 | -0.711 | 0.794 | 1.892 | -0.900 | 0.650 | 14.746 | -1.577 | 0.861 |
| GPNPT |  | 5.428 | -0.016 | 0.990 | 4.849 | -0.132 | 1.000 | 4.121 | -0.224 | 0.989 | 3.857 | -0.319 | 0.950 |
| HPNPT |  | 13.454 | 0.201 | 0.986 | 11.977 | 0.060 | 0.993 | 11.325 | -0.083 | 0.990 | 11.558 | -0.241 | 0.999 |
| KPNPT |  | 3.097 | 0.265 | 0.977 | 5.020 | 0.084 | 0.999 | 1.472 | 0.070 | 0.446 | 2.838 | -0.034 | 0.732 |
| NPNPT |  | 9.636 | -0.240 | 0.982 | 8.867 | -0.382 | 0.994 | 6.942 | -0.462 | 0.992 | 4.290 | -0.497 | 0.828 |
| PPNPT |  | 5.222 | 0.335 | 0.988 | 4.339 | 0.203 | 0.995 | 3.579 | 0.090 | 0.996 | 2.823 | -0.002 | 0.974 |
| RPNPT |  | 7.524 | 0.364 | 1.000 | 7.186 | 0.226 | 0.999 | 7.271 | 0.096 | 0.999 | 7.406 | -0.025 | 0.965 |
| SPNPT |  | 8.339 | -0.162 | 0.986 | 7.638 | -0.300 | 0.997 | 6.816 | -0.404 | 0.998 | 5.870 | -0.495 | 0.957 |
| TPNPT |  | 6.487 | 0.122 | 0.997 | 8.153 | -0.098 | 0.886 | 6.464 | -0.216 | 0.998 | 6.029 | -0.343 | 0.995 |
| NPNPA |  | 8.252 | -0.216 | 0.993 | 6.657 | -0.297 | 1.000 | 7.877 | -0.470 | 0.957 | 2.704 | -0.384 | 0.768 |
| NPNPD |  | 50.965 | -2.919 | 0.993 | \ | \ | \ | \ | \ | \ | \ | \ | \ |
| NPNPE |  | 34.074 | -2.118 | 0.846 | \ | \ | \ | \ | \ | \ | \ | \ | \ |
| NPNPG |  | 9.357 | -0.348 | 0.980 | 8.298 | -0.468 | 1.000 | 5.440 | -0.501 | 0.891 | 8.601 | -0.725 | 0.926 |
| NPNPH |  | 12.182 | -0.158 | 0.984 | 13.243 | -0.346 | 1.000 | 11.401 | -0.430 | 1.000 | 11.077 | -0.545 | 0.995 |
| NPNPK |  | 6.330 | -0.006 | 0.988 | 5.938 | -0.122 | 0.957 | 5.630 | -0.218 | 0.923 | 5.157 | -0.295 | 0.874 |
| NPNPN |  | 9.076 | -0.426 | 0.992 | 6.861 | -0.502 | 0.988 | 8.638 | -0.695 | 0.918 | 12.204 | -0.936 | 0.928 |
| NPNPQ |  | 9.599 | -0.379 | 0.988 | 7.913 | -0.486 | 1.000 | 4.519 | -0.497 | 0.866 | 9.102 | -0.779 | 0.888 |
| NPNPR |  | 9.729 | 0.086 | 1.000 | 8.842 | -0.039 | 0.996 | 7.515 | -0.128 | 0.992 | 7.347 | -0.232 | 0.963 |
| NPNPS |  | 8.770 | -0.399 | 0.989 | 6.961 | -0.488 | 0.995 | 7.986 | -0.652 | 0.934 | 10.524 | -0.851 | 0.921 |
| APNPT | pH=8 | 2.674 | 0.288 | 0.536 | 1.746 | 0.141 | 0.388 | 0.730 | 0.018 | 0.198 | -1.224 | -0.070 | 0.508 |
| DPNPT |  | 9.539 | -0.589 | 0.769 | 0.985 | -0.542 | 0.081 | -2.073 | -0.605 | 0.789 | 8.782 | -1.182 | 0.515 |
| EPNPT |  | 2.125 | -0.414 | 0.116 | -4.448 | -0.422 | 0.648 | 2.684 | -0.886 | 0.119 | 9.310 | -1.253 | 0.366 |
| GPNPT |  | 3.432 | 0.065 | 0.848 | 2.350 | -0.068 | 0.816 | 0.523 | -0.159 | 0.709 | -1.220 | -0.250 | 0.859 |
| HPNPT |  | 14.179 | 0.305 | 0.904 | 12.612 | 0.148 | 0.902 | 11.255 | 0.007 | 0.906 | 10.023 | -0.125 | 0.899 |
| KPNPT |  | 0.239 | 0.213 | 0.014 | -1.449 | 0.094 | 0.205 | -3.536 | 0.003 | 0.521 | -6.884 | -0.040 | 0.606 |
| NPNPT |  | 8.874 | -0.094 | 0.897 | 6.723 | -0.209 | 0.913 | 5.518 | -0.340 | 0.936 | 4.087 | -0.449 | 0.968 |
| PPNPT |  | -3.408 | 0.792 | 0.831 | -3.200 | 0.605 | 0.933 | -4.518 | 0.500 | 0.869 | -4.473 | 0.363 | 0.843 |
| RPNPT |  | 7.744 | 0.260 | 0.992 | 5.784 | 0.127 | 0.935 | 3.615 | 0.024 | 0.671 | 2.012 | -0.084 | 0.282 |
| SPNPT |  | 6.938 | -0.030 | 0.866 | 5.174 | -0.150 | 0.880 | 3.971 | -0.274 | 0.894 | 2.957 | -0.393 | 0.896 |
| TPNPT |  | 6.955 | 0.224 | 0.803 | 5.546 | 0.078 | 0.783 | 3.591 | -0.028 | 0.758 | 2.700 | -0.159 | 0.755 |
| NPNPA |  | 7.348 | -0.069 | 0.846 | 5.474 | -0.178 | 0.895 | 4.379 | -0.299 | 0.941 | 3.031 | -0.399 | 1.000 |
| NPNPD |  | 9.430 | -0.963 | 0.532 | 16.539 | -1.397 | 0.509 | \ | \ | \ | \ | \ | \ |
| NPNPE |  | 1.364 | -0.566 | 0.434 | 11.989 | -1.182 | 0.500 | 45.088 | -2.545 | 0.580 | \ | \ | \ |
| NPNPG |  | 8.151 | -0.198 | 0.925 | 7.179 | -0.348 | 0.919 | 5.743 | -0.462 | 0.962 | 3.164 | -0.541 | 0.993 |
| NPNPH |  | 11.126 | -0.095 | 0.926 | 9.646 | -0.235 | 0.928 | 8.432 | -0.362 | 0.957 | 6.824 | -0.464 | 0.986 |
| NPNPK |  | 4.334 | -0.108 | 0.886 | 2.200 | -0.213 | 0.385 | 0.207 | -0.307 | 0.002 | -1.931 | -0.383 | 0.096 |
| NPNPN |  | 8.057 | -0.289 | 0.936 | 7.023 | -0.443 | 0.939 | 5.016 | -0.549 | 0.948 | 0.727 | -0.583 | 0.242 |
| NPNPQ |  | 8.605 | -0.236 | 0.925 | 7.345 | -0.387 | 0.938 | 6.041 | -0.515 | 0.968 | 3.417 | -0.611 | 0.940 |
| NPNPR |  | 9.168 | -0.025 | 0.998 | 7.225 | -0.153 | 0.969 | 4.951 | -0.247 | 0.760 | 2.761 | -0.332 | 0.297 |
| NPNPS |  | 7.853 | -0.262 | 0.930 | 6.739 | -0.406 | 0.928 | 4.915 | -0.513 | 0.966 | 1.566 | -0.574 | 0.648 |
| Pentapeptide sequence | pH | 20% | | | 22% | | | 24% | | | 26% | | |
|  |  | Slope | Inter-cept | R^2^ | Slope | Inter-cept | R^2^ | Slope | Inter-cept | R^2^ | Slope | Inter-cept | R^2^ |
| HGRFA | pH=2 | 18.181 | 0.249 | 0.914 | 17.476 | 0.148 | 0.918 | 15.084 | 0.084 | 0.870 | 14.607 | -0.021 | 0.869 |
| HGRFD |  | 21.678 | 0.081 | 0.939 | 20.817 | -0.016 | 0.940 | 18.289 | -0.073 | 0.907 | 17.602 | -0.172 | 0.906 |
| HGRFE |  | 19.805 | 0.133 | 0.932 | 19.221 | 0.025 | 0.935 | 16.778 | -0.036 | 0.901 | 16.190 | -0.140 | 0.906 |
| HGRFG |  | 18.705 | 0.149 | 0.920 | 18.159 | 0.047 | 0.922 | 9.154 | 0.239 | 1.000 | 14.892 | -0.100 | 0.870 |
| HGRFH |  | 19.717 | -0.052 | 0.910 | 18.862 | -0.152 | 0.909 | 15.716 | -0.196 | 0.839 | 15.166 | -0.302 | 0.847 |
| HGRFK |  | 18.387 | -0.076 | 0.888 | 17.744 | -0.177 | 0.893 | 14.204 | -0.204 | 0.811 | 13.503 | -0.301 | 0.820 |
| HGRFN |  | 18.326 | -0.038 | 0.935 | 17.681 | -0.135 | 0.934 | 15.242 | -0.186 | 0.892 | 14.738 | -0.284 | 0.884 |
| HGRFQ |  | 18.553 | 0.034 | 0.932 | 17.938 | -0.068 | 0.933 | 15.563 | -0.126 | 0.898 | 15.007 | -0.227 | 0.894 |
| HGRFR |  | 20.639 | -0.022 | 0.910 | 19.838 | -0.130 | 0.916 | 16.319 | -0.169 | 0.857 | 15.496 | -0.272 | 0.870 |
| HGRFS |  | 19.101 | 0.053 | 0.932 | 18.395 | -0.045 | 0.930 | 15.891 | -0.098 | 0.891 | 15.342 | -0.198 | 0.882 |
| HGRFT |  | 19.800 | 0.200 | 0.930 | 19.244 | 0.091 | 0.933 | 16.519 | 0.035 | 0.891 | 16.023 | -0.075 | 0.884 |
| AGRFG |  | 15.727 | 0.233 | 0.947 | 15.437 | 0.134 | 0.945 | 14.012 | 0.064 | 0.928 | 13.530 | -0.026 | 0.907 |
| DGRFG |  | 16.464 | 0.186 | 0.955 | 15.900 | 0.096 | 0.957 | 14.518 | 0.026 | 0.940 | 14.175 | -0.069 | 0.924 |
| EGRFG |  | 16.854 | 0.224 | 0.951 | 16.288 | 0.129 | 0.953 | 14.862 | 0.056 | 0.935 | 14.396 | -0.038 | 0.917 |
| GGRFG |  | 15.647 | 0.142 | 0.935 | 15.476 | 0.042 | 0.938 | 13.877 | -0.018 | 0.924 | 13.417 | -0.106 | 0.905 |
| KGRFG |  | 17.566 | 0.160 | 0.926 | 17.307 | 0.053 | 0.928 | 15.023 | -0.003 | 0.889 | 14.630 | -0.104 | 0.882 |
| NGRFG |  | 16.133 | 0.169 | 0.949 | 15.663 | 0.076 | 0.951 | 14.296 | 0.005 | 0.933 | 13.941 | -0.089 | 0.917 |
| PGRFG |  | 16.378 | 0.317 | 0.948 | 15.906 | 0.212 | 0.950 | 14.511 | 0.133 | 0.933 | 13.926 | 0.037 | 0.914 |
| QGRFG |  | 16.276 | 0.226 | 0.947 | 15.851 | 0.134 | 0.949 | 14.364 | 0.068 | 0.928 | 13.942 | -0.022 | 0.911 |
| RGRFG |  | 19.126 | 0.187 | 0.933 | 18.798 | 0.073 | 0.931 | 16.077 | 0.025 | 0.895 | 15.388 | -0.078 | 0.897 |
| SGRFG |  | 15.863 | 0.185 | 0.944 | 15.434 | 0.091 | 0.947 | 14.018 | 0.023 | 0.925 | 13.652 | -0.070 | 0.909 |
| TGRFG |  | 15.969 | 0.231 | 0.941 | 23.028 | -0.144 | 0.840 | 14.017 | 0.063 | 0.920 | 13.570 | -0.030 | 0.903 |
| VGRFG |  | 15.453 | 0.545 | 0.914 | 16.348 | 0.397 | 0.942 | 14.795 | 0.312 | 0.922 | 14.398 | 0.202 | 0.897 |
| NPNPC |  | 20.687 | 0.106 | 0.976 | 19.116 | -0.041 | 0.974 | 17.311 | -0.178 | 0.974 | 15.962 | -0.307 | 0.935 |
| NPNPI |  | 10.498 | 0.684 | 0.972 | 8.670 | 0.628 | 0.993 | 9.296 | 0.486 | 0.986 | 8.670 | 0.399 | 0.918 |
| NPNPM |  | 11.648 | 0.296 | 0.979 | 11.090 | 0.199 | 0.978 | 10.724 | 0.099 | 0.980 | 10.151 | 0.015 | 0.930 |
| NPNPP |  | 9.574 | 0.027 | 0.984 | 8.803 | -0.070 | 0.981 | 8.342 | -0.171 | 0.986 | 7.592 | -0.252 | 0.908 |
| NPNPV |  | 8.690 | 0.330 | 0.976 | 8.365 | 0.229 | 0.972 | 8.195 | 0.128 | 0.977 | 7.691 | 0.047 | 0.901 |
| NPNPY |  | 15.681 | 0.221 | 0.986 | 15.333 | 0.112 | 0.984 | 14.848 | 0.010 | 0.984 | 14.140 | -0.079 | 0.964 |
| CPNPT |  | 8.870 | -0.049 | 0.983 | 8.319 | -0.144 | 0.981 | 7.869 | -0.235 | 0.982 | 7.281 | -0.312 | 0.921 |
| IPNPT |  | 10.671 | 0.531 | 0.966 | 10.016 | 0.403 | 0.965 | 9.695 | 0.271 | 0.970 | 8.855 | 0.167 | 0.882 |
| LPNPT |  | 11.143 | 0.633 | 0.967 | 10.522 | 0.503 | 0.965 | 10.294 | 0.366 | 0.973 | 9.539 | 0.257 | 0.890 |
| MPNPT |  | 11.969 | 0.349 | 0.975 | 11.338 | 0.231 | 0.974 | 10.840 | 0.114 | 0.979 | 10.073 | 0.016 | 0.919 |
| QPNPT |  | 8.124 | 0.007 | 1.000 | 7.448 | -0.095 | 1.000 | 7.397 | -0.207 | 0.999 | 6.558 | -0.287 | 0.975 |
| VPNPT |  | 9.084 | 0.213 | 0.975 | 8.388 | 0.101 | 0.973 | 7.906 | -0.010 | 0.979 | 7.161 | -0.101 | 0.883 |
| YPNPT |  | 16.950 | 0.261 | 0.988 | 16.102 | 0.132 | 0.985 | 15.458 | 0.006 | 0.990 | 14.227 | -0.092 | 0.961 |
| HGRFA | pH=3 | 49.472 | -2.022 | 0.767 | 50.464 | -2.117 | 0.772 | 49.413 | -2.142 | 0.786 | 53.458 | -2.340 | 0.803 |
| HGRFD |  | 12.748 | -0.581 | 0.963 | 13.077 | -0.645 | 0.964 | 13.136 | -0.707 | 0.964 | 14.679 | -0.807 | 0.961 |
| HGRFE |  | 12.455 | -0.630 | 0.961 | 12.876 | -0.701 | 0.963 | 13.649 | -0.790 | 0.974 | 14.588 | -0.868 | 0.948 |
| HGRFG |  | 25.102 | -1.219 | 0.853 | 24.060 | -1.245 | 0.819 | 23.264 | -1.275 | 0.804 | 43.431 | -2.063 | 0.992 |
| HGRFH |  | 13.993 | -0.843 | 0.890 | 12.823 | -0.850 | 0.818 | 12.049 | -0.872 | 0.764 | 11.180 | -0.867 | 0.766 |
| HGRFK |  | 15.063 | -0.846 | 0.971 | 14.793 | -0.879 | 0.957 | 14.858 | -0.942 | 0.819 | 15.898 | -1.012 | 0.844 |
| HGRFN |  | 97.149 | -4.259 | 0.965 | \ | \ | \ | \ | \ | \ | \ | \ | \ |
| HGRFQ |  | \ | \ | \ | \ | \ | \ | \ | \ | \ | \ | \ | \ |
| HGRFR |  | 16.096 | -0.850 | 0.973 | 15.452 | -0.879 | 0.951 | 15.564 | -0.946 | 0.862 | 16.340 | -1.012 | 0.863 |
| HGRFS |  | 84.416 | -3.721 | 0.985 | \ | \ | \ | \ | \ | \ | \ | \ | \ |
| HGRFT |  | 54.862 | -2.406 | 0.997 | 63.997 | -2.877 | 0.982 | \ | \ | \ | \ | \ | \ |
| AGRFG |  | 19.880 | -0.831 | 0.918 | 23.034 | -1.006 | 0.891 | 23.945 | -1.107 | 0.894 | 24.399 | -1.171 | 0.936 |
| DGRFG |  | 11.389 | -0.392 | 1.000 | 13.507 | -0.555 | 0.986 | 14.657 | -0.680 | 0.982 | 18.036 | -0.881 | 0.981 |
| EGRFG |  | 16.154 | -0.680 | 0.974 | 18.970 | -0.880 | 0.990 | 20.770 | -1.039 | 0.967 | 23.999 | -1.224 | 0.927 |
| GGRFG |  | 22.349 | -0.936 | 0.900 | 23.247 | -1.029 | 0.905 | 23.620 | -1.109 | 0.913 | 27.038 | -1.358 | 0.824 |
| KGRFG |  | 65.114 | -3.180 | 0.990 | \ | \ | \ | \ | \ | \ | \ | \ | \ |
| NGRFG |  | 15.523 | -0.877 | 1.000 | 14.030 | -0.953 | 0.972 | 11.111 | -0.958 | 0.780 | 15.234 | -1.174 | 0.973 |
| PGRFG |  | 10.421 | -0.420 | 0.982 | 20.408 | -0.851 | 0.905 | 21.719 | -0.967 | 0.898 | 24.871 | -1.134 | 0.913 |
| QGRFG |  | 20.180 | -0.870 | 0.931 | 26.212 | -1.210 | 0.964 | 26.188 | -1.297 | 0.877 | 27.150 | -1.385 | 0.836 |
| RGRFG |  | 3.751 | -0.336 | 0.953 | 16.348 | -0.862 | 0.684 | 17.212 | -0.961 | 0.689 | 17.761 | -1.030 | 0.757 |
| SGRFG |  | 23.276 | -1.043 | 0.988 | 25.197 | -1.196 | 0.936 | 25.088 | -1.276 | 0.863 | 26.118 | -1.364 | 0.844 |
| TGRFG |  | 19.471 | -0.872 | 0.990 | 22.695 | -1.071 | 0.982 | 24.511 | -1.233 | 0.930 | 25.508 | -1.334 | 0.828 |
| VGRFG |  | 9.652 | -0.288 | 0.980 | 9.784 | -0.357 | 0.984 | 18.587 | -0.753 | 0.900 | 21.074 | -0.894 | 0.923 |
| NPNPC |  | 11.440 | -0.624 | 0.976 | 11.326 | -0.676 | 0.929 | 6.768 | -0.646 | 0.884 | 5.255 | -0.688 | 0.785 |
| NPNPI |  | 6.776 | 0.233 | 0.960 | 6.271 | 0.135 | 0.953 | 4.871 | 0.054 | 0.917 | 5.980 | -0.084 | 0.980 |
| NPNPM |  | 8.293 | -0.158 | 0.993 | 7.217 | -0.229 | 0.998 | 7.105 | -0.321 | 1.000 | 11.947 | -0.568 | 0.980 |
| NPNPP |  | 6.654 | -0.358 | 0.966 | 13.329 | -0.683 | 0.921 | 14.978 | -0.863 | 0.914 | 21.495 | -1.205 | 0.794 |
| NPNPV |  | 5.036 | -0.128 | 1.000 | 5.522 | -0.222 | 0.984 | 5.384 | -0.295 | 0.954 | 11.815 | -0.593 | 0.958 |
| NPNPY |  | 11.457 | -0.212 | 0.986 | 9.868 | -0.268 | 0.998 | 9.354 | -0.342 | 1.000 | 10.354 | -0.444 | 0.995 |
| CPNPT |  | 7.008 | -0.427 | 0.897 | 14.396 | -0.770 | 0.947 | 18.144 | -0.981 | 0.880 | 18.584 | -1.072 | 1.000 |
| IPNPT |  | 4.941 | 0.051 | 0.954 | 5.448 | -0.081 | 1.000 | 5.032 | -0.163 | 0.970 | 6.394 | -0.285 | 0.960 |
| LPNPT |  | 6.337 | 0.152 | 0.954 | 5.524 | 0.039 | 0.946 | -2.799 | 0.197 | 0.275 | 5.642 | -0.211 | 0.996 |
| MPNPT |  | 6.612 | -0.112 | 0.982 | 6.674 | -0.215 | 0.992 | 6.471 | -0.299 | 0.985 | 13.348 | -0.618 | 0.970 |
| QPNPT |  | 6.639 | 0.012 | 1.000 | 6.081 | -0.079 | 0.999 | 4.554 | -0.142 | 0.992 | 6.010 | -0.274 | 0.948 |
| VPNPT |  | 5.795 | -0.245 | 0.950 | 6.183 | -0.339 | 0.946 | 12.471 | -0.659 | 0.933 | 14.742 | -0.802 | 0.948 |
| YPNPT |  | 14.168 | -0.215 | 0.989 | 11.817 | -0.285 | 0.998 | 8.832 | -0.322 | 0.999 | 6.927 | -0.325 | 0.740 |
| HGRFA | pH=4 | 4.931 | -0.256 | 0.816 | 4.090 | -0.296 | 0.682 | 3.548 | -0.346 | 0.613 | 3.137 | -0.396 | 0.539 |
| HGRFD |  | 7.929 | -0.394 | 0.983 | 7.399 | -0.441 | 0.978 | 7.062 | -0.498 | 0.961 | 6.832 | -0.555 | 0.948 |
| HGRFE |  | 7.409 | -0.379 | 0.976 | 7.007 | -0.434 | 0.935 | 6.664 | -0.489 | 0.891 | 6.530 | -0.552 | 0.784 |
| HGRFG |  | 12.064 | -0.641 | 0.831 | 9.219 | -0.604 | 0.714 | 6.629 | -0.594 | 0.565 | 2.069 | -0.547 | 0.142 |
| HGRFH |  | 0.664 | -0.426 | 0.010 | 0.677 | -0.477 | 0.011 | 0.031 | -0.511 | 0.000 | -0.031 | -0.566 | 0.000 |
| HGRFK |  | 2.088 | -0.443 | 0.128 | 2.265 | -0.494 | 0.174 | 2.113 | -0.539 | 0.189 | 2.833 | -0.634 | 0.564 |
| HGRFN |  | 11.160 | -0.876 | 0.996 | 9.438 | -0.946 | 0.976 | 1.223 | -0.800 | 0.179 | -0.032 | -0.799 | 0.000 |
| HGRFQ |  | -0.849 | -0.374 | 0.008 | -3.987 | -0.395 | 0.080 | -8.000 | -0.390 | 0.192 | -12.732 | -0.337 | 0.434 |
| HGRFR |  | 4.549 | -0.656 | 0.042 | -13.557 | -0.263 | 0.465 | -11.372 | -0.416 | 0.222 | -9.127 | -0.715 | 0.049 |
| HGRFS |  | 3.120 | -0.497 | 0.430 | -2.463 | -0.474 | 0.056 | -7.442 | -0.440 | 0.283 | -8.746 | -0.468 | 0.395 |
| HGRFT |  | 5.778 | -0.399 | 0.976 | 5.646 | -0.460 | 0.970 | 5.426 | -0.519 | 0.940 | 4.885 | -0.571 | 0.876 |
| AGRFG |  | 11.119 | -0.426 | 0.946 | 10.395 | -0.494 | 0.977 | 8.894 | -0.533 | 0.999 | 8.506 | -0.591 | 0.999 |
| DGRFG |  | 12.373 | -0.264 | 0.986 | 12.026 | -0.342 | 0.989 | 11.683 | -0.421 | 0.991 | 11.521 | -0.507 | 0.991 |
| EGRFG |  | 11.942 | -0.282 | 0.985 | 11.557 | -0.362 | 0.988 | 11.100 | -0.442 | 0.995 | 11.000 | -0.535 | 0.992 |
| GGRFG |  | 11.282 | -0.477 | 0.969 | 10.035 | -0.523 | 0.994 | 9.004 | -0.567 | 1.000 | 8.911 | -0.632 | 1.000 |
| KGRFG |  | 12.207 | -0.875 | 0.994 | 10.111 | -0.937 | 0.992 | 2.939 | -0.841 | 0.424 | -0.035 | -0.808 | 0.000 |
| NGRFG |  | 11.587 | -0.475 | 0.961 | 10.946 | -0.549 | 0.982 | 9.397 | -0.592 | 0.998 | 8.903 | -0.656 | 0.999 |
| PGRFG |  | 11.263 | -0.355 | 0.933 | 9.923 | -0.403 | 0.979 | 8.348 | -0.445 | 1.000 | 7.773 | -0.503 | 0.990 |
| QGRFG |  | 11.466 | -0.459 | 0.956 | 10.252 | -0.505 | 0.985 | 8.947 | -0.545 | 0.999 | 8.713 | -0.606 | 0.999 |
| RGRFG |  | 8.395 | -0.546 | 0.997 | 8.069 | -0.600 | 0.991 | 7.837 | -0.661 | 0.979 | 7.673 | -0.725 | 0.961 |
| SGRFG |  | 11.010 | -0.465 | 0.969 | 9.694 | -0.508 | 0.995 | 8.827 | -0.556 | 1.000 | 8.556 | -0.615 | 0.999 |
| TGRFG |  | 11.383 | -0.393 | 0.961 | 10.761 | -0.469 | 0.972 | 10.022 | -0.545 | 0.987 | 8.975 | -0.595 | 1.000 |
| VGRFG |  | 10.791 | -0.088 | 0.951 | 10.277 | -0.180 | 0.961 | 9.941 | -0.283 | 0.967 | 9.771 | -0.389 | 0.968 |
| NPNPC |  | 6.713 | -0.502 | 0.999 | 7.516 | -0.601 | 0.981 | 5.710 | -0.623 | 0.996 | 4.740 | -0.672 | 0.969 |
| NPNPI |  | 6.287 | 0.313 | 0.947 | 5.799 | 0.225 | 0.961 | 5.789 | 0.120 | 0.963 | 5.639 | 0.023 | 0.966 |
| NPNPM |  | 7.988 | -0.119 | 0.977 | 7.478 | -0.205 | 0.985 | 7.252 | -0.302 | 0.986 | 6.947 | -0.391 | 0.993 |
| NPNPP |  | 4.824 | -0.400 | 1.000 | 4.420 | -0.473 | 0.967 | 4.506 | -0.559 | 0.911 | 1.435 | -0.575 | 0.115 |
| NPNPV |  | 4.816 | -0.066 | 0.948 | 4.432 | -0.151 | 0.974 | 4.312 | -0.245 | 0.981 | 4.146 | -0.337 | 0.987 |
| NPNPY |  | 12.983 | -0.220 | 0.976 | 12.479 | -0.310 | 0.983 | 12.273 | -0.413 | 0.984 | 11.924 | -0.508 | 0.988 |
| CPNPT |  | 5.011 | -0.468 | 0.979 | 4.824 | -0.544 | 0.925 | 3.155 | -0.592 | 0.586 | 0.285 | -0.597 | 0.005 |
| IPNPT |  | 5.777 | 0.128 | 0.952 | 4.988 | 0.029 | 0.965 | 4.554 | -0.083 | 0.961 | 3.873 | -0.182 | 0.962 |
| LPNPT |  | 6.007 | 0.235 | 0.951 | 5.582 | 0.124 | 0.963 | 5.197 | 0.011 | 0.966 | 4.912 | -0.099 | 0.967 |
| MPNPT |  | 7.166 | -0.058 | 0.970 | 6.565 | -0.156 | 0.980 | 6.102 | -0.259 | 0.983 | 5.602 | -0.356 | 0.986 |
| QPNPT |  | 6.918 | -0.141 | 0.997 | 6.566 | -0.234 | 0.996 | 6.222 | -0.325 | 0.988 | 5.712 | -0.404 | 0.981 |
| VPNPT |  | 3.778 | -0.201 | 0.957 | 3.123 | -0.296 | 0.980 | 3.271 | -0.409 | 1.000 | 3.145 | -0.502 | 0.985 |
| YPNPT |  | 12.699 | -0.108 | 0.982 | 11.742 | -0.207 | 0.985 | 10.999 | -0.310 | 0.988 | 9.969 | -0.401 | 0.990 |
| HGRFA | pH=5 | 12.855 | -0.527 | 0.964 | 2.167 | -0.313 | 0.459 | 3.782 | -0.410 | 0.234 | 4.059 | -0.430 | 0.242 |
| HGRFD |  | 7.080 | -0.410 | 0.975 | 7.353 | -0.475 | 0.985 | 7.485 | -0.539 | 0.997 | 7.083 | -0.574 | 0.927 |
| HGRFE |  | 6.599 | -0.434 | 0.987 | 7.444 | -0.530 | 0.994 | 6.248 | -0.548 | 0.960 | 5.442 | -0.595 | 0.539 |
| HGRFG |  | 3.387 | -0.455 | 0.993 | 3.458 | -0.494 | 0.992 | 3.505 | -0.537 | 0.994 | 3.747 | -0.587 | 0.983 |
| HGRFH |  | 5.028 | -0.559 | 0.826 | 5.037 | -0.583 | 0.849 | 4.627 | -0.597 | 0.909 | 4.281 | -0.619 | 0.947 |
| HGRFK |  | 3.678 | -0.547 | 0.684 | 3.625 | -0.561 | 0.850 | 3.845 | -0.586 | 0.811 | 3.912 | -0.615 | 0.931 |
| HGRFN |  | 4.209 | -0.406 | 0.462 | 4.610 | -0.445 | 0.462 | 5.297 | -0.508 | 0.595 | 5.971 | -0.574 | 0.713 |
| HGRFQ |  | 4.389 | -0.382 | 0.478 | 4.990 | -0.433 | 0.503 | 5.490 | -0.489 | 0.543 | 5.934 | -0.551 | 0.673 |
| HGRFR |  | 4.537 | -0.538 | 0.717 | 4.790 | -0.561 | 0.739 | 4.988 | -0.593 | 0.810 | 4.784 | -0.611 | 0.948 |
| HGRFS |  | 4.011 | -0.371 | 0.453 | 4.448 | -0.416 | 0.508 | 5.225 | -0.481 | 0.615 | 5.880 | -0.547 | 0.711 |
| HGRFT |  | 3.170 | -0.307 | 0.306 | 3.592 | -0.334 | 0.344 | 4.252 | -0.379 | 0.390 | 5.038 | -0.439 | 0.492 |
| AGRFG |  | 11.007 | -0.453 | 0.998 | 6.608 | -0.388 | 0.843 | 6.986 | -0.452 | 0.895 | 7.481 | -0.521 | 0.912 |
| DGRFG |  | 11.318 | -0.252 | 0.987 | 11.044 | -0.326 | 0.985 | 10.446 | -0.391 | 0.988 | 10.396 | -0.469 | 0.987 |
| EGRFG |  | 11.387 | -0.239 | 0.988 | 11.322 | -0.323 | 0.981 | 10.256 | -0.372 | 0.983 | 10.472 | -0.457 | 0.984 |
| GGRFG |  | 7.760 | -0.395 | 0.972 | 7.326 | -0.438 | 0.961 | 7.647 | -0.503 | 0.973 | 7.814 | -0.565 | 0.972 |
| KGRFG |  | 7.312 | -0.565 | 0.930 | 7.464 | -0.623 | 0.935 | 7.627 | -0.682 | 0.946 | 7.896 | -0.746 | 0.939 |
| NGRFG |  | 11.370 | -0.417 | 0.984 | 9.121 | -0.431 | 0.939 | 9.156 | -0.503 | 0.953 | 8.913 | -0.565 | 0.941 |
| PGRFG |  | 15.900 | -0.486 | 1.000 | 11.121 | -0.451 | 0.993 | 6.833 | -0.395 | 0.794 | 7.084 | -0.455 | 0.850 |
| QGRFG |  | 11.370 | -0.464 | 0.965 | 7.625 | -0.420 | 0.824 | 7.715 | -0.479 | 0.875 | 8.025 | -0.542 | 0.881 |
| RGRFG |  | 6.158 | -0.468 | 0.887 | 7.529 | -0.555 | 0.957 | 7.579 | -0.610 | 0.960 | 7.698 | -0.667 | 0.952 |
| SGRFG |  | 10.525 | -0.455 | 0.961 | 8.220 | -0.458 | 0.937 | 7.652 | -0.501 | 0.940 | 7.425 | -0.552 | 0.921 |
| TGRFG |  | 13.738 | -0.440 | 0.993 | 8.843 | -0.381 | 0.910 | 8.981 | -0.455 | 0.931 | 9.041 | -0.526 | 0.924 |
| VGRFG |  | 7.909 | 0.069 | 0.991 | 15.875 | -0.329 | 0.998 | 12.761 | -0.396 | 1.000 | 10.003 | -0.429 | 0.922 |
| NPNPC |  | 5.360 | -0.504 | 0.997 | 3.753 | -0.515 | 0.999 | 3.649 | -0.588 | 0.996 | 3.143 | -0.643 | 0.996 |
| NPNPI |  | 4.429 | 0.257 | 0.979 | 4.291 | 0.166 | 0.979 | 4.186 | 0.076 | 0.973 | 4.089 | -0.008 | 0.972 |
| NPNPM |  | 6.334 | -0.152 | 0.990 | 5.959 | -0.237 | 0.999 | 5.665 | -0.325 | 0.994 | 5.452 | -0.407 | 0.993 |
| NPNPP |  | 2.709 | -0.531 | 0.964 | 2.191 | -0.616 | 0.826 | 1.437 | -0.689 | 0.819 | 0.559 | -0.746 | 0.408 |
| NPNPV |  | 3.004 | -0.102 | 0.995 | 3.002 | -0.193 | 0.957 | 2.680 | -0.280 | 0.999 | 2.494 | -0.357 | 0.997 |
| NPNPY |  | 13.665 | -0.307 | 1.000 | 10.652 | -0.328 | 0.993 | 10.372 | -0.421 | 0.998 | 11.947 | -0.573 | 0.998 |
| CPNPT |  | -3.525 | -0.281 | 0.662 | -0.405 | -0.487 | 0.972 | -0.579 | -0.572 | 0.990 | -1.054 | -0.637 | 0.846 |
| IPNPT |  | 3.082 | 0.124 | 0.949 | -3.420 | 0.249 | 0.384 | 1.816 | -0.060 | 0.939 | 1.735 | -0.162 | 0.985 |
| LPNPT |  | 3.722 | 0.215 | 0.981 | 3.393 | 0.111 | 0.975 | 2.922 | 0.013 | 0.974 | 2.646 | -0.083 | 0.904 |
| MPNPT |  | 4.352 | -0.052 | 0.985 | 4.162 | -0.154 | 0.993 | 3.814 | -0.247 | 0.985 | 3.320 | -0.325 | 0.998 |
| QPNPT |  | 3.746 | -0.155 | 0.797 | 3.935 | -0.221 | 0.814 | 10.032 | -0.660 | 0.077 | 10.488 | -0.735 | 0.081 |
| VPNPT |  | 1.452 | -0.198 | 0.982 | 1.933 | -0.312 | 0.568 | 1.872 | -0.415 | 0.588 | -1.987 | -0.412 | 1.000 |
| YPNPT |  | 10.096 | -0.095 | 0.989 | 9.232 | -0.189 | 0.985 | 8.689 | -0.285 | 0.993 | 7.120 | -0.352 | 0.943 |
| HGRFA | pH=6 | 6.689 | 0.113 | 0.992 | 5.808 | 0.040 | 0.994 | 4.242 | -0.010 | 0.860 | 2.085 | -0.052 | 0.454 |
| HGRFD |  | 13.024 | -0.406 | 0.993 | 12.100 | -0.482 | 0.993 | 11.224 | -0.562 | 0.998 | 11.084 | -0.673 | 0.984 |
| HGRFE |  | 12.421 | -0.394 | 0.994 | 11.315 | -0.467 | 0.991 | 10.494 | -0.546 | 0.993 | 9.803 | -0.643 | 0.955 |
| HGRFG |  | 7.970 | -0.041 | 1.000 | 5.731 | -0.073 | 0.909 | 3.441 | -0.116 | 0.838 | 4.629 | -0.334 | 0.069 |
| HGRFH |  | 4.840 | -0.009 | 0.954 | 1.928 | -0.039 | 0.534 | -1.549 | -0.061 | 0.407 | 1.725 | -0.335 | 0.086 |
| HGRFK |  | 6.609 | -0.397 | 0.996 | 4.518 | -0.437 | 0.986 | 3.130 | -0.497 | 0.990 | 5.230 | -0.680 | 0.820 |
| HGRFN |  | 11.196 | -0.289 | 0.996 | 11.870 | -0.420 | 0.980 | 12.866 | -0.561 | 0.958 | 14.200 | -0.729 | 0.997 |
| HGRFQ |  | 10.953 | -0.267 | 1.000 | 11.171 | -0.385 | 0.998 | 12.494 | -0.563 | 0.835 | 20.702 | -0.984 | 0.999 |
| HGRFR |  | 8.663 | -0.341 | 0.992 | 8.127 | -0.443 | 0.997 | 7.905 | -0.560 | 0.991 | 12.680 | -0.856 | 0.986 |
| HGRFS |  | 10.202 | -0.217 | 0.998 | 10.144 | -0.308 | 0.998 | 11.350 | -0.460 | 0.929 | 16.589 | -0.733 | 0.989 |
| HGRFT |  | 8.114 | 0.019 | 0.994 | 7.961 | -0.078 | 0.988 | 8.191 | -0.190 | 1.000 | 9.461 | -0.342 | 0.990 |
| AGRFG |  | 12.001 | -0.302 | 1.000 | 12.273 | -0.401 | 1.000 | 13.127 | -0.516 | 0.995 | 13.561 | -0.628 | 0.997 |
| DGRFG |  | 13.742 | -0.353 | 0.995 | 13.708 | -0.443 | 0.993 | 13.826 | -0.540 | 0.995 | 13.410 | -0.624 | 0.993 |
| EGRFG |  | 13.516 | -0.331 | 0.992 | 13.619 | -0.426 | 0.993 | 13.776 | -0.525 | 0.995 | 13.253 | -0.606 | 0.993 |
| GGRFG |  | 14.178 | -0.434 | 1.000 | 14.665 | -0.535 | 1.000 | 15.801 | -0.656 | 0.999 | 14.387 | -0.707 | 0.998 |
| KGRFG |  | 19.209 | -0.749 | 0.989 | 21.387 | -0.906 | 0.982 | 18.544 | -0.941 | 0.988 | 20.836 | -1.107 | 0.977 |
| NGRFG |  | 10.942 | -0.203 | 0.998 | 11.143 | -0.298 | 0.999 | 10.920 | -0.383 | 0.998 | 10.794 | -0.467 | 0.999 |
| PGRFG |  | 11.469 | -0.205 | 0.999 | 11.150 | -0.293 | 1.000 | 11.299 | -0.392 | 0.999 | 11.974 | -0.516 | 1.000 |
| QGRFG |  | 13.377 | -0.384 | 1.000 | 13.570 | -0.473 | 1.000 | 14.383 | -0.583 | 0.998 | 14.683 | -0.687 | 1.000 |
| RGRFG |  | 12.582 | -0.497 | 1.000 | 13.282 | -0.621 | 0.996 | 14.125 | -0.754 | 0.978 | 17.439 | -0.982 | 0.999 |
| SGRFG |  | 10.704 | -0.251 | 1.000 | 10.979 | -0.345 | 0.999 | 11.133 | -0.438 | 0.998 | 11.793 | -0.550 | 1.000 |
| TGRFG |  | 9.222 | -0.092 | 1.000 | 9.378 | -0.184 | 0.999 | 9.065 | -0.266 | 0.998 | 9.056 | -0.352 | 0.996 |
| VGRFG |  | 5.681 | 0.336 | 0.948 | 5.677 | 0.240 | 0.953 | 5.278 | 0.154 | 0.937 | 4.775 | 0.078 | 0.875 |
| NPNPC |  | 7.955 | -0.651 | 0.999 | 7.903 | -0.729 | 0.998 | 8.515 | -0.856 | 0.986 | 7.765 | -0.931 | 0.976 |
| NPNPI |  | 6.569 | 0.165 | 0.990 | 6.679 | 0.058 | 0.987 | 6.555 | -0.044 | 0.988 | 6.560 | -0.146 | 0.990 |
| NPNPM |  | 8.241 | -0.242 | 0.994 | 8.505 | -0.358 | 0.997 | 8.444 | -0.465 | 0.998 | 8.316 | -0.569 | 0.999 |
| NPNPP |  | 3.973 | -0.689 | 0.997 | 3.547 | -0.795 | 0.999 | 2.786 | -0.904 | 0.910 | 4.363 | -1.109 | 0.243 |
| NPNPV |  | 5.243 | -0.227 | 0.998 | 5.441 | -0.336 | 0.999 | 5.468 | -0.442 | 1.000 | 5.380 | -0.541 | 1.000 |
| NPNPY |  | 13.938 | -0.379 | 0.995 | 13.942 | -0.490 | 0.991 | 13.925 | -0.603 | 0.993 | 13.809 | -0.709 | 0.994 |
| CPNPT |  | -0.038 | -0.376 | 0.003 | -1.120 | -0.455 | 0.970 | -1.050 | -0.610 | 0.185 | 2.006 | -0.811 | 0.427 |
| IPNPT |  | 1.710 | 0.209 | 0.925 | 1.105 | 0.112 | 0.845 | 0.036 | 0.028 | 0.003 | -0.585 | -0.061 | 0.286 |
| LPNPT |  | 3.308 | 0.258 | 0.999 | 2.806 | 0.157 | 1.000 | 2.163 | 0.062 | 0.983 | 1.916 | -0.041 | 0.895 |
| MPNPT |  | 3.620 | 0.007 | 0.997 | 3.156 | -0.092 | 0.995 | 2.103 | -0.172 | 0.945 | 2.194 | -0.280 | 0.868 |
| QPNPT |  | 8.144 | -0.605 | 0.990 | 11.385 | -0.777 | 0.984 | 6.748 | -0.748 | 0.966 | -2.973 | -0.579 | 0.125 |
| VPNPT |  | -1.017 | -0.147 | 0.781 | -1.993 | -0.235 | 0.881 | -3.082 | -0.323 | 0.808 | -3.698 | -0.420 | 0.705 |
| YPNPT |  | 11.048 | -0.119 | 0.996 | 10.545 | -0.225 | 0.993 | 9.780 | -0.325 | 0.997 | 9.291 | -0.427 | 0.999 |
| HGRFA | pH=7 | 13.408 | 0.372 | 1.000 | 11.127 | 0.336 | 0.996 | 11.619 | 0.235 | 1.000 | 11.654 | 0.153 | 0.999 |
| HGRFD |  | 19.535 | -0.502 | 1.000 | 15.928 | -0.520 | 0.998 | 15.388 | -0.603 | 0.999 | 14.507 | -0.665 | 1.000 |
| HGRFE |  | 18.829 | -0.488 | 1.000 | 15.384 | -0.513 | 0.997 | 14.573 | -0.588 | 1.000 | 14.562 | -0.687 | 1.000 |
| HGRFG |  | 13.698 | 0.262 | 1.000 | 12.544 | 0.201 | 1.000 | 12.621 | 0.115 | 1.000 | 13.957 | -0.011 | 0.998 |
| HGRFH |  | 17.368 | 0.320 | 1.000 | 15.687 | 0.265 | 1.000 | 16.034 | 0.163 | 1.000 | 16.369 | 0.070 | 0.996 |
| HGRFK |  | 9.353 | 0.514 | 0.712 | 8.578 | 0.440 | 0.859 | 10.695 | 0.327 | 0.860 | 11.646 | 0.252 | 0.869 |
| HGRFN |  | 14.174 | 0.147 | 0.999 | 11.168 | 0.137 | 1.000 | 11.921 | 0.034 | 0.993 | 12.331 | -0.047 | 0.977 |
| HGRFQ |  | 14.638 | 0.169 | 0.999 | 12.257 | 0.140 | 0.997 | 12.295 | 0.054 | 0.991 | 12.738 | -0.034 | 0.974 |
| HGRFR |  | 29.591 | -0.066 | 0.904 | 10.860 | 0.521 | 0.969 | 12.119 | 0.433 | 0.971 | 12.657 | 0.363 | 0.929 |
| HGRFS |  | 13.914 | 0.187 | 1.000 | 12.008 | 0.150 | 0.999 | 11.831 | 0.074 | 0.994 | 12.184 | -0.010 | 0.983 |
| HGRFT |  | 13.789 | 0.321 | 1.000 | 11.802 | 0.282 | 1.000 | 11.915 | 0.190 | 0.998 | 12.158 | 0.101 | 0.993 |
| AGRFG |  | 9.698 | 0.296 | 0.999 | 8.488 | 0.254 | 0.991 | 8.624 | 0.187 | 1.000 | 9.278 | 0.104 | 0.999 |
| DGRFG |  | 16.398 | -0.449 | 0.989 | 15.181 | -0.500 | 0.994 | 15.389 | -0.604 | 0.997 | 15.201 | -0.690 | 1.000 |
| EGRFG |  | 15.962 | -0.381 | 0.998 | 14.997 | -0.447 | 0.993 | 14.995 | -0.535 | 0.997 | 15.202 | -0.631 | 0.998 |
| GGRFG |  | 12.264 | 0.132 | 1.000 | 11.441 | 0.087 | 1.000 | 11.677 | 0.019 | 1.000 | 11.369 | -0.025 | 0.993 |
| KGRFG |  | 11.484 | 0.515 | 1.000 | 10.527 | 0.498 | 0.997 | 10.728 | 0.461 | 0.944 | 10.715 | 0.432 | 0.882 |
| NGRFG |  | 11.649 | 0.035 | 1.000 | 10.751 | -0.022 | 0.999 | 10.358 | -0.088 | 1.000 | 10.201 | -0.156 | 0.998 |
| PGRFG |  | 6.291 | 0.649 | 0.998 | 4.438 | 0.628 | 0.999 | 4.641 | 0.561 | 0.985 | 5.066 | 0.492 | 0.955 |
| QGRFG |  | 10.620 | 0.211 | 0.997 | 7.139 | 0.233 | 0.945 | 5.198 | 0.220 | 0.855 | 2.897 | 0.226 | 0.663 |
| RGRFG |  | 10.986 | 0.198 | 1.000 | 9.732 | 0.168 | 0.998 | 10.060 | 0.096 | 1.000 | 9.907 | 0.046 | 0.996 |
| SGRFG |  | 11.812 | 0.062 | 1.000 | 10.669 | 0.017 | 0.999 | 10.591 | -0.055 | 1.000 | 8.460 | 0.007 | 0.345 |
| TGRFG |  | 10.916 | 0.203 | 0.994 | 10.697 | 0.127 | 0.995 | 9.999 | 0.061 | 0.997 | 9.827 | -0.005 | 1.000 |
| VGRFG |  | 6.641 | 0.834 | 0.978 | 6.735 | 0.735 | 0.985 | 5.959 | 0.661 | 0.980 | 6.603 | 0.555 | 0.992 |
| NPNPC |  | 82.716 | -3.467 | 0.641 | \ | \ | \ | \ | \ | \ | \ | \ | \ |
| NPNPI |  | 8.559 | -0.107 | 0.988 | 10.270 | -0.269 | 0.999 | 8.847 | -0.345 | 0.997 | 8.723 | -0.455 | 0.999 |
| NPNPM |  | 10.429 | -0.458 | 0.999 | 10.430 | -0.568 | 0.996 | 8.422 | -0.617 | 0.955 | 6.292 | -0.642 | 0.848 |
| NPNPP |  | 7.955 | -0.748 | 0.855 | 11.032 | -0.954 | 0.788 | 4.626 | -0.861 | 0.504 | 6.469 | -1.018 | 0.543 |
| NPNPV |  | 6.930 | -0.437 | 0.999 | 6.953 | -0.538 | 0.947 | 4.812 | -0.563 | 0.795 | 10.017 | -0.830 | 0.919 |
| NPNPY |  | 14.552 | -0.552 | 0.998 | 14.654 | -0.665 | 1.000 | 11.463 | -0.677 | 0.951 | 10.031 | -0.732 | 0.939 |
| CPNPT |  | 2.830 | -0.511 | 0.639 | 14.594 | -0.940 | 0.993 | 15.217 | -1.086 | 0.784 | 4.696 | -0.934 | 0.686 |
| IPNPT |  | 2.893 | 0.310 | 0.940 | 3.199 | 0.185 | 0.956 | 3.112 | 0.074 | 0.953 | 3.247 | -0.032 | 0.971 |
| LPNPT |  | 3.887 | 0.345 | 0.971 | 3.641 | 0.240 | 0.969 | 4.140 | 0.109 | 0.955 | 4.172 | 0.001 | 0.979 |
| MPNPT |  | 8.340 | -0.086 | 0.991 | 7.721 | -0.185 | 0.991 | 7.647 | -0.298 | 0.995 | 7.374 | -0.397 | 0.999 |
| QPNPT |  | 7.051 | -0.700 | 0.899 | 7.094 | -0.801 | 0.676 | 3.813 | -0.788 | 0.542 | 3.077 | -0.892 | 0.378 |
| VPNPT |  | 1.891 | -0.026 | 0.972 | 2.428 | -0.143 | 1.000 | 2.424 | -0.243 | 0.989 | 2.421 | -0.335 | 0.931 |
| YPNPT |  | 13.639 | -0.184 | 0.947 | 15.346 | -0.366 | 0.998 | 13.081 | -0.429 | 0.994 | 12.474 | -0.525 | 1.000 |
| HGRFA | pH=8 | 10.240 | 0.379 | 0.998 | 9.910 | 0.276 | 0.996 | 9.290 | 0.189 | 0.993 | 8.938 | 0.104 | 0.982 |
| HGRFD |  | 10.881 | -0.209 | 0.999 | 10.567 | -0.310 | 0.960 | 10.078 | -0.405 | 0.969 | 9.440 | -0.481 | 0.963 |
| HGRFE |  | 10.569 | -0.194 | 0.966 | 9.289 | -0.268 | 0.987 | 7.920 | -0.330 | 0.988 | 7.730 | -0.425 | 0.983 |
| HGRFG |  | 10.567 | 0.261 | 0.994 | 10.102 | 0.167 | 0.994 | 9.248 | 0.089 | 0.986 | 8.742 | 0.008 | 0.964 |
| HGRFH |  | 14.118 | 0.294 | 1.000 | 12.931 | 0.207 | 0.999 | 12.249 | 0.110 | 0.998 | 11.615 | 0.023 | 0.986 |
| HGRFK |  | -6.135 | 0.641 | 0.188 | -7.145 | 0.585 | 0.214 | -6.757 | 0.489 | 0.209 | -8.063 | 0.446 | 0.277 |
| HGRFN |  | 9.699 | 0.166 | 0.973 | 8.853 | 0.085 | 0.947 | 8.080 | 0.006 | 0.924 | 7.489 | -0.068 | 0.857 |
| HGRFQ |  | 10.432 | 0.168 | 0.985 | 9.731 | 0.076 | 0.976 | 8.833 | -0.004 | 0.956 | 8.100 | -0.079 | 0.905 |
| HGRFR |  | 5.328 | 0.373 | 0.440 | 3.629 | 0.328 | 0.210 | 1.582 | 0.299 | 0.037 | -0.235 | 0.277 | 0.001 |
| HGRFS |  | 9.851 | 0.193 | 0.983 | 8.980 | 0.115 | 0.969 | 8.261 | 0.035 | 0.947 | 7.618 | -0.037 | 0.892 |
| HGRFT |  | 10.276 | 0.327 | 0.992 | 9.779 | 0.231 | 0.991 | 9.140 | 0.144 | 0.983 | 8.655 | 0.062 | 0.958 |
| AGRFG |  | 6.774 | 0.252 | 0.993 | 6.856 | 0.155 | 0.994 | 6.648 | 0.070 | 0.988 | 6.667 | -0.015 | 0.975 |
| DGRFG |  | 12.817 | -0.305 | 0.943 | 12.987 | -0.426 | 0.924 | 13.086 | -0.537 | 0.927 | 13.146 | -0.640 | 0.910 |
| EGRFG |  | 12.307 | -0.304 | 0.950 | 12.615 | -0.424 | 0.937 | 12.568 | -0.529 | 0.936 | 11.904 | -0.615 | 0.926 |
| GGRFG |  | 7.536 | 0.075 | 0.981 | 7.584 | -0.015 | 0.986 | 6.962 | -0.081 | 0.975 | 7.843 | -0.197 | 0.997 |
| KGRFG |  | -11.794 | 0.775 | 0.322 | -13.274 | 0.756 | 0.361 | -14.616 | 0.735 | 0.387 | -15.330 | 0.701 | 0.395 |
| NGRFG |  | 8.632 | 0.018 | 0.939 | 8.248 | -0.066 | 0.924 | 7.513 | -0.135 | 0.884 | 6.539 | -0.190 | 0.796 |
| PGRFG |  | 3.637 | 0.749 | 0.868 | 3.683 | 0.646 | 0.933 | -7.529 | 0.829 | 0.855 | 3.737 | 0.460 | 0.873 |
| QGRFG |  | 5.368 | 0.202 | 0.821 | 5.708 | 0.105 | 0.874 | 5.982 | 0.010 | 0.913 | 5.315 | -0.045 | 0.937 |
| RGRFG |  | 7.303 | 0.127 | 0.914 | 6.632 | 0.067 | 0.885 | 5.996 | 0.008 | 0.866 | 5.370 | -0.047 | 0.871 |
| SGRFG |  | 9.060 | 0.012 | 0.997 | 8.885 | -0.076 | 0.998 | 8.200 | -0.142 | 0.990 | 7.696 | -0.209 | 0.977 |
| TGRFG |  | 8.833 | 0.163 | 1.000 | 8.711 | 0.069 | 1.000 | 8.377 | -0.013 | 0.998 | 8.094 | -0.091 | 0.991 |
| VGRFG |  | 8.674 | 0.793 | 0.993 | 8.694 | 0.673 | 0.991 | 8.388 | 0.569 | 0.994 | 8.294 | 0.465 | 0.998 |
| NPNPC |  | 5.444 | -0.557 | 0.893 | 2.963 | -0.577 | 0.981 | 0.779 | -0.631 | 0.200 | -2.752 | -0.632 | 0.350 |
| NPNPI |  | 8.148 | 0.042 | 0.704 | 6.830 | -0.039 | 0.692 | 5.935 | -0.130 | 0.670 | 5.567 | -0.230 | 0.663 |
| NPNPM |  | 7.934 | -0.242 | 0.882 | 8.083 | -0.382 | 0.861 | 7.142 | -0.487 | 0.731 | 5.980 | -0.579 | 0.731 |
| NPNPP |  | 1.049 | -0.526 | 0.800 | -4.064 | -0.552 | 0.947 | -7.930 | -0.552 | 0.632 | -8.634 | -0.658 | 0.571 |
| NPNPV |  | 4.282 | -0.238 | 0.735 | 3.712 | -0.352 | 0.554 | 2.749 | -0.454 | 0.416 | 1.446 | -0.534 | 0.190 |
| NPNPY |  | 12.595 | -0.371 | 0.931 | 12.985 | -0.521 | 0.924 | 13.276 | -0.662 | 0.902 | 11.270 | -0.734 | 0.886 |
| CPNPT |  | 3.998 | -0.257 | 0.155 | 8.847 | -0.607 | 0.458 | 7.599 | -0.740 | 0.250 | 10.014 | -0.930 | 0.408 |
| IPNPT |  | 3.080 | 0.580 | 0.357 | 3.682 | 0.419 | 0.425 | 3.668 | 0.288 | 0.379 | 3.725 | 0.170 | 0.426 |
| LPNPT |  | 2.946 | 0.619 | 0.310 | 3.366 | 0.468 | 0.356 | 11.963 | 0.023 | 0.515 | 3.830 | 0.208 | 0.416 |
| MPNPT |  | 6.352 | 0.170 | 0.736 | 6.190 | 0.037 | 0.723 | 6.244 | -0.095 | 0.674 | 5.893 | -0.204 | 0.663 |
| QPNPT |  | -0.151 | -0.411 | 0.037 | -2.620 | -0.500 | 0.937 | -6.437 | -0.513 | 0.886 | -7.938 | -0.559 | 0.754 |
| VPNPT |  | 2.149 | 0.196 | 0.280 | 1.834 | 0.072 | 0.225 | 1.814 | -0.057 | 0.185 | 1.264 | -0.158 | 0.105 |
| YPNPT |  | 11.598 | -0.002 | 0.892 | 10.890 | -0.122 | 0.894 | 10.986 | -0.266 | 0.874 | 10.813 | -0.388 | 0.855 |

**Supplementary Table 2.** The fitting results of $logk$ to φ or $P_{m}^{N}$

| Pentapeptide sequence | pH | $logk=-S\varphi+logk_{W}$ | | | | | | | | |
| --- | --- | --- | --- | --- | --- | --- | --- | --- | --- | --- |
|  |  | 45℃ | | | 35℃ | | | 25℃ | | |
|  |  | -S | $logk_{W}$ | R^2^ | -S | $logk_{W}$ | R^2^ | -S | $logk_{W}$ | R^2^ |
| APNPT | pH=2 | -8.266 | 1.464 | 0.996 | -8.307 | 1.543 | 0.998 | -8.670 | 1.677 | 0.997 |
| DPNPT |  | -8.074 | 1.401 | 0.996 | -8.156 | 1.497 | 0.998 | -8.595 | 1.660 | 0.997 |
| EPNPT |  | -8.716 | 1.565 | 0.997 | -8.820 | 1.672 | 0.998 | -9.325 | 1.850 | 0.997 |
| GPNPT |  | -7.565 | 1.269 | 0.995 | -7.641 | 1.357 | 0.998 | -8.010 | 1.497 | 0.997 |
| HPNPT |  | -9.154 | 1.518 | 0.997 | -9.413 | 1.680 | 0.998 | -9.986 | 1.872 | 0.996 |
| KPNPT |  | -7.770 | 1.186 | 0.994 | -7.738 | 1.278 | 0.998 | -8.081 | 1.394 | 0.996 |
| NPNPT |  | -7.954 | 1.314 | 0.996 | -8.059 | 1.411 | 0.998 | -8.544 | 1.579 | 0.997 |
| PPNPT |  | -9.343 | 1.752 | 0.997 | -9.477 | 1.853 | 0.998 | -9.965 | 2.017 | 0.997 |
| RPNPT |  | -9.194 | 1.564 | 0.996 | -9.450 | 1.731 | 0.997 | -9.976 | 1.916 | 0.997 |
| SPNPT |  | -7.771 | 1.313 | 0.997 | -7.931 | 1.412 | 0.998 | -8.313 | 1.555 | 0.997 |
| TPNPT |  | -8.856 | 1.639 | 0.998 | -8.987 | 1.740 | 0.998 | -9.358 | 1.887 | 0.997 |
| NPNPA |  | -7.722 | 1.294 | 0.996 | -7.756 | 1.371 | 0.996 | -8.264 | 1.522 | 0.994 |
| NPNPD |  | -7.280 | 1.042 | 0.995 | -7.251 | 1.119 | 0.997 | -7.738 | 1.286 | 0.995 |
| NPNPE |  | -8.035 | 1.300 | 0.995 | -8.012 | 1.393 | 0.997 | -8.643 | 1.593 | 0.995 |
| NPNPG |  | -7.150 | 0.999 | 0.995 | -7.115 | 1.063 | 0.997 | -7.589 | 1.212 | 0.995 |
| NPNPH |  | -7.728 | 1.033 | 0.993 | -7.673 | 1.132 | 0.997 | -8.206 | 1.297 | 0.995 |
| NPNPK |  | -7.667 | 0.987 | 0.994 | -7.593 | 1.069 | 0.997 | -8.061 | 1.204 | 0.994 |
| NPNPN |  | -7.031 | 0.814 | 0.996 | -7.034 | 0.877 | 0.998 | -7.550 | 1.027 | 0.995 |
| NPNPQ |  | -7.480 | 0.987 | 0.996 | -7.537 | 1.064 | 0.998 | -8.103 | 1.232 | 0.995 |
| NPNPR |  | -8.488 | 1.246 | 0.995 | -8.569 | 1.378 | 0.997 | -9.180 | 1.570 | 0.995 |
| NPNPS |  | -7.219 | 0.969 | 0.996 | -7.285 | 1.051 | 0.998 | -7.730 | 1.207 | 0.997 |
| HGRFA |  | -5.852 | 1.787 | 1.000 | -6.192 | 2.063 | 1.000 | -7.005 | 2.359 | 0.997 |
| HGRFD |  | -5.757 | 1.678 | 1.000 | -6.136 | 1.983 | 1.000 | -7.053 | 2.340 | 0.998 |
| HGRFE |  | -5.870 | 1.711 | 1.000 | -6.291 | 2.011 | 1.000 | -7.051 | 2.318 | 0.998 |
| HGRFG |  | -4.900 | 1.522 | 0.781 | -5.940 | 1.927 | 1.000 | -6.785 | 2.238 | 0.999 |
| HGRFH |  | -5.884 | 1.522 | 1.000 | -6.284 | 1.831 | 1.000 | -7.354 | 2.187 | 0.996 |
| HGRFK |  | -5.545 | 1.398 | 1.000 | -6.110 | 1.738 | 1.000 | -7.160 | 2.072 | 0.996 |
| HGRFN |  | -5.484 | 1.434 | 1.000 | -5.727 | 1.680 | 1.000 | -6.627 | 2.005 | 0.998 |
| HGRFQ |  | -5.670 | 1.546 | 1.000 | -6.005 | 1.815 | 1.000 | -6.813 | 2.122 | 0.998 |
| HGRFR |  | -6.033 | 1.600 | 1.000 | -6.701 | 1.974 | 1.000 | -7.729 | 2.329 | 0.996 |
| HGRFS |  | -5.621 | 1.568 | 1.000 | -5.900 | 1.831 | 1.000 | -6.819 | 2.164 | 0.998 |
| HGRFT |  | -6.017 | 1.808 | 1.000 | -6.317 | 2.084 | 1.000 | -7.241 | 2.423 | 0.997 |
| AGRFG |  | -5.180 | 1.593 | 1.000 | -5.269 | 1.773 | 1.000 | -5.864 | 2.023 | 0.999 |
| DGRFG |  | -5.145 | 1.558 | 1.000 | -5.259 | 1.743 | 1.000 | -5.852 | 2.003 | 0.999 |
| EGRFG |  | -5.338 | 1.642 | 1.000 | -5.466 | 1.836 | 1.000 | -6.094 | 2.104 | 0.999 |
| GGRFG |  | -4.961 | 1.453 | 1.000 | -5.169 | 1.663 | 1.000 | -5.688 | 1.892 | 0.999 |
| KGRFG |  | -5.531 | 1.623 | 1.000 | -5.741 | 1.860 | 1.000 | -6.492 | 2.146 | 0.998 |
| NGRFG |  | -5.169 | 1.537 | 1.000 | -5.283 | 1.723 | 1.000 | -5.851 | 1.972 | 0.999 |
| PGRFG |  | -5.616 | 1.778 | 1.000 | -5.769 | 1.975 | 1.000 | -6.372 | 2.233 | 0.999 |
| QGRFG |  | -5.061 | 1.575 | 1.000 | -5.169 | 1.763 | 1.000 | -5.787 | 2.022 | 0.999 |
| RGRFG |  | -5.789 | 1.734 | 1.000 | -6.171 | 2.021 | 1.000 | -7.017 | 2.341 | 0.998 |
| SGRFG |  | -5.122 | 1.537 | 1.000 | -5.230 | 1.722 | 1.000 | -5.812 | 1.969 | 0.999 |
| TGRFG |  | -4.519 | 1.402 | 0.715 | -5.425 | 1.812 | 1.000 | -6.028 | 2.062 | 0.999 |
| VGRFG |  | -6.122 | 2.081 | 1.000 | -6.210 | 2.274 | 0.999 | -6.531 | 2.460 | 0.997 |
| NPNPC |  | -8.739 | 2.289 | 1.000 | -9.017 | 2.525 | 0.997 | -10.120 | 2.941 | 0.999 |
| NPNPI |  | -5.620 | 2.038 | 0.996 | -5.537 | 2.104 | 0.996 | -6.011 | 2.297 | 1.000 |
| NPNPM |  | -5.366 | 1.616 | 1.000 | -5.254 | 1.692 | 0.997 | -5.752 | 1.904 | 1.000 |
| NPNPP |  | -5.505 | 1.330 | 1.000 | -5.424 | 1.391 | 0.995 | -6.025 | 1.607 | 0.999 |
| NPNPV |  | -5.217 | 1.555 | 1.000 | -5.029 | 1.592 | 0.996 | -5.448 | 1.760 | 1.000 |
| NPNPY |  | -5.661 | 1.687 | 1.000 | -5.609 | 1.806 | 0.999 | -6.078 | 2.056 | 1.000 |
| CPNPT |  | -5.082 | 1.154 | 1.000 | -5.007 | 1.212 | 0.996 | -5.504 | 1.399 | 0.999 |
| IPNPT |  | -6.876 | 2.128 | 1.000 | -6.763 | 2.202 | 0.996 | -7.337 | 2.415 | 1.000 |
| LPNPT |  | -7.001 | 2.265 | 1.000 | -6.859 | 2.337 | 0.996 | -7.396 | 2.547 | 1.000 |
| MPNPT |  | -6.372 | 1.875 | 1.000 | -6.323 | 1.969 | 0.996 | -6.879 | 2.194 | 1.000 |
| QPNPT |  | -5.581 | 1.301 | 1.000 | -5.505 | 1.334 | 0.996 | -5.964 | 1.521 | 0.999 |
| VPNPT |  | -6.068 | 1.616 | 1.000 | -5.993 | 1.678 | 0.995 | -6.576 | 1.882 | 1.000 |
| YPNPT |  | -6.985 | 2.018 | 0.999 | -7.048 | 2.167 | 0.997 | -7.731 | 2.474 | 1.000 |
| APNPT | pH=3 | -5.796 | 0.656 | 0.980 | -6.794 | 0.801 | 0.983 | -6.289 | 0.812 | 0.960 |
| DPNPT |  | -6.875 | 0.861 | 0.996 | -7.603 | 0.995 | 0.997 | -7.826 | 1.113 | 0.992 |
| EPNPT |  | -7.708 | 0.946 | 0.995 | -8.494 | 1.099 | 0.997 | -9.007 | 1.242 | 0.991 |
| GPNPT |  | -4.496 | 0.447 | 0.996 | -5.464 | 0.580 | 0.984 | -5.000 | 0.612 | 0.978 |
| HPNPT |  | -13.107 | 0.895 | 0.955 | -12.847 | 0.963 | 0.969 | -4.654 | 0.368 | 0.995 |
| KPNPT |  | -17.602 | 1.264 | 0.918 | -21.468 | 1.609 | 0.932 | -3.489 | 0.161 | 0.993 |
| NPNPT |  | -7.207 | 0.680 | 0.994 | -8.107 | 0.835 | 0.997 | -7.531 | 0.860 | 0.972 |
| PPNPT |  | -8.286 | 1.087 | 0.995 | -9.145 | 1.244 | 0.997 | -9.536 | 1.354 | 0.990 |
| RPNPT |  | -14.504 | 0.999 | 0.988 | -15.055 | 1.173 | 0.957 | -4.337 | 0.345 | 0.996 |
| SPNPT |  | -7.309 | 0.741 | 0.996 | -7.344 | 0.807 | 0.993 | -7.259 | 0.880 | 0.976 |
| TPNPT |  | -7.124 | 0.893 | 0.983 | -8.168 | 1.061 | 0.991 | -8.041 | 1.111 | 0.969 |
| NPNPA |  | -4.993 | 0.513 | 0.985 | -6.090 | 0.665 | 0.975 | -5.719 | 0.698 | 0.963 |
| NPNPD |  | -7.576 | 0.603 | 0.994 | -7.822 | 0.693 | 0.992 | -5.961 | 0.620 | 0.975 |
| NPNPE |  | -7.580 | 0.748 | 0.996 | -8.218 | 0.891 | 0.997 | -7.756 | 0.957 | 0.980 |
| NPNPG |  | -7.959 | 0.594 | 0.980 | -8.442 | 0.686 | 0.994 | -5.262 | 0.496 | 0.980 |
| NPNPH |  | -14.222 | 0.655 | 0.997 | -16.986 | 0.980 | 0.955 | -7.924 | 0.400 | 0.888 |
| NPNPK |  | \ | \ | \ | \ | \ | \ | -18.893 | 1.201 | 0.883 |
| NPNPN |  | -6.741 | 0.328 | 0.945 | -8.283 | 0.509 | 0.987 | -8.391 | 0.644 | 0.987 |
| NPNPQ |  | -8.384 | 0.613 | 0.970 | -8.416 | 0.674 | 0.951 | -5.299 | 0.484 | 0.986 |
| NPNPR |  | -12.846 | 0.635 | 0.963 | -15.765 | 1.063 | 0.967 | -4.495 | 0.283 | 0.994 |
| NPNPS |  | -7.977 | 0.564 | 0.976 | -8.755 | 0.677 | 0.993 | -5.250 | 0.468 | 0.974 |
| HGRFA |  | -3.556 | -0.387 | 0.961 | -3.550 | 0.384 | 0.999 | -2.646 | 0.383 | 0.992 |
| HGRFD |  | -3.003 | 0.321 | 0.999 | -2.930 | 0.345 | 1.000 | -2.503 | 0.437 | 0.992 |
| HGRFE |  | -3.137 | 0.290 | 0.995 | -3.121 | 0.326 | 1.000 | -2.536 | 0.382 | 0.991 |
| HGRFG |  | -8.201 | 1.128 | 0.739 | -2.845 | -0.044 | 0.998 | -2.752 | 0.368 | 0.987 |
| HGRFH |  | -1.347 | -0.231 | 0.987 | -2.017 | -0.098 | 0.953 | -2.232 | 0.178 | 0.991 |
| HGRFK |  | -1.980 | -0.095 | 0.994 | -3.288 | 0.219 | 0.970 | -1.993 | 0.163 | 0.993 |
| HGRFN |  | \ | \ | \ | \ | \ | \ | -2.429 | 0.172 | 0.968 |
| HGRFQ |  | \ | \ | \ | \ | \ | \ | -2.936 | 0.336 | 0.988 |
| HGRFR |  | -2.208 | -0.030 | 0.997 | -3.387 | 0.262 | 0.985 | -2.343 | 0.269 | 0.992 |
| HGRFS |  | \ | \ | \ | \ | \ | \ | -2.349 | 0.156 | 0.971 |
| HGRFT |  | \ | \ | \ | \ | \ | \ | -2.967 | 0.368 | 0.989 |
| AGRFG |  | -4.054 | 0.364 | 0.957 | -3.462 | 0.493 | 0.999 | -2.745 | 0.489 | 0.991 |
| DGRFG |  | -5.376 | 0.927 | 0.996 | -5.327 | 1.013 | 0.979 | -3.609 | 0.778 | 0.988 |
| EGRFG |  | -5.153 | 0.690 | 0.999 | -6.883 | 1.173 | 0.994 | -3.341 | 0.621 | 0.989 |
| GGRFG |  | -3.157 | 0.137 | 0.987 | -7.755 | 1.388 | 0.830 | -2.753 | 0.479 | 0.992 |
| KGRFG |  | \ | \ | \ | \ | \ | \ | -6.334 | 0.713 | 0.902 |
| NGRFG |  | -4.519 | 0.377 | 0.958 | -5.607 | 0.671 | 0.974 | -5.024 | 0.734 | 0.980 |
| PGRFG |  | -7.264 | 1.224 | 0.928 | -3.479 | 0.562 | 0.999 | -2.882 | 0.576 | 0.992 |
| QGRFG |  | -3.947 | 0.307 | 0.939 | -8.114 | 1.333 | 0.932 | -2.930 | 0.501 | 0.991 |
| RGRFG |  | -7.064 | 1.078 | 0.835 | -3.333 | 0.449 | 0.999 | -2.839 | 0.376 | 0.992 |
| SGRFG |  | -3.314 | 0.146 | 0.991 | -5.537 | 0.688 | 0.981 | -2.999 | 0.494 | 0.991 |
| TGRFG |  | -4.446 | 0.446 | 0.966 | -6.584 | 0.985 | 0.998 | -3.156 | 0.542 | 0.989 |
| VGRFG |  | -7.277 | 1.413 | 0.946 | -3.394 | 0.654 | 0.999 | -3.011 | 0.702 | 0.990 |
| NPNPC |  | -3.311 | 0.313 | 0.989 | -4.232 | 0.537 | 0.996 | -5.398 | 0.940 | 0.969 |
| NPNPI |  | -5.522 | 1.479 | 1.000 | -5.772 | 1.596 | 1.000 | -5.881 | 1.672 | 0.994 |
| NPNPM |  | -5.480 | 1.129 | 0.983 | -4.912 | 1.065 | 0.999 | -4.476 | 1.054 | 0.982 |
| NPNPP |  | -7.224 | 1.219 | 0.976 | -8.933 | 1.648 | 0.965 | -3.672 | 0.644 | 0.995 |
| NPNPV |  | -5.311 | 1.065 | 0.947 | -4.093 | 0.832 | 0.999 | -3.415 | 0.753 | 0.995 |
| NPNPY |  | -4.071 | 0.846 | 0.999 | -4.742 | 1.070 | 0.995 | -4.507 | 1.129 | 0.980 |
| CPNPT |  | -6.835 | 1.069 | 0.922 | -4.630 | 0.688 | 0.966 | -3.244 | 0.507 | 0.994 |
| IPNPT |  | -4.743 | 1.094 | 0.995 | -5.303 | 1.253 | 0.992 | -4.512 | 1.137 | 0.989 |
| LPNPT |  | -5.444 | 1.382 | 0.838 | -6.717 | 1.689 | 0.999 | -6.533 | 1.696 | 0.986 |
| MPNPT |  | -5.920 | 1.232 | 0.961 | -4.934 | 1.065 | 0.996 | -4.081 | 0.957 | 0.987 |
| QPNPT |  | -4.837 | 1.125 | 0.999 | -5.318 | 1.264 | 1.000 | -5.206 | 1.311 | 0.988 |
| VPNPT |  | -6.901 | 1.287 | 0.969 | -4.230 | 0.752 | 0.999 | -3.675 | 0.722 | 0.991 |
| YPNPT |  | -3.994 | 0.868 | 0.927 | -6.259 | 1.445 | 0.992 | -6.448 | 1.616 | 0.970 |
| APNPT | pH=4 | -6.948 | 0.752 | 0.994 | -7.697 | 0.887 | 0.997 | -7.730 | 0.937 | 0.995 |
| DPNPT |  | -6.458 | 0.722 | 0.997 | -7.152 | 0.835 | 0.997 | -7.409 | 0.958 | 0.996 |
| EPNPT |  | -7.526 | 0.845 | 0.997 | -8.104 | 0.971 | 0.998 | -8.658 | 1.091 | 0.997 |
| GPNPT |  | -6.631 | 0.631 | 0.994 | -7.320 | 0.761 | 0.997 | -7.248 | 0.811 | 0.993 |
| HPNPT |  | -6.632 | 0.487 | 0.991 | -9.094 | 0.748 | 0.996 | -9.412 | 0.818 | 0.988 |
| KPNPT |  | -6.697 | 0.237 | 0.966 | -9.009 | 0.402 | 0.981 | -9.814 | 0.485 | 0.952 |
| NPNPT |  | -6.934 | 0.647 | 0.997 | -7.340 | 0.762 | 0.995 | -7.044 | 0.810 | 0.990 |
| PPNPT |  | -8.245 | 1.044 | 0.997 | -8.860 | 1.178 | 0.998 | -9.203 | 1.269 | 0.998 |
| RPNPT |  | -4.753 | 0.195 | 0.997 | -9.187 | 0.667 | 0.963 | -9.826 | 0.791 | 0.948 |
| SPNPT |  | -6.709 | 0.646 | 0.994 | -7.505 | 0.788 | 0.997 | -7.465 | 0.845 | 0.995 |
| TPNPT |  | -7.471 | 0.912 | 1.000 | -8.480 | 1.098 | 0.998 | -8.638 | 1.173 | 0.998 |
| NPNPA |  | -6.605 | 0.656 | 0.996 | -7.205 | 0.780 | 0.998 | -7.101 | 0.821 | 0.997 |
| NPNPD |  | -6.737 | 0.487 | 0.995 | -7.238 | 0.604 | 0.995 | -7.028 | 0.668 | 0.993 |
| NPNPE |  | -7.265 | 0.651 | 0.996 | -7.887 | 0.798 | 0.997 | -7.920 | 0.890 | 0.996 |
| NPNPG |  | -6.472 | 0.449 | 0.995 | -7.275 | 0.590 | 0.997 | -7.246 | 0.657 | 0.998 |
| NPNPH |  | -5.270 | 0.045 | 0.858 | -7.232 | 0.267 | 0.962 | -7.875 | 0.383 | 0.956 |
| NPNPK |  | -7.064 | 0.120 | 0.899 | -9.978 | 0.410 | 0.926 | -9.146 | 0.357 | 0.944 |
| NPNPN |  | -6.861 | 0.345 | 1.000 | -7.665 | 0.472 | 0.999 | -7.983 | 0.582 | 0.997 |
| NPNPQ |  | -5.826 | 0.347 | 0.997 | -7.634 | 0.572 | 1.000 | -7.990 | 0.690 | 0.998 |
| NPNPR |  | -6.975 | 0.303 | 0.945 | -9.037 | 0.557 | 0.983 | -9.700 | 0.692 | 0.998 |
| NPNPS |  | -6.561 | 0.389 | 0.996 | -7.530 | 0.536 | 1.000 | -7.595 | 0.615 | 0.999 |
| HGRFA |  | -2.946 | 0.459 | 1.000 | -3.277 | 0.515 | 1.000 | -3.497 | 0.648 | 1.000 |
| HGRFD |  | -3.033 | 0.396 | 1.000 | -3.313 | 0.485 | 1.000 | -3.384 | 0.603 | 1.000 |
| HGRFE |  | -2.958 | 0.385 | 1.000 | -3.665 | 0.554 | 0.999 | -3.330 | 0.586 | 1.000 |
| HGRFG |  | -2.191 | 0.111 | 0.969 | -3.133 | 0.282 | 0.991 | -5.071 | 0.890 | 0.981 |
| HGRFH |  | -2.679 | 0.169 | 1.000 | -2.526 | 0.032 | 0.997 | -2.880 | 0.203 | 0.999 |
| HGRFK |  | -3.161 | 0.280 | 0.976 | -2.298 | 0.023 | 0.995 | -2.837 | 0.234 | 0.988 |
| HGRFN |  | -2.338 | -0.186 | 0.886 | -4.744 | 0.374 | 0.947 | -6.240 | 0.804 | 0.958 |
| HGRFQ |  | -3.208 | 0.311 | 1.000 | -6.040 | 0.695 | 0.970 | -7.007 | 1.031 | 0.999 |
| HGRFR |  | -3.892 | 0.380 | 0.959 | -10.398 | 1.417 | 0.853 | -8.262 | 1.182 | 0.870 |
| HGRFS |  | -3.130 | 0.224 | 0.999 | -6.389 | 0.795 | 0.936 | -7.080 | 1.037 | 0.977 |
| HGRFT |  | -3.087 | 0.352 | 1.000 | -3.450 | 0.448 | 1.000 | -3.392 | 0.515 | 1.000 |
| AGRFG |  | -3.380 | 0.476 | 0.997 | -4.523 | 0.818 | 0.996 | -4.357 | 0.877 | 0.996 |
| DGRFG |  | -4.307 | 0.863 | 1.000 | -4.513 | 1.007 | 1.000 | -4.585 | 1.142 | 1.000 |
| EGRFG |  | -4.495 | 0.874 | 1.000 | -4.767 | 1.029 | 1.000 | -4.816 | 1.154 | 1.000 |
| GGRFG |  | -3.221 | 0.404 | 1.000 | -4.091 | 0.678 | 0.996 | -4.052 | 0.772 | 0.995 |
| KGRFG |  | -2.793 | -0.070 | 0.929 | -5.712 | 0.609 | 0.972 | -6.960 | 0.995 | 0.982 |
| NGRFG |  | -3.703 | 0.504 | 0.998 | -4.738 | 0.824 | 0.998 | -4.683 | 0.914 | 0.998 |
| PGRFG |  | -3.333 | 0.541 | 1.000 | -4.797 | 0.953 | 0.998 | -4.592 | 0.999 | 0.997 |
| QGRFG |  | -3.155 | 0.410 | 1.000 | -4.241 | 0.737 | 0.997 | -4.141 | 0.814 | 0.996 |
| RGRFG |  | -3.123 | 0.269 | 1.000 | -3.500 | 0.390 | 1.000 | -3.388 | 0.470 | 1.000 |
| SGRFG |  | -3.175 | 0.402 | 1.000 | -4.053 | 0.674 | 0.995 | -4.014 | 0.766 | 0.996 |
| TGRFG |  | -4.060 | 0.653 | 0.996 | -4.920 | 0.939 | 0.999 | -4.874 | 1.027 | 0.999 |
| VGRFG |  | -5.328 | 1.203 | 1.000 | -5.623 | 1.371 | 1.000 | -5.662 | 1.468 | 1.000 |
| NPNPC |  | -3.355 | 0.309 | 0.990 | -3.996 | 0.496 | 0.998 | -4.110 | 0.593 | 0.997 |
| NPNPI |  | -5.053 | 1.455 | 1.000 | -5.224 | 1.552 | 1.000 | -5.246 | 1.607 | 1.000 |
| NPNPM |  | -4.873 | 1.026 | 1.000 | -5.147 | 1.151 | 1.000 | -5.200 | 1.235 | 1.000 |
| NPNPP |  | -3.772 | 0.461 | 0.999 | -5.125 | 0.770 | 0.991 | -4.848 | 0.771 | 0.987 |
| NPNPV |  | -4.707 | 0.976 | 1.000 | -4.947 | 1.072 | 1.000 | -4.926 | 1.108 | 1.000 |
| NPNPY |  | -5.128 | 1.082 | 1.000 | -5.422 | 1.256 | 1.000 | -5.461 | 1.384 | 1.000 |
| CPNPT |  | -3.582 | 0.362 | 0.998 | -4.982 | 0.668 | 0.998 | -5.146 | 0.774 | 0.994 |
| IPNPT |  | -5.846 | 1.418 | 1.000 | -6.175 | 1.540 | 1.000 | -6.415 | 1.637 | 1.000 |
| LPNPT |  | -5.933 | 1.546 | 1.000 | -6.177 | 1.655 | 1.000 | -6.281 | 1.726 | 1.000 |
| MPNPT |  | -5.506 | 1.195 | 1.000 | -5.830 | 1.325 | 1.000 | -5.993 | 1.422 | 1.000 |
| QPNPT |  | -4.790 | 0.970 | 0.999 | -5.045 | 1.060 | 1.000 | -5.165 | 1.168 | 1.000 |
| VPNPT |  | -5.144 | 0.904 | 0.999 | -5.541 | 1.019 | 0.999 | -5.359 | 1.013 | 0.998 |
| YPNPT |  | -5.834 | 1.329 | 1.000 | -6.320 | 1.535 | 1.000 | -6.663 | 1.725 | 1.000 |
| APNPT | pH=5 | -5.992 | 0.574 | 0.992 | -7.204 | 0.712 | 0.996 | -6.119 | 0.640 | 0.980 |
| DPNPT |  | -10.732 | 0.862 | 0.908 | -11.139 | 0.946 | 0.910 | -3.818 | 0.346 | 0.988 |
| EPNPT |  | -9.614 | 0.777 | 0.927 | -9.011 | 0.677 | 0.926 | -9.811 | 0.742 | 0.928 |
| GPNPT |  | -4.985 | 0.410 | 0.987 | -5.031 | 0.451 | 0.988 | -5.182 | 0.505 | 0.979 |
| HPNPT |  | -10.540 | 1.107 | 0.943 | -10.137 | 1.127 | 0.923 | -11.935 | 1.329 | 0.929 |
| KPNPT |  | -3.948 | -0.001 | 0.998 | -4.144 | 0.025 | 0.997 | -4.089 | 0.020 | 0.994 |
| NPNPT |  | -6.678 | 0.537 | 0.997 | -7.088 | 0.615 | 0.998 | -7.476 | 0.713 | 0.998 |
| PPNPT |  | -7.389 | 0.861 | 0.997 | -7.827 | 0.938 | 0.998 | -8.215 | 1.021 | 0.995 |
| RPNPT |  | -4.559 | 0.158 | 0.999 | -4.808 | 0.221 | 0.999 | -5.083 | 0.283 | 0.999 |
| SPNPT |  | -6.571 | 0.528 | 0.998 | -6.912 | 0.588 | 0.997 | -7.320 | 0.676 | 0.998 |
| TPNPT |  | -6.928 | 0.770 | 0.996 | -7.790 | 0.886 | 0.999 | -7.462 | 0.904 | 0.994 |
| NPNPA |  | -5.341 | 0.454 | 0.991 | -6.515 | 0.596 | 0.994 | -6.071 | 0.599 | 0.994 |
| NPNPD |  | -8.663 | 0.519 | 0.936 | -5.047 | 0.025 | 0.990 | -9.760 | 0.635 | 0.825 |
| NPNPE |  | -8.320 | 0.489 | 0.868 | -6.260 | 0.221 | 0.987 | -9.971 | 0.732 | 0.922 |
| NPNPG |  | -6.289 | 0.382 | 0.997 | -6.648 | 0.452 | 0.998 | -6.988 | 0.550 | 0.997 |
| NPNPH |  | -5.579 | 0.174 | 0.971 | -6.928 | 0.340 | 0.938 | -4.554 | 0.154 | 0.996 |
| NPNPK |  | -7.019 | 0.179 | 0.978 | -6.946 | 0.172 | 0.944 | -6.780 | 0.195 | 0.971 |
| NPNPN |  | -6.103 | 0.244 | 0.993 | -6.701 | 0.333 | 0.997 | -7.089 | 0.429 | 0.996 |
| NPNPQ |  | -6.821 | 0.375 | 0.992 | -7.194 | 0.447 | 0.995 | -7.774 | 0.583 | 0.997 |
| NPNPR |  | -4.403 | 0.115 | 1.000 | -4.868 | 0.210 | 0.999 | -4.891 | 0.253 | 0.999 |
| NPNPS |  | -6.104 | 0.272 | 0.995 | -6.554 | 0.345 | 0.997 | -7.026 | 0.452 | 0.996 |
| HGRFA |  | -2.878 | 0.346 | 0.944 | -0.844 | -0.027 | 0.804 | -4.589 | 0.852 | 0.762 |
| HGRFD |  | -2.842 | 0.308 | 1.000 | -2.667 | 0.332 | 0.979 | -2.800 | 0.430 | 1.000 |
| HGRFE |  | -3.463 | 0.400 | 0.989 | -2.476 | 0.246 | 0.964 | -3.681 | 0.570 | 0.991 |
| HGRFG |  | -2.087 | 0.038 | 0.998 | -2.004 | 0.047 | 0.999 | -1.979 | 0.077 | 0.999 |
| HGRFH |  | -1.125 | -0.237 | 0.995 | -1.593 | -0.070 | 0.995 | -1.427 | -0.078 | 0.993 |
| HGRFK |  | -0.920 | -0.293 | 0.965 | -1.226 | -0.170 | 0.976 | -0.897 | -0.227 | 0.979 |
| HGRFN |  | -2.062 | 0.073 | 0.994 | -2.112 | 0.191 | 0.974 | -1.573 | 0.063 | 0.992 |
| HGRFQ |  | -2.198 | 0.127 | 0.999 | -2.142 | 0.226 | 0.977 | -1.760 | 0.132 | 0.993 |
| HGRFR |  | -0.960 | -0.264 | 0.992 | -1.405 | -0.093 | 0.969 | -0.959 | -0.173 | 0.986 |
| HGRFS |  | -2.184 | 0.130 | 0.995 | -2.169 | 0.228 | 0.991 | -1.649 | 0.106 | 0.995 |
| HGRFT |  | -1.563 | 0.051 | 0.973 | -1.246 | 0.088 | 0.944 | -0.986 | 0.004 | 0.962 |
| AGRFG |  | -2.642 | 0.319 | 0.998 | -2.533 | 0.379 | 0.990 | -3.474 | 0.659 | 0.941 |
| DGRFG |  | -3.938 | 0.778 | 1.000 | -4.085 | 0.902 | 1.000 | -4.244 | 1.044 | 1.000 |
| EGRFG |  | -3.933 | 0.789 | 0.999 | -4.046 | 0.907 | 1.000 | -4.271 | 1.064 | 0.999 |
| GGRFG |  | -2.831 | 0.337 | 1.000 | -2.830 | 0.408 | 1.000 | -2.790 | 0.467 | 0.999 |
| KGRFG |  | -2.793 | 0.144 | 1.000 | -2.747 | 0.214 | 1.000 | -2.626 | 0.246 | 1.000 |
| NGRFG |  | -3.465 | 0.519 | 1.000 | -3.527 | 0.628 | 1.000 | -4.088 | 0.839 | 0.989 |
| PGRFG |  | -3.064 | 0.474 | 0.989 | -2.996 | 0.553 | 0.975 | -5.614 | 1.247 | 0.951 |
| QGRFG |  | -2.634 | 0.304 | 0.999 | -2.799 | 0.445 | 0.998 | -3.491 | 0.664 | 0.958 |
| RGRFG |  | -2.689 | 0.193 | 1.000 | -2.667 | 0.264 | 1.000 | -2.296 | 0.237 | 0.984 |
| SGRFG |  | -2.766 | 0.319 | 1.000 | -3.082 | 0.480 | 0.998 | -3.644 | 0.677 | 0.988 |
| TGRFG |  | -3.338 | 0.528 | 0.999 | -3.436 | 0.653 | 0.999 | -4.519 | 0.987 | 0.961 |
| VGRFG |  | -7.699 | 1.753 | 0.979 | -6.977 | 1.674 | 0.973 | -7.309 | 1.873 | 0.974 |
| NPNPC |  | -3.207 | 0.265 | 0.993 | -3.413 | 0.334 | 0.996 | -3.806 | 0.473 | 1.000 |
| NPNPI |  | -4.559 | 1.261 | 1.000 | -4.577 | 1.304 | 1.000 | -4.656 | 1.361 | 1.000 |
| NPNPM |  | -4.591 | 0.903 | 1.000 | -4.707 | 0.974 | 0.999 | -4.856 | 1.069 | 1.000 |
| NPNPP |  | -4.386 | 0.406 | 0.998 | -4.624 | 0.460 | 0.997 | -5.028 | 0.582 | 0.999 |
| NPNPV |  | -4.482 | 0.860 | 0.999 | -4.499 | 0.881 | 0.998 | -4.639 | 0.946 | 0.999 |
| NPNPY |  | -5.043 | 1.010 | 0.994 | -5.240 | 1.134 | 0.999 | -5.530 | 1.330 | 0.988 |
| CPNPT |  | -5.261 | 0.688 | 0.972 | -4.280 | 0.446 | 0.998 | -4.487 | 0.484 | 1.000 |
| IPNPT |  | -5.824 | 1.400 | 0.874 | -5.507 | 1.320 | 1.000 | -5.669 | 1.376 | 0.999 |
| LPNPT |  | -5.407 | 1.374 | 1.000 | -5.442 | 1.413 | 0.999 | -5.722 | 1.505 | 1.000 |
| MPNPT |  | -4.895 | 1.016 | 0.998 | -5.096 | 1.093 | 0.999 | -5.217 | 1.161 | 0.999 |
| QPNPT |  | -3.073 | 0.530 | 1.000 | -14.747 | 2.981 | 0.868 | -2.908 | 0.569 | 1.000 |
| VPNPT |  | -4.860 | 0.807 | 0.999 | -5.225 | 0.883 | 0.996 | -5.789 | 1.033 | 0.982 |
| YPNPT |  | -5.450 | 1.211 | 1.000 | -5.600 | 1.321 | 0.999 | -6.265 | 1.558 | 0.999 |
| APNPT | pH=6 | -7.673 | 0.670 | 0.998 | -8.002 | 0.731 | 0.999 | -8.120 | 0.793 | 0.999 |
| DPNPT |  | -7.989 | 0.361 | 0.996 | -8.477 | 0.463 | 0.997 | -7.794 | 0.480 | 0.986 |
| EPNPT |  | -8.361 | 0.416 | 0.998 | -9.636 | 0.542 | 0.991 | -8.735 | 0.445 | 0.996 |
| GPNPT |  | -7.519 | 0.547 | 0.999 | -7.936 | 0.631 | 0.999 | -8.063 | 0.713 | 0.999 |
| HPNPT |  | -9.023 | 1.085 | 0.999 | -9.365 | 1.211 | 0.999 | -9.334 | 1.308 | 0.998 |
| KPNPT |  | -8.365 | 0.343 | 0.991 | -9.141 | 0.383 | 0.995 | -8.425 | 0.331 | 0.965 |
| NPNPT |  | -7.620 | 0.601 | 0.998 | -8.369 | 0.720 | 1.000 | -8.344 | 0.798 | 0.999 |
| PPNPT |  | -8.375 | 0.898 | 0.999 | -8.849 | 0.987 | 0.999 | -9.380 | 1.092 | 0.998 |
| RPNPT |  | -8.277 | 0.616 | 0.995 | -9.221 | 0.720 | 0.999 | -9.239 | 0.782 | 0.997 |
| SPNPT |  | -7.680 | 0.588 | 0.999 | -8.109 | 0.667 | 1.000 | -8.200 | 0.740 | 0.999 |
| TPNPT |  | -8.026 | 0.856 | 0.999 | -8.460 | 0.934 | 0.999 | -8.646 | 1.011 | 0.999 |
| NPNPA |  | -7.042 | 0.560 | 1.000 | -7.403 | 0.633 | 1.000 | -7.516 | 0.705 | 0.999 |
| NPNPD |  | -7.984 | 0.054 | 0.926 | -6.616 | -0.048 | 0.984 | -6.188 | 0.003 | 0.944 |
| NPNPE |  | -6.083 | -0.090 | 0.982 | -6.899 | 0.029 | 0.970 | -7.220 | 0.157 | 0.962 |
| NPNPG |  | -7.878 | 0.486 | 1.000 | -8.132 | 0.557 | 1.000 | -8.035 | 0.629 | 0.999 |
| NPNPH |  | -7.734 | 0.501 | 0.999 | -8.298 | 0.581 | 0.999 | -8.256 | 0.635 | 0.998 |
| NPNPK |  | -8.676 | 0.397 | 0.999 | -9.113 | 0.436 | 0.999 | -9.019 | 0.474 | 0.998 |
| NPNPN |  | -8.255 | 0.387 | 0.999 | -8.743 | 0.479 | 1.000 | -8.675 | 0.556 | 0.999 |
| NPNPQ |  | -8.594 | 0.490 | 0.997 | -8.844 | 0.567 | 0.999 | -8.822 | 0.654 | 1.000 |
| NPNPR |  | -8.756 | 0.634 | 0.999 | -9.320 | 0.730 | 0.999 | -9.275 | 0.809 | 0.998 |
| NPNPS |  | -7.851 | 0.385 | 0.998 | -8.470 | 0.487 | 1.000 | -8.316 | 0.552 | 0.999 |
| HGRFA |  | -4.646 | 1.188 | 1.000 | -4.599 | 1.228 | 0.999 | -5.920 | 1.568 | 0.998 |
| HGRFD |  | -5.149 | 0.910 | 0.997 | -5.322 | 1.043 | 1.000 | -5.737 | 1.259 | 1.000 |
| HGRFE |  | -5.206 | 0.921 | 0.997 | -5.196 | 1.010 | 1.000 | -5.927 | 1.287 | 0.999 |
| HGRFG |  | -4.276 | 0.986 | 0.995 | -9.006 | 2.051 | 0.868 | -6.125 | 1.498 | 0.997 |
| HGRFH |  | -6.890 | 1.495 | 0.952 | -6.135 | 1.376 | 0.996 | -7.827 | 1.749 | 1.000 |
| HGRFK |  | -5.354 | 0.831 | 0.980 | -5.041 | 0.806 | 1.000 | -5.760 | 1.014 | 0.999 |
| HGRFN |  | -6.149 | 1.203 | 0.994 | -5.954 | 1.212 | 0.999 | -5.281 | 1.222 | 0.997 |
| HGRFQ |  | -8.048 | 1.619 | 0.945 | -7.612 | 1.579 | 0.992 | -5.424 | 1.257 | 1.000 |
| HGRFR |  | -7.022 | 1.270 | 0.974 | -6.572 | 1.234 | 0.999 | -5.958 | 1.192 | 1.000 |
| HGRFS |  | -5.987 | 1.224 | 0.982 | -5.966 | 1.275 | 0.989 | -4.285 | 1.043 | 0.995 |
| HGRFT |  | -5.513 | 1.303 | 0.997 | -5.356 | 1.332 | 0.999 | -5.129 | 1.367 | 1.000 |
| AGRFG |  | -4.748 | 0.916 | 0.999 | -4.831 | 1.009 | 1.000 | -4.301 | 1.039 | 0.999 |
| DGRFG |  | -4.651 | 0.877 | 1.000 | -4.644 | 0.980 | 1.000 | -4.723 | 1.139 | 0.999 |
| EGRFG |  | -4.680 | 0.898 | 1.000 | -4.732 | 1.015 | 1.000 | -4.742 | 1.156 | 0.999 |
| GGRFG |  | -4.412 | 0.761 | 0.999 | -4.623 | 0.900 | 1.000 | -4.302 | 0.997 | 0.993 |
| KGRFG |  | -5.239 | 0.740 | 1.000 | -5.391 | 0.860 | 1.000 | -5.096 | 1.055 | 0.973 |
| NGRFG |  | -4.457 | 0.928 | 1.000 | -4.517 | 1.018 | 1.000 | -4.523 | 1.139 | 1.000 |
| PGRFG |  | -4.900 | 1.031 | 0.999 | -5.016 | 1.130 | 1.000 | -4.782 | 1.207 | 1.000 |
| QGRFG |  | -4.516 | 0.819 | 0.999 | -4.514 | 0.905 | 0.999 | -4.121 | 0.977 | 0.998 |
| RGRFG |  | -6.130 | 1.021 | 0.985 | -5.907 | 1.047 | 1.000 | -4.807 | 0.970 | 1.000 |
| SGRFG |  | -4.540 | 0.897 | 0.999 | -4.507 | 0.956 | 1.000 | -4.249 | 1.028 | 1.000 |
| TGRFG |  | -4.359 | 0.985 | 1.000 | -4.488 | 1.067 | 1.000 | -4.449 | 1.168 | 1.000 |
| VGRFG |  | -4.600 | 1.389 | 0.999 | -4.828 | 1.451 | 1.000 | -4.900 | 1.548 | 1.000 |
| NPNPC |  | -4.715 | 0.476 | 0.996 | -5.012 | 0.583 | 0.996 | -4.764 | 0.628 | 0.996 |
| NPNPI |  | -5.189 | 1.344 | 1.000 | -5.194 | 1.399 | 1.000 | -5.203 | 1.466 | 1.000 |
| NPNPM |  | -5.390 | 1.014 | 1.000 | -5.475 | 1.095 | 1.000 | -5.391 | 1.165 | 1.000 |
| NPNPP |  | -6.024 | 0.607 | 1.000 | -7.989 | 1.045 | 0.973 | -6.331 | 0.736 | 0.999 |
| NPNPV |  | -5.156 | 0.919 | 1.000 | -5.237 | 0.972 | 1.000 | -5.133 | 1.009 | 1.000 |
| NPNPY |  | -5.564 | 1.036 | 1.000 | -5.548 | 1.140 | 1.000 | -5.595 | 1.294 | 1.000 |
| CPNPT |  | -6.380 | 0.912 | 0.997 | -6.757 | 0.976 | 0.991 | -5.929 | 0.804 | 0.987 |
| IPNPT |  | -5.303 | 1.308 | 1.000 | -5.685 | 1.389 | 1.000 | -6.032 | 1.484 | 1.000 |
| LPNPT |  | -5.441 | 1.419 | 1.000 | -5.752 | 1.502 | 1.000 | -5.897 | 1.568 | 1.000 |
| MPNPT |  | -5.212 | 1.129 | 1.000 | -5.571 | 1.221 | 1.000 | -5.719 | 1.293 | 0.999 |
| QPNPT |  | -4.632 | 0.506 | 1.000 | -3.413 | 0.291 | 0.978 | -7.587 | 1.294 | 0.916 |
| VPNPT |  | -5.340 | 0.901 | 1.000 | -6.164 | 1.053 | 1.000 | -6.244 | 1.062 | 1.000 |
| YPNPT |  | -5.723 | 1.264 | 1.000 | -6.071 | 1.419 | 1.000 | -6.287 | 1.576 | 1.000 |
| APNPT | pH=7 | -6.066 | 0.690 | 0.997 | -6.533 | 0.758 | 0.997 | -6.511 | 0.790 | 0.996 |
| DPNPT |  | -14.707 | 0.784 | 0.883 | -10.918 | 0.507 | 1.000 | -9.173 | 0.399 | 1.000 |
| EPNPT |  | -12.966 | 0.604 | 0.944 | -11.914 | 0.570 | 0.999 | -10.772 | 0.484 | 0.993 |
| GPNPT |  | -5.451 | 0.529 | 0.996 | -6.025 | 0.618 | 0.996 | -6.004 | 0.671 | 0.996 |
| HPNPT |  | -7.936 | 1.119 | 0.999 | -8.452 | 1.270 | 0.998 | -8.554 | 1.405 | 0.995 |
| KPNPT |  | -4.861 | 0.706 | 0.972 | -5.462 | 0.776 | 0.982 | -5.326 | 0.810 | 0.968 |
| NPNPT |  | -5.884 | 0.418 | 0.983 | -7.390 | 0.628 | 0.995 | -7.644 | 0.743 | 0.998 |
| PPNPT |  | -6.379 | 0.947 | 0.995 | -6.942 | 1.035 | 0.995 | -7.140 | 1.101 | 0.995 |
| RPNPT |  | -6.368 | 1.030 | 0.995 | -6.759 | 1.116 | 0.999 | -6.458 | 1.170 | 0.995 |
| SPNPT |  | -6.193 | 0.501 | 0.994 | -7.085 | 0.643 | 0.996 | -7.034 | 0.720 | 0.996 |
| TPNPT |  | -7.733 | 0.863 | 0.989 | -8.249 | 0.976 | 0.997 | -8.078 | 1.024 | 0.998 |
| NPNPA |  | -4.937 | 0.341 | 0.915 | -5.849 | 0.486 | 0.995 | -6.381 | 0.614 | 0.997 |
| NPNPD |  | \ | \ | \ | \ | \ | \ | \ | \ | \ |
| NPNPE |  | \ | \ | \ | \ | \ | \ | \ | \ | \ |
| NPNPG |  | -6.430 | 0.370 | 0.980 | -6.491 | 0.435 | 0.984 | -6.868 | 0.560 | 0.995 |
| NPNPH |  | -6.568 | 0.618 | 0.996 | -7.324 | 0.780 | 0.995 | -7.130 | 0.893 | 0.998 |
| NPNPK |  | -5.107 | 0.539 | 0.994 | -5.576 | 0.603 | 0.994 | -5.508 | 0.681 | 0.995 |
| NPNPN |  | -7.067 | 0.344 | 0.993 | -7.502 | 0.447 | 0.987 | -6.212 | 0.412 | 0.978 |
| NPNPQ |  | -6.109 | 0.316 | 0.980 | -7.515 | 0.504 | 0.998 | -6.761 | 0.523 | 0.983 |
| NPNPR |  | -6.007 | 0.773 | 0.995 | -6.645 | 0.885 | 0.996 | -6.824 | 1.009 | 0.993 |
| NPNPS |  | -6.589 | 0.318 | 0.995 | -7.188 | 0.436 | 0.992 | -6.169 | 0.424 | 0.980 |
| HGRFA |  | -4.240 | 1.514 | 0.999 | -4.571 | 1.668 | 0.999 | -4.695 | 1.831 | 0.983 |
| HGRFD |  | -4.593 | 0.846 | 0.995 | -5.101 | 1.069 | 0.999 | -5.984 | 1.457 | 0.980 |
| HGRFE |  | -4.832 | 0.894 | 0.998 | -5.354 | 1.116 | 1.000 | -6.058 | 1.457 | 0.982 |
| HGRFG |  | -4.479 | 1.464 | 0.998 | -4.352 | 1.522 | 0.999 | -4.386 | 1.678 | 0.992 |
| HGRFH |  | -4.420 | 1.586 | 0.999 | -4.884 | 1.794 | 0.999 | -4.722 | 1.946 | 0.988 |
| HGRFK |  | -3.603 | 1.466 | 0.968 | -3.027 | 1.320 | 0.994 | -2.752 | 1.436 | 0.882 |
| HGRFN |  | -3.792 | 1.221 | 0.998 | -4.368 | 1.418 | 0.999 | -4.290 | 1.553 | 0.965 |
| HGRFQ |  | -3.915 | 1.279 | 0.999 | -4.575 | 1.495 | 0.998 | -4.502 | 1.641 | 0.980 |
| HGRFR |  | 1.801 | 0.257 | 0.195 | -3.112 | 1.500 | 0.997 | -3.184 | 1.695 | 0.915 |
| HGRFS |  | -3.749 | 1.245 | 1.000 | -4.367 | 1.454 | 0.999 | -4.304 | 1.592 | 0.986 |
| HGRFT |  | -4.163 | 1.459 | 1.000 | -4.667 | 1.648 | 0.999 | -4.649 | 1.791 | 0.990 |
| AGRFG |  | -3.268 | 1.161 | 0.998 | -3.518 | 1.279 | 1.000 | -3.405 | 1.354 | 0.984 |
| DGRFG |  | -4.350 | 0.779 | 0.997 | -4.864 | 1.012 | 0.999 | -4.722 | 1.143 | 1.000 |
| EGRFG |  | -4.417 | 0.851 | 1.000 | -4.552 | 0.994 | 1.000 | -4.631 | 1.175 | 0.997 |
| GGRFG |  | -2.882 | 0.977 | 0.994 | -3.205 | 1.122 | 1.000 | -3.143 | 1.244 | 0.991 |
| KGRFG |  | -1.201 | 1.006 | 0.978 | -2.435 | 1.329 | 0.998 | -1.591 | 1.284 | 0.949 |
| NGRFG |  | -3.640 | 1.018 | 0.999 | -3.989 | 1.167 | 1.000 | -4.096 | 1.314 | 0.997 |
| PGRFG |  | -2.964 | 1.381 | 0.999 | -3.346 | 1.493 | 0.999 | -3.320 | 1.552 | 0.975 |
| QGRFG |  | -2.789 | 0.997 | 0.994 | -3.177 | 1.155 | 1.000 | -4.950 | 1.613 | 0.993 |
| RGRFG |  | -2.877 | 1.014 | 0.997 | -3.213 | 1.157 | 1.000 | -3.178 | 1.265 | 0.989 |
| SGRFG |  | -3.444 | 1.010 | 0.999 | -0.877 | 0.537 | 0.118 | -3.842 | 1.296 | 0.995 |
| TGRFG |  | -3.817 | 1.201 | 0.998 | -4.140 | 1.351 | 0.999 | -4.203 | 1.476 | 0.998 |
| VGRFG |  | -4.615 | 1.896 | 1.000 | -4.748 | 1.982 | 1.000 | -4.712 | 2.036 | 0.997 |
| NPNPC |  | \ | \ | \ | \ | \ | \ | \ | \ | \ |
| NPNPI |  | -5.641 | 1.202 | 1.000 | -5.818 | 1.309 | 1.000 | -5.749 | 1.390 | 0.995 |
| NPNPM |  | -4.264 | 0.615 | 0.991 | -5.596 | 0.958 | 0.997 | -5.698 | 1.099 | 0.999 |
| NPNPP |  | -3.916 | 0.194 | 0.985 | -6.495 | 0.780 | 0.959 | -5.270 | 0.630 | 0.960 |
| NPNPV |  | -5.520 | 0.839 | 0.948 | -4.567 | 0.660 | 0.984 | -4.761 | 0.791 | 0.997 |
| NPNPY |  | -4.232 | 0.607 | 0.994 | -5.767 | 1.020 | 0.997 | -5.896 | 1.211 | 0.998 |
| CPNPT |  | -5.966 | 0.732 | 0.964 | -6.810 | 0.934 | 0.953 | -5.594 | 0.803 | 0.711 |
| IPNPT |  | -5.551 | 1.476 | 0.999 | -5.563 | 1.510 | 0.999 | -5.472 | 1.515 | 0.999 |
| LPNPT |  | -5.666 | 1.560 | 0.999 | -5.618 | 1.586 | 1.000 | -5.545 | 1.605 | 1.000 |
| MPNPT |  | -5.502 | 1.193 | 1.000 | -5.747 | 1.308 | 1.000 | -5.792 | 1.399 | 0.999 |
| QPNPT |  | -3.919 | 0.239 | 0.965 | -5.881 | 0.682 | 0.972 | -5.528 | 0.696 | 0.985 |
| VPNPT |  | -4.846 | 0.980 | 0.999 | -5.068 | 1.043 | 1.000 | -4.753 | 0.998 | 1.000 |
| YPNPT |  | -5.792 | 1.250 | 0.998 | -6.704 | 1.568 | 0.996 | -6.431 | 1.644 | 0.995 |
| APNPT | pH=8 | -7.248 | 0.901 | 0.997 | -8.038 | 1.025 | 0.998 | -8.445 | 1.056 | 0.999 |
| DPNPT |  | -9.981 | 0.438 | 0.895 | -9.686 | 0.500 | 0.997 | -10.374 | 0.569 | 0.983 |
| EPNPT |  | -12.488 | 0.636 | 0.978 | -9.624 | 0.458 | 0.986 | -9.598 | 0.382 | 0.974 |
| GPNPT |  | -6.719 | 0.660 | 0.997 | -7.778 | 0.795 | 0.999 | -8.219 | 0.850 | 0.999 |
| HPNPT |  | -8.546 | 1.265 | 0.998 | -9.350 | 1.496 | 0.998 | -9.839 | 1.632 | 0.998 |
| KPNPT |  | -6.485 | 0.739 | 0.993 | -8.179 | 0.849 | 0.999 | -8.735 | 0.925 | 1.000 |
| NPNPT |  | -7.473 | 0.677 | 0.999 | -8.593 | 0.871 | 0.999 | -8.965 | 0.959 | 0.998 |
| PPNPT |  | -7.393 | 1.302 | 0.992 | -7.711 | 1.286 | 0.997 | -7.824 | 1.275 | 0.996 |
| RPNPT |  | -7.553 | 1.032 | 0.998 | -8.884 | 1.175 | 0.998 | -9.399 | 1.315 | 0.998 |
| SPNPT |  | -7.331 | 0.689 | 1.000 | -8.230 | 0.847 | 0.999 | -8.583 | 0.916 | 0.999 |
| TPNPT |  | -7.691 | 0.963 | 0.998 | -8.714 | 1.147 | 0.998 | -9.096 | 1.208 | 0.998 |
| NPNPA |  | -6.775 | 0.612 | 0.999 | -8.077 | 0.813 | 0.999 | -8.173 | 0.860 | 0.998 |
| NPNPD |  | \ | \ | \ | \ | \ | \ | \ | \ | \ |
| NPNPE |  | \ | \ | \ | -24.291 | 1.629 | 0.786 | \ | \ | \ |
| NPNPG |  | -7.249 | 0.536 | 0.995 | -8.505 | 0.735 | 1.000 | -8.834 | 0.822 | 1.000 |
| NPNPH |  | -7.482 | 0.720 | 0.997 | -8.594 | 0.935 | 0.999 | -8.852 | 1.037 | 0.998 |
| NPNPK |  | -6.416 | 0.504 | 0.997 | -8.342 | 0.661 | 0.998 | -8.484 | 0.742 | 0.998 |
| NPNPN |  | -7.250 | 0.441 | 0.991 | -8.922 | 0.674 | 1.000 | -9.541 | 0.788 | 1.000 |
| NPNPQ |  | -7.795 | 0.554 | 0.998 | -9.234 | 0.772 | 1.000 | -9.451 | 0.852 | 1.000 |
| NPNPR |  | -7.031 | 0.732 | 0.995 | -8.802 | 0.928 | 0.999 | -9.131 | 1.064 | 0.998 |
| NPNPS |  | -7.166 | 0.460 | 0.995 | -8.741 | 0.683 | 1.000 | -9.165 | 0.776 | 1.000 |
| HGRFA |  | -4.973 | 1.599 | 0.998 | -5.330 | 1.730 | 0.999 | -5.412 | 1.869 | 0.999 |
| HGRFD |  | -5.226 | 1.068 | 0.996 | -5.022 | 1.112 | 0.998 | -5.592 | 1.339 | 0.999 |
| HGRFE |  | -4.777 | 0.983 | 1.000 | -5.351 | 1.192 | 0.998 | -5.701 | 1.357 | 0.999 |
| HGRFG |  | -4.787 | 1.455 | 0.999 | -5.228 | 1.599 | 1.000 | -5.392 | 1.762 | 0.999 |
| HGRFH |  | -5.338 | 1.673 | 0.999 | -5.907 | 1.874 | 0.999 | -6.120 | 2.078 | 0.998 |
| HGRFK |  | -4.002 | 1.391 | 0.998 | -4.193 | 1.169 | 0.998 | -4.485 | 1.339 | 0.999 |
| HGRFN |  | -4.588 | 1.307 | 0.999 | -5.203 | 1.464 | 0.999 | -5.312 | 1.619 | 0.999 |
| HGRFQ |  | -4.843 | 1.372 | 0.998 | -5.461 | 1.543 | 0.999 | -5.609 | 1.708 | 0.999 |
| HGRFR |  | -3.253 | 1.176 | 0.992 | -4.907 | 1.442 | 0.999 | -5.103 | 1.625 | 0.998 |
| HGRFS |  | -4.526 | 1.323 | 0.999 | -5.137 | 1.487 | 1.000 | -5.251 | 1.638 | 0.999 |
| HGRFT |  | -4.928 | 1.543 | 0.999 | -5.353 | 1.680 | 1.000 | -5.460 | 1.830 | 0.999 |
| AGRFG |  | -4.433 | 1.289 | 0.999 | -4.595 | 1.357 | 1.000 | -4.505 | 1.424 | 0.999 |
| DGRFG |  | -5.558 | 1.066 | 0.999 | -5.284 | 1.148 | 0.999 | -5.420 | 1.277 | 0.999 |
| EGRFG |  | -5.387 | 1.025 | 0.999 | -5.288 | 1.134 | 1.000 | -5.474 | 1.274 | 1.000 |
| GGRFG |  | -4.435 | 1.138 | 0.998 | -4.288 | 1.137 | 1.000 | -4.385 | 1.257 | 1.000 |
| KGRFG |  | -2.334 | 1.092 | 1.000 | -3.246 | 0.910 | 1.000 | -3.489 | 1.060 | 0.997 |
| NGRFG |  | -4.114 | 1.042 | 0.997 | -4.647 | 1.169 | 1.000 | -4.791 | 1.328 | 1.000 |
| PGRFG |  | -4.758 | 1.786 | 0.998 | -4.762 | 1.789 | 1.000 | -5.668 | 2.011 | 0.714 |
| QGRFG |  | -4.304 | 1.195 | 0.997 | -3.986 | 1.124 | 0.998 | -4.239 | 1.274 | 1.000 |
| RGRFG |  | -3.640 | 1.031 | 1.000 | -3.799 | 1.070 | 0.999 | -4.204 | 1.267 | 1.000 |
| SGRFG |  | -4.091 | 1.030 | 0.998 | -4.449 | 1.154 | 1.000 | -4.552 | 1.284 | 1.000 |
| TGRFG |  | -4.426 | 1.241 | 0.999 | -4.726 | 1.359 | 1.000 | -4.691 | 1.452 | 0.999 |
| VGRFG |  | -5.544 | 2.085 | 0.999 | -5.694 | 2.184 | 1.000 | -5.691 | 2.272 | 0.999 |
| NPNPC |  | -3.738 | 0.301 | 0.996 | -6.179 | 0.860 | 0.998 | -6.395 | 0.938 | 0.999 |
| NPNPI |  | -5.341 | 1.257 | 1.000 | -5.981 | 1.522 | 0.999 | -6.173 | 1.579 | 0.999 |
| NPNPM |  | -6.444 | 1.198 | 0.998 | -6.383 | 1.290 | 1.000 | -7.001 | 1.466 | 1.000 |
| NPNPP |  | -4.674 | 0.417 | 0.977 | -8.176 | 1.133 | 0.996 | -8.027 | 1.091 | 0.980 |
| NPNPV |  | -6.046 | 1.044 | 0.998 | -6.248 | 1.160 | 1.000 | -6.871 | 1.296 | 1.000 |
| NPNPY |  | -6.631 | 1.206 | 0.997 | -6.542 | 1.331 | 1.000 | -6.923 | 1.507 | 0.999 |
| CPNPT |  | -9.277 | 1.596 | 0.981 | -7.753 | 1.480 | 0.986 | -7.612 | 1.381 | 0.981 |
| IPNPT |  | -6.618 | 1.936 | 0.997 | -6.474 | 1.999 | 0.999 | -6.433 | 1.969 | 0.998 |
| LPNPT |  | -7.396 | 2.112 | 0.830 | -6.404 | 2.025 | 0.999 | -6.286 | 1.973 | 0.999 |
| MPNPT |  | -6.470 | 1.576 | 0.998 | -6.366 | 1.659 | 0.999 | -6.562 | 1.718 | 0.999 |
| QPNPT |  | -4.729 | 0.508 | 0.973 | -7.074 | 0.992 | 0.990 | -7.402 | 1.042 | 0.983 |
| VPNPT |  | -6.285 | 1.474 | 0.999 | -6.274 | 1.544 | 0.999 | -6.506 | 1.567 | 0.999 |
| YPNPT |  | -6.815 | 1.592 | 0.999 | -6.747 | 1.716 | 0.999 | -6.992 | 1.842 | 0.999 |

| Pentapeptide sequence | pH | $logk=q+pP_{m}^{N}$ | | | | | | | | |
| --- | --- | --- | --- | --- | --- | --- | --- | --- | --- | --- |
|  |  | 45℃ | | | 35℃ | | | 25℃ | | |
|  |  | p | q | R^2^ | p | q | R^2^ | p | q | R^2^ |
| APNPT | pH=2 | 6.876 | -5.367 | 0.996 | 6.909 | -5.320 | 0.998 | 7.212 | -5.488 | 0.997 |
| DPNPT |  | 6.716 | -5.270 | 0.996 | 6.784 | -5.242 | 0.998 | 7.149 | -5.442 | 0.997 |
| EPNPT |  | 7.250 | -5.637 | 0.997 | 7.335 | -5.615 | 0.998 | 7.756 | -5.855 | 0.997 |
| GPNPT |  | 6.293 | -4.982 | 0.995 | 6.355 | -4.956 | 0.998 | 6.663 | -5.121 | 0.997 |
| HPNPT |  | 7.615 | -6.047 | 0.997 | 7.830 | -6.098 | 0.998 | 8.307 | -6.380 | 0.996 |
| KPNPT |  | 6.464 | -5.236 | 0.994 | 6.436 | -5.115 | 0.998 | 6.722 | -5.284 | 0.996 |
| NPNPT |  | 6.616 | -5.258 | 0.996 | 6.703 | -5.248 | 0.998 | 7.107 | -5.481 | 0.997 |
| PPNPT |  | 7.772 | -5.969 | 0.997 | 7.882 | -5.977 | 0.998 | 8.289 | -6.217 | 0.997 |
| RPNPT |  | 7.648 | -6.033 | 0.996 | 7.860 | -6.077 | 0.997 | 8.298 | -6.327 | 0.997 |
| SPNPT |  | 6.464 | -5.109 | 0.997 | 6.597 | -5.141 | 0.998 | 6.915 | -5.314 | 0.997 |
| TPNPT |  | 7.366 | -5.678 | 0.998 | 7.475 | -5.685 | 0.998 | 7.783 | -5.846 | 0.997 |
| NPNPA |  | 6.423 | -5.087 | 0.996 | 6.452 | -5.038 | 0.996 | 6.875 | -5.308 | 0.994 |
| NPNPD |  | 6.056 | -4.973 | 0.995 | 6.032 | -4.873 | 0.997 | 6.437 | -5.109 | 0.995 |
| NPNPE |  | 6.684 | -5.340 | 0.995 | 6.665 | -5.228 | 0.997 | 7.191 | -5.550 | 0.995 |
| NPNPG |  | 5.948 | -4.910 | 0.995 | 5.918 | -4.816 | 0.997 | 6.313 | -5.060 | 0.995 |
| NPNPH |  | 6.429 | -5.354 | 0.993 | 6.382 | -5.209 | 0.997 | 6.827 | -5.485 | 0.995 |
| NPNPK |  | 6.379 | -5.349 | 0.994 | 6.316 | -5.205 | 0.997 | 6.706 | -5.457 | 0.994 |
| NPNPN |  | 5.849 | -4.996 | 0.996 | 5.851 | -4.935 | 0.998 | 6.280 | -5.212 | 0.995 |
| NPNPQ |  | 6.222 | -5.195 | 0.996 | 6.269 | -5.163 | 0.998 | 6.741 | -5.465 | 0.995 |
| NPNPR |  | 7.061 | -5.768 | 0.995 | 7.128 | -5.703 | 0.997 | 7.637 | -6.017 | 0.995 |
| NPNPS |  | 6.005 | -4.997 | 0.996 | 6.059 | -4.969 | 0.998 | 6.429 | -5.180 | 0.997 |
| HGRFA |  | 5.402 | -3.470 | 1.000 | 5.716 | -3.500 | 1.000 | 6.466 | -3.934 | 0.997 |
| HGRFD |  | 5.314 | -3.495 | 1.000 | 5.664 | -3.530 | 1.000 | 6.511 | -3.997 | 0.998 |
| HGRFE |  | 5.419 | -3.564 | 1.000 | 5.807 | -3.641 | 1.000 | 6.509 | -4.017 | 0.998 |
| HGRFG |  | 4.512 | -2.873 | 0.781 | 5.483 | -3.409 | 1.000 | 6.262 | -3.857 | 0.999 |
| HGRFH |  | 5.431 | -3.765 | 1.000 | 5.800 | -3.814 | 1.000 | 6.789 | -4.421 | 0.996 |
| HGRFK |  | 5.119 | -3.584 | 1.000 | 5.640 | -3.751 | 1.000 | 6.609 | -4.361 | 0.996 |
| HGRFN |  | 5.062 | -3.493 | 1.000 | 5.287 | -3.466 | 1.000 | 6.117 | -3.949 | 0.998 |
| HGRFQ |  | 5.234 | -3.548 | 1.000 | 5.544 | -3.580 | 1.000 | 6.289 | -3.999 | 0.998 |
| HGRFR |  | 5.569 | -3.821 | 1.000 | 6.185 | -4.046 | 1.000 | 7.134 | -4.615 | 0.996 |
| HGRFS |  | 5.189 | -3.483 | 1.000 | 5.446 | -3.470 | 1.000 | 6.294 | -3.962 | 0.998 |
| HGRFT |  | 5.554 | -3.599 | 1.000 | 5.831 | -3.592 | 1.000 | 6.684 | -4.082 | 0.997 |
| AGRFG |  | 4.781 | -3.061 | 1.000 | 4.864 | -2.962 | 1.000 | 5.412 | -3.245 | 0.999 |
| DGRFG |  | 4.749 | -3.064 | 1.000 | 4.855 | -2.982 | 1.000 | 5.402 | -3.255 | 0.999 |
| EGRFG |  | 4.927 | -3.154 | 1.000 | 5.046 | -3.075 | 1.000 | 5.625 | -3.371 | 0.999 |
| GGRFG |  | 4.579 | -3.004 | 1.000 | 4.772 | -2.982 | 1.000 | 5.250 | -3.218 | 0.999 |
| KGRFG |  | 5.106 | -3.346 | 1.000 | 5.300 | -3.298 | 1.000 | 5.993 | -3.687 | 0.998 |
| NGRFG |  | 4.771 | -3.106 | 1.000 | 4.877 | -3.024 | 1.000 | 5.401 | -3.284 | 0.999 |
| PGRFG |  | 5.183 | -3.268 | 1.000 | 5.325 | -3.208 | 1.000 | 5.881 | -3.492 | 0.999 |
| QGRFG |  | 4.671 | -2.972 | 1.000 | 4.772 | -2.882 | 1.000 | 5.342 | -3.177 | 0.999 |
| RGRFG |  | 5.344 | -3.467 | 1.000 | 5.696 | -3.524 | 1.000 | 6.477 | -3.963 | 0.998 |
| SGRFG |  | 4.728 | -3.065 | 1.000 | 4.828 | -2.977 | 1.000 | 5.364 | -3.252 | 0.999 |
| TGRFG |  | 4.183 | -2.667 | 0.715 | 5.009 | -3.063 | 1.000 | 5.564 | -3.354 | 0.999 |
| VGRFG |  | 5.651 | -3.419 | 1.000 | 5.733 | -3.306 | 0.999 | 6.027 | -3.407 | 0.997 |
| NPNPC |  | 8.068 | -5.563 | 1.000 | 8.326 | -5.578 | 0.997 | 9.343 | -6.152 | 0.999 |
| NPNPI |  | 5.186 | -3.010 | 0.996 | 5.113 | -2.872 | 0.996 | 5.549 | -3.103 | 1.000 |
| NPNPM |  | 4.953 | -3.206 | 1.000 | 4.851 | -3.030 | 0.997 | 5.310 | -3.264 | 1.000 |
| NPNPP |  | 5.082 | -3.615 | 1.000 | 5.009 | -3.484 | 0.995 | 5.562 | -3.807 | 0.999 |
| NPNPV |  | 4.816 | -3.132 | 1.000 | 4.644 | -2.927 | 0.996 | 5.029 | -3.135 | 1.000 |
| NPNPY |  | 5.226 | -3.399 | 1.000 | 5.179 | -3.234 | 0.999 | 5.610 | -3.405 | 1.000 |
| CPNPT |  | 4.692 | -3.412 | 1.000 | 4.624 | -3.288 | 0.996 | 5.081 | -3.546 | 0.999 |
| IPNPT |  | 6.348 | -4.050 | 1.000 | 6.245 | -3.876 | 0.996 | 6.773 | -4.178 | 1.000 |
| LPNPT |  | 6.462 | -4.025 | 1.000 | 6.334 | -3.827 | 0.996 | 6.828 | -4.098 | 1.000 |
| MPNPT |  | 5.882 | -3.850 | 1.000 | 5.839 | -3.714 | 0.996 | 6.350 | -3.987 | 1.000 |
| QPNPT |  | 5.152 | -3.714 | 1.000 | 5.083 | -3.613 | 0.996 | 5.506 | -3.838 | 0.999 |
| VPNPT |  | 5.602 | -3.836 | 1.000 | 5.535 | -3.708 | 0.995 | 6.071 | -4.026 | 1.000 |
| YPNPT |  | 6.448 | -4.258 | 0.999 | 6.508 | -4.167 | 0.997 | 7.136 | -4.472 | 1.000 |
| APNPT | pH=3 | 4.825 | -4.136 | 0.980 | 5.654 | -4.816 | 0.983 | 5.237 | -4.390 | 0.960 |
| DPNPT |  | 5.719 | -4.821 | 0.996 | 6.324 | -5.287 | 0.997 | 6.511 | -5.355 | 0.992 |
| EPNPT |  | 6.413 | -5.425 | 0.995 | 7.066 | -5.920 | 0.997 | 7.494 | -6.202 | 0.991 |
| GPNPT |  | 3.740 | -3.268 | 0.996 | 4.547 | -3.937 | 0.984 | 4.162 | -3.522 | 0.978 |
| HPNPT |  | 10.894 | -9.928 | 0.955 | 10.672 | -9.641 | 0.969 | 3.872 | -3.477 | 0.995 |
| KPNPT |  | 14.625 | -13.267 | 0.918 | 17.828 | -16.105 | 0.932 | 2.903 | -2.722 | 0.993 |
| NPNPT |  | 5.996 | -5.276 | 0.994 | 6.744 | -5.865 | 0.997 | 6.270 | -5.368 | 0.972 |
| PPNPT |  | 6.893 | -5.760 | 0.995 | 7.607 | -6.313 | 0.997 | 7.935 | -6.528 | 0.990 |
| RPNPT |  | 12.062 | -10.984 | 0.988 | 12.498 | -11.247 | 0.957 | 3.608 | -3.239 | 0.996 |
| SPNPT |  | 6.077 | -5.296 | 0.996 | 6.110 | -5.262 | 0.993 | 6.043 | -5.123 | 0.976 |
| TPNPT |  | 5.930 | -4.997 | 0.983 | 6.797 | -5.691 | 0.991 | 6.695 | -5.539 | 0.969 |
| NPNPA |  | 4.155 | -3.614 | 0.985 | 5.070 | -4.371 | 0.975 | 4.762 | -4.032 | 0.963 |
| NPNPD |  | 6.299 | -5.654 | 0.994 | 6.500 | -5.765 | 0.992 | 4.963 | -4.309 | 0.975 |
| NPNPE |  | 6.302 | -5.513 | 0.996 | 6.835 | -5.899 | 0.997 | 6.456 | -5.456 | 0.980 |
| NPNPG |  | 6.617 | -5.979 | 0.980 | 7.018 | -6.286 | 0.994 | 4.380 | -3.854 | 0.980 |
| NPNPH |  | 11.825 | -11.092 | 0.997 | 14.099 | -13.030 | 0.955 | 6.603 | -6.158 | 0.888 |
| NPNPK |  | \ | \ | \ | \ | \ | \ | 15.663 | -14.366 | 0.883 |
| NPNPN |  | 5.616 | -5.249 | 0.945 | 6.893 | -6.338 | 0.987 | 6.976 | -6.287 | 0.987 |
| NPNPQ |  | 6.971 | -6.312 | 0.970 | 6.986 | -6.268 | 0.951 | 4.410 | -3.897 | 0.986 |
| NPNPR |  | 10.670 | -9.967 | 0.963 | 13.098 | -11.950 | 0.967 | 3.740 | -3.432 | 0.994 |
| NPNPS |  | 6.632 | -6.025 | 0.976 | 7.278 | -6.554 | 0.993 | 4.370 | -3.873 | 0.974 |
| HGRFA |  | 3.279 | -3.580 | 0.961 | 3.276 | -2.805 | 0.999 | 2.443 | -1.995 | 0.992 |
| HGRFD |  | 2.772 | -2.377 | 0.999 | 2.704 | -2.287 | 1.000 | 2.311 | -1.813 | 0.992 |
| HGRFE |  | 2.896 | -2.529 | 0.995 | 2.881 | -2.478 | 1.000 | 2.343 | -1.898 | 0.991 |
| HGRFG |  | 7.539 | -6.218 | 0.739 | 2.627 | -2.600 | 0.998 | 2.542 | -2.106 | 0.987 |
| HGRFH |  | 1.244 | -1.441 | 0.987 | 1.865 | -1.912 | 0.953 | 2.061 | -1.828 | 0.991 |
| HGRFK |  | 1.826 | -1.873 | 0.994 | 3.035 | -2.735 | 0.970 | 1.840 | -1.628 | 0.993 |
| HGRFN |  | \ | \ | \ | \ | \ | \ | 2.245 | -2.012 | 0.968 |
| HGRFQ |  | \ | \ | \ | \ | \ | \ | 2.712 | -2.303 | 0.988 |
| HGRFR |  | 2.037 | -2.013 | 0.997 | 3.127 | -2.782 | 0.985 | 2.164 | -1.837 | 0.992 |
| HGRFS |  | \ | \ | \ | \ | \ | \ | 2.171 | -1.956 | 0.971 |
| HGRFT |  | \ | \ | \ | \ | \ | \ | 2.741 | -2.299 | 0.989 |
| AGRFG |  | 3.748 | -3.283 | 0.957 | 3.195 | -2.617 | 0.999 | 2.535 | -1.978 | 0.991 |
| DGRFG |  | 4.964 | -3.904 | 0.996 | 4.912 | -3.770 | 0.979 | 3.334 | -2.466 | 0.988 |
| EGRFG |  | 4.757 | -3.941 | 0.999 | 6.357 | -5.013 | 0.994 | 3.086 | -2.383 | 0.989 |
| GGRFG |  | 2.917 | -2.701 | 0.987 | 7.136 | -5.563 | 0.830 | 2.542 | -1.995 | 0.992 |
| KGRFG |  | \ | \ | \ | \ | \ | \ | 5.832 | -4.967 | 0.902 |
| NGRFG |  | 4.167 | -3.680 | 0.958 | 5.182 | -4.371 | 0.974 | 4.642 | -3.783 | 0.980 |
| PGRFG |  | 6.717 | -5.311 | 0.928 | 3.211 | -2.563 | 0.999 | 2.662 | -2.015 | 0.992 |
| QGRFG |  | 3.649 | -3.244 | 0.939 | 7.504 | -5.967 | 0.932 | 2.706 | -2.133 | 0.991 |
| RGRFG |  | 6.542 | -5.284 | 0.835 | 3.076 | -2.545 | 0.999 | 2.622 | -2.176 | 0.992 |
| SGRFG |  | 3.061 | -2.832 | 0.991 | 5.116 | -4.291 | 0.981 | 2.769 | -2.201 | 0.991 |
| TGRFG |  | 4.109 | -3.553 | 0.966 | 6.077 | -4.929 | 0.998 | 2.915 | -2.295 | 0.989 |
| VGRFG |  | 6.715 | -5.123 | 0.946 | 3.132 | -2.394 | 0.999 | 2.781 | -2.005 | 0.990 |
| NPNPC |  | 3.054 | -2.660 | 0.989 | 3.904 | -3.263 | 0.996 | 4.978 | -3.906 | 0.969 |
| NPNPI |  | 5.097 | -3.482 | 1.000 | 5.327 | -3.590 | 1.000 | 5.431 | -3.614 | 0.994 |
| NPNPM |  | 5.053 | -3.791 | 0.983 | 4.535 | -3.350 | 0.999 | 4.136 | -2.971 | 0.982 |
| NPNPP |  | 6.673 | -5.274 | 0.976 | 8.234 | -6.370 | 0.965 | 3.391 | -2.656 | 0.995 |
| NPNPV |  | 4.895 | -3.701 | 0.947 | 3.779 | -2.846 | 0.999 | 3.154 | -2.316 | 0.995 |
| NPNPY |  | 3.759 | -2.813 | 0.999 | 4.379 | -3.192 | 0.995 | 4.164 | -2.923 | 0.980 |
| CPNPT |  | 6.322 | -5.082 | 0.922 | 4.268 | -3.468 | 0.966 | 2.995 | -2.408 | 0.994 |
| IPNPT |  | 4.380 | -3.168 | 0.995 | 4.898 | -3.514 | 0.992 | 4.168 | -2.919 | 0.989 |
| LPNPT |  | 5.016 | -3.503 | 0.838 | 6.201 | -4.347 | 0.999 | 6.035 | -4.177 | 0.986 |
| MPNPT |  | 5.457 | -4.082 | 0.961 | 4.557 | -3.369 | 0.996 | 3.770 | -2.711 | 0.987 |
| QPNPT |  | 4.466 | -3.222 | 0.999 | 4.910 | -3.515 | 1.000 | 4.809 | -3.368 | 0.988 |
| VPNPT |  | 6.369 | -4.912 | 0.969 | 3.905 | -3.049 | 0.999 | 3.394 | -2.581 | 0.991 |
| YPNPT |  | 3.694 | -2.726 | 0.927 | 5.781 | -4.181 | 0.992 | 5.959 | -4.182 | 0.970 |
| APNPT | pH=4 | 5.780 | -4.990 | 0.994 | 6.403 | -5.473 | 0.997 | 6.431 | -5.451 | 0.995 |
| DPNPT |  | 5.372 | -4.615 | 0.997 | 5.949 | -5.075 | 0.997 | 6.163 | -5.164 | 0.996 |
| EPNPT |  | 6.260 | -5.374 | 0.997 | 6.741 | -5.726 | 0.998 | 7.202 | -6.063 | 0.997 |
| GPNPT |  | 5.516 | -4.849 | 0.994 | 6.089 | -5.288 | 0.997 | 6.030 | -5.179 | 0.993 |
| HPNPT |  | 5.518 | -4.994 | 0.991 | 7.562 | -6.764 | 0.996 | 7.824 | -6.955 | 0.988 |
| KPNPT |  | 5.569 | -5.295 | 0.966 | 7.491 | -7.041 | 0.981 | 8.164 | -7.626 | 0.952 |
| NPNPT |  | 5.767 | -5.082 | 0.997 | 6.106 | -5.304 | 0.995 | 5.861 | -5.012 | 0.990 |
| PPNPT |  | 6.858 | -5.769 | 0.997 | 7.369 | -6.142 | 0.998 | 7.655 | -6.335 | 0.998 |
| RPNPT |  | 3.954 | -3.733 | 0.997 | 7.629 | -6.914 | 0.963 | 8.158 | -7.315 | 0.948 |
| SPNPT |  | 5.581 | -4.898 | 0.994 | 6.243 | -5.414 | 0.997 | 6.211 | -5.324 | 0.995 |
| TPNPT |  | 6.212 | -5.260 | 1.000 | 7.053 | -5.909 | 0.998 | 7.185 | -5.965 | 0.998 |
| NPNPA |  | 5.494 | -4.802 | 0.996 | 5.992 | -5.173 | 0.998 | 5.907 | -5.047 | 0.997 |
| NPNPD |  | 5.605 | -5.080 | 0.995 | 6.021 | -5.377 | 0.995 | 5.847 | -5.140 | 0.993 |
| NPNPE |  | 6.044 | -5.352 | 0.996 | 6.560 | -5.719 | 0.997 | 6.588 | -5.655 | 0.996 |
| NPNPG |  | 5.384 | -4.900 | 0.995 | 6.052 | -5.421 | 0.997 | 6.026 | -5.329 | 0.998 |
| NPNPH |  | 4.394 | -4.319 | 0.858 | 6.022 | -5.715 | 0.962 | 6.558 | -6.131 | 0.956 |
| NPNPK |  | 5.859 | -5.702 | 0.899 | 8.282 | -7.819 | 0.926 | 7.596 | -7.190 | 0.944 |
| NPNPN |  | 5.705 | -5.323 | 1.000 | 6.374 | -5.860 | 0.999 | 6.639 | -6.014 | 0.997 |
| NPNPQ |  | 4.845 | -4.466 | 0.997 | 6.348 | -5.734 | 1.000 | 6.643 | -5.910 | 0.998 |
| NPNPR |  | 5.805 | -5.463 | 0.945 | 7.519 | -6.913 | 0.983 | 8.067 | -7.322 | 0.998 |
| NPNPS |  | 5.453 | -5.029 | 0.996 | 6.261 | -5.684 | 1.000 | 6.315 | -5.659 | 0.999 |
| HGRFA |  | 2.719 | -2.187 | 1.000 | 3.025 | -2.430 | 1.000 | 3.228 | -2.494 | 1.000 |
| HGRFD |  | 2.799 | -2.328 | 1.000 | 3.058 | -2.491 | 1.000 | 3.123 | -2.437 | 1.000 |
| HGRFE |  | 2.731 | -2.273 | 1.000 | 3.383 | -2.739 | 0.999 | 3.074 | -2.405 | 1.000 |
| HGRFG |  | 2.020 | -1.856 | 0.969 | 2.890 | -2.532 | 0.991 | 4.676 | -3.662 | 0.981 |
| HGRFH |  | 2.472 | -2.237 | 1.000 | 2.331 | -2.237 | 0.997 | 2.658 | -2.384 | 0.999 |
| HGRFK |  | 2.914 | -2.558 | 0.976 | 2.120 | -2.041 | 0.995 | 2.617 | -2.313 | 0.988 |
| HGRFN |  | 2.164 | -2.290 | 0.886 | 4.386 | -3.893 | 0.947 | 5.767 | -4.807 | 0.958 |
| HGRFQ |  | 2.961 | -2.572 | 1.000 | 5.582 | -4.737 | 0.970 | 6.467 | -5.264 | 0.999 |
| HGRFR |  | 3.588 | -3.113 | 0.959 | 9.568 | -7.904 | 0.853 | 7.643 | -6.253 | 0.870 |
| HGRFS |  | 2.889 | -2.588 | 0.999 | 5.909 | -4.953 | 0.936 | 6.542 | -5.329 | 0.977 |
| HGRFT |  | 2.850 | -2.422 | 1.000 | 3.184 | -2.651 | 1.000 | 3.130 | -2.532 | 1.000 |
| AGRFG |  | 3.121 | -2.562 | 0.997 | 4.177 | -3.247 | 0.996 | 4.024 | -3.040 | 0.996 |
| DGRFG |  | 3.975 | -3.006 | 1.000 | 4.165 | -3.047 | 1.000 | 4.232 | -2.977 | 1.000 |
| EGRFG |  | 4.149 | -3.164 | 1.000 | 4.400 | -3.255 | 1.000 | 4.445 | -3.173 | 1.000 |
| GGRFG |  | 2.974 | -2.490 | 1.000 | 3.778 | -2.999 | 0.996 | 3.742 | -2.870 | 0.995 |
| KGRFG |  | 2.584 | -2.583 | 0.929 | 5.279 | -4.527 | 0.972 | 6.429 | -5.262 | 0.982 |
| NGRFG |  | 3.419 | -2.823 | 0.998 | 4.375 | -3.434 | 0.998 | 4.324 | -3.294 | 0.998 |
| PGRFG |  | 3.077 | -2.453 | 1.000 | 4.429 | -3.358 | 0.998 | 4.240 | -3.127 | 0.997 |
| QGRFG |  | 2.912 | -2.425 | 1.000 | 3.916 | -3.074 | 0.997 | 3.824 | -2.907 | 0.996 |
| RGRFG |  | 2.882 | -2.536 | 1.000 | 3.230 | -2.754 | 1.000 | 3.127 | -2.574 | 1.000 |
| SGRFG |  | 2.931 | -2.451 | 1.000 | 3.743 | -2.969 | 0.995 | 3.706 | -2.842 | 0.996 |
| TGRFG |  | 3.750 | -2.996 | 0.996 | 4.542 | -3.481 | 0.999 | 4.499 | -3.352 | 0.999 |
| VGRFG |  | 4.917 | -3.583 | 1.000 | 5.190 | -3.681 | 1.000 | 5.226 | -3.619 | 1.000 |
| NPNPC |  | 3.098 | -2.706 | 0.990 | 3.688 | -3.093 | 0.998 | 3.793 | -3.099 | 0.997 |
| NPNPI |  | 4.664 | -3.085 | 1.000 | 4.822 | -3.142 | 1.000 | 4.842 | -3.106 | 1.000 |
| NPNPM |  | 4.498 | -3.353 | 1.000 | 4.751 | -3.474 | 1.000 | 4.800 | -3.437 | 1.000 |
| NPNPP |  | 3.483 | -2.929 | 0.999 | 4.727 | -3.833 | 0.991 | 4.472 | -3.582 | 0.987 |
| NPNPV |  | 4.344 | -3.252 | 1.000 | 4.567 | -3.373 | 1.000 | 4.547 | -3.318 | 1.000 |
| NPNPY |  | 4.733 | -3.525 | 1.000 | 5.005 | -3.615 | 1.000 | 5.041 | -3.522 | 1.000 |
| CPNPT |  | 3.307 | -2.857 | 0.998 | 4.597 | -3.807 | 0.998 | 4.748 | -3.848 | 0.994 |
| IPNPT |  | 5.396 | -3.834 | 1.000 | 5.700 | -4.008 | 1.000 | 5.922 | -4.127 | 1.000 |
| LPNPT |  | 5.477 | -3.785 | 1.000 | 5.702 | -3.895 | 1.000 | 5.798 | -3.918 | 1.000 |
| MPNPT |  | 5.083 | -3.753 | 1.000 | 5.382 | -3.914 | 1.000 | 5.532 | -3.962 | 1.000 |
| QPNPT |  | 4.422 | -3.334 | 0.999 | 4.657 | -3.473 | 1.000 | 4.768 | -3.473 | 1.000 |
| VPNPT |  | 4.749 | -3.719 | 0.999 | 5.116 | -3.960 | 0.999 | 4.949 | -3.803 | 0.998 |
| YPNPT |  | 5.385 | -3.913 | 1.000 | 5.835 | -4.144 | 1.000 | 6.151 | -4.262 | 1.000 |
| APNPT | pH=5 | 4.986 | -4.378 | 0.992 | 5.988 | -5.237 | 0.996 | 5.094 | -4.419 | 0.980 |
| DPNPT |  | 8.924 | -8.003 | 0.908 | 9.263 | -8.256 | 0.910 | 3.177 | -2.810 | 0.988 |
| EPNPT |  | 7.996 | -7.167 | 0.927 | 7.509 | -6.781 | 0.926 | 8.174 | -7.376 | 0.928 |
| GPNPT |  | 4.148 | -3.711 | 0.987 | 4.187 | -3.707 | 0.988 | 4.314 | -3.780 | 0.979 |
| HPNPT |  | 8.773 | -7.608 | 0.943 | 8.441 | -7.257 | 0.923 | 9.937 | -8.541 | 0.929 |
| KPNPT |  | 3.284 | -3.263 | 0.998 | 3.447 | -3.400 | 0.997 | 3.401 | -3.359 | 0.994 |
| NPNPT |  | 5.555 | -4.981 | 0.997 | 5.896 | -5.242 | 0.998 | 6.218 | -5.464 | 0.998 |
| PPNPT |  | 6.146 | -5.245 | 0.997 | 6.510 | -5.529 | 0.998 | 6.834 | -5.768 | 0.995 |
| RPNPT |  | 3.791 | -3.608 | 0.999 | 3.998 | -3.751 | 0.999 | 4.227 | -3.917 | 0.999 |
| SPNPT |  | 5.465 | -4.901 | 0.998 | 5.750 | -5.124 | 0.997 | 6.088 | -5.372 | 0.998 |
| TPNPT |  | 5.763 | -4.954 | 0.996 | 6.477 | -5.549 | 0.999 | 6.208 | -5.263 | 0.994 |
| NPNPA |  | 4.444 | -3.960 | 0.991 | 5.415 | -4.784 | 0.994 | 5.050 | -4.417 | 0.994 |
| NPNPD |  | 7.217 | -6.649 | 0.936 | 4.199 | -4.147 | 0.990 | 8.142 | -7.451 | 0.825 |
| NPNPE |  | 6.937 | -6.400 | 0.868 | 5.210 | -4.954 | 0.987 | 8.309 | -7.520 | 0.922 |
| NPNPG |  | 5.231 | -4.815 | 0.997 | 5.530 | -5.041 | 0.998 | 5.812 | -5.224 | 0.997 |
| NPNPH |  | 4.633 | -4.429 | 0.971 | 5.750 | -5.373 | 0.938 | 3.788 | -3.609 | 0.996 |
| NPNPK |  | 5.839 | -5.621 | 0.978 | 5.784 | -5.573 | 0.944 | 5.645 | -5.412 | 0.971 |
| NPNPN |  | 5.077 | -4.799 | 0.993 | 5.574 | -5.204 | 0.997 | 5.897 | -5.429 | 0.996 |
| NPNPQ |  | 5.675 | -5.262 | 0.992 | 5.985 | -5.498 | 0.995 | 6.464 | -5.839 | 0.997 |
| NPNPR |  | 3.661 | -3.522 | 1.000 | 4.049 | -3.812 | 0.999 | 4.068 | -3.788 | 0.999 |
| NPNPS |  | 5.078 | -4.772 | 0.995 | 5.452 | -5.071 | 0.997 | 5.844 | -5.353 | 0.996 |
| HGRFA |  | 2.659 | -2.241 | 0.944 | 0.782 | -0.787 | 0.804 | 4.253 | -3.284 | 0.762 |
| HGRFD |  | 2.623 | -2.245 | 1.000 | 2.464 | -2.065 | 0.979 | 2.584 | -2.085 | 1.000 |
| HGRFE |  | 3.196 | -2.711 | 0.989 | 2.289 | -1.981 | 0.964 | 3.395 | -2.735 | 0.991 |
| HGRFG |  | 1.926 | -1.837 | 0.998 | 1.849 | -1.753 | 0.999 | 1.826 | -1.701 | 0.999 |
| HGRFH |  | 1.038 | -1.247 | 0.995 | 1.469 | -1.500 | 0.995 | 1.317 | -1.360 | 0.993 |
| HGRFK |  | 0.848 | -1.119 | 0.965 | 1.130 | -1.270 | 0.976 | 0.827 | -1.032 | 0.979 |
| HGRFN |  | 1.902 | -1.778 | 0.994 | 1.947 | -1.705 | 0.974 | 1.451 | -1.349 | 0.992 |
| HGRFQ |  | 2.028 | -1.848 | 0.999 | 1.975 | -1.697 | 0.977 | 1.623 | -1.448 | 0.993 |
| HGRFR |  | 0.886 | -1.127 | 0.992 | 1.296 | -1.354 | 0.969 | 0.885 | -1.034 | 0.986 |
| HGRFS |  | 2.015 | -1.831 | 0.995 | 2.001 | -1.720 | 0.991 | 1.521 | -1.375 | 0.995 |
| HGRFT |  | 1.441 | -1.352 | 0.973 | 1.148 | -1.030 | 0.944 | 0.908 | -0.881 | 0.962 |
| AGRFG |  | 2.438 | -2.054 | 0.998 | 2.337 | -1.896 | 0.990 | 3.212 | -2.466 | 0.941 |
| DGRFG |  | 3.635 | -2.760 | 1.000 | 3.771 | -2.768 | 1.000 | 3.918 | -2.769 | 1.000 |
| EGRFG |  | 3.631 | -2.745 | 0.999 | 3.735 | -2.728 | 1.000 | 3.943 | -2.774 | 0.999 |
| GGRFG |  | 2.613 | -2.206 | 1.000 | 2.612 | -2.134 | 1.000 | 2.576 | -2.040 | 0.999 |
| KGRFG |  | 2.578 | -2.365 | 1.000 | 2.536 | -2.254 | 1.000 | 2.423 | -2.113 | 1.000 |
| NGRFG |  | 3.199 | -2.595 | 1.000 | 3.255 | -2.540 | 1.000 | 3.776 | -2.835 | 0.989 |
| PGRFG |  | 2.830 | -2.281 | 0.989 | 2.768 | -2.141 | 0.975 | 5.191 | -3.803 | 0.951 |
| QGRFG |  | 2.430 | -2.062 | 0.999 | 2.583 | -2.069 | 0.998 | 3.227 | -2.477 | 0.958 |
| RGRFG |  | 2.482 | -2.223 | 1.000 | 2.461 | -2.132 | 1.000 | 2.117 | -1.825 | 0.984 |
| SGRFG |  | 2.553 | -2.166 | 1.000 | 2.846 | -2.289 | 0.998 | 3.366 | -2.598 | 0.988 |
| TGRFG |  | 3.081 | -2.471 | 0.999 | 3.172 | -2.434 | 0.999 | 4.177 | -3.077 | 0.961 |
| VGRFG |  | 7.114 | -5.169 | 0.979 | 6.448 | -4.600 | 0.973 | 6.740 | -4.689 | 0.974 |
| NPNPC |  | 2.959 | -2.615 | 0.993 | 3.149 | -2.731 | 0.996 | 3.513 | -2.946 | 1.000 |
| NPNPI |  | 4.209 | -2.835 | 1.000 | 4.225 | -2.809 | 1.000 | 4.298 | -2.823 | 1.000 |
| NPNPM |  | 4.238 | -3.222 | 1.000 | 4.345 | -3.256 | 0.999 | 4.483 | -3.294 | 1.000 |
| NPNPP |  | 4.050 | -3.535 | 0.998 | 4.270 | -3.696 | 0.997 | 4.642 | -3.935 | 0.999 |
| NPNPV |  | 4.137 | -3.167 | 0.999 | 4.154 | -3.162 | 0.998 | 4.282 | -3.222 | 0.999 |
| NPNPY |  | 4.652 | -3.519 | 0.994 | 4.837 | -3.574 | 0.999 | 5.109 | -3.641 | 0.988 |
| CPNPT |  | 4.862 | -4.043 | 0.972 | 3.952 | -3.401 | 0.998 | 4.142 | -3.547 | 1.000 |
| IPNPT |  | 5.366 | -3.826 | 0.874 | 5.083 | -3.628 | 1.000 | 5.233 | -3.717 | 0.999 |
| LPNPT |  | 4.992 | -3.484 | 1.000 | 5.024 | -3.478 | 0.999 | 5.282 | -3.637 | 1.000 |
| MPNPT |  | 4.519 | -3.383 | 0.998 | 4.705 | -3.486 | 0.999 | 4.816 | -3.527 | 0.999 |
| QPNPT |  | 2.836 | -2.230 | 1.000 | 13.611 | -10.268 | 0.868 | 2.685 | -2.044 | 1.000 |
| VPNPT |  | 4.486 | -3.560 | 0.999 | 4.826 | -3.813 | 0.996 | 5.338 | -4.164 | 0.982 |
| YPNPT |  | 5.031 | -3.686 | 1.000 | 5.171 | -3.712 | 0.999 | 5.783 | -4.071 | 0.999 |
| APNPT | pH=6 | 6.382 | -5.669 | 0.998 | 6.655 | -5.880 | 0.999 | 6.753 | -5.915 | 0.999 |
| DPNPT |  | 6.645 | -6.240 | 0.996 | 7.050 | -6.541 | 0.997 | 6.485 | -5.962 | 0.986 |
| EPNPT |  | 6.954 | -6.492 | 0.998 | 8.013 | -7.419 | 0.991 | 7.266 | -6.773 | 0.996 |
| GPNPT |  | 6.253 | -5.665 | 0.999 | 6.599 | -5.925 | 0.999 | 6.705 | -5.948 | 0.999 |
| HPNPT |  | 7.503 | -6.369 | 0.999 | 7.788 | -6.526 | 0.999 | 7.764 | -6.405 | 0.998 |
| KPNPT |  | 6.956 | -6.567 | 0.991 | 7.604 | -7.171 | 0.995 | 7.014 | -6.636 | 0.965 |
| NPNPT |  | 6.337 | -5.695 | 0.998 | 6.959 | -6.193 | 1.000 | 6.939 | -6.096 | 0.999 |
| PPNPT |  | 6.965 | -6.021 | 0.999 | 7.359 | -6.324 | 0.999 | 7.801 | -6.658 | 0.998 |
| RPNPT |  | 6.885 | -6.224 | 0.995 | 7.669 | -6.898 | 0.999 | 7.685 | -6.852 | 0.997 |
| SPNPT |  | 6.387 | -5.757 | 0.999 | 6.743 | -6.032 | 1.000 | 6.819 | -6.035 | 0.999 |
| TPNPT |  | 6.675 | -5.776 | 0.999 | 7.036 | -6.056 | 0.999 | 7.190 | -6.132 | 0.999 |
| NPNPA |  | 5.856 | -5.258 | 1.000 | 6.157 | -5.483 | 1.000 | 6.250 | -5.505 | 0.999 |
| NPNPD |  | 6.626 | -6.530 | 0.926 | 5.505 | -5.516 | 0.984 | 5.155 | -5.117 | 0.944 |
| NPNPE |  | 5.061 | -5.117 | 0.982 | 5.744 | -5.677 | 0.970 | 6.012 | -5.814 | 0.962 |
| NPNPG |  | 6.551 | -6.022 | 1.000 | 6.763 | -6.161 | 1.000 | 6.683 | -6.010 | 0.999 |
| NPNPH |  | 6.433 | -5.889 | 0.999 | 6.901 | -6.274 | 0.999 | 6.867 | -6.186 | 0.998 |
| NPNPK |  | 7.214 | -6.769 | 0.999 | 7.579 | -7.093 | 0.999 | 7.501 | -6.978 | 0.998 |
| NPNPN |  | 6.866 | -6.434 | 0.999 | 7.271 | -6.744 | 1.000 | 7.214 | -6.611 | 0.999 |
| NPNPQ |  | 7.148 | -6.611 | 0.997 | 7.355 | -6.739 | 0.999 | 7.335 | -6.633 | 1.000 |
| NPNPR |  | 7.282 | -6.600 | 0.999 | 7.750 | -6.969 | 0.999 | 7.714 | -6.855 | 0.998 |
| NPNPS |  | 6.530 | -6.102 | 0.998 | 7.043 | -6.510 | 1.000 | 6.916 | -6.319 | 0.999 |
| HGRFA |  | 4.288 | -2.986 | 1.000 | 4.245 | -2.904 | 0.999 | 5.462 | -3.749 | 0.998 |
| HGRFD |  | 4.750 | -3.714 | 0.997 | 4.912 | -3.739 | 1.000 | 5.295 | -3.895 | 1.000 |
| HGRFE |  | 4.803 | -3.755 | 0.997 | 4.796 | -3.658 | 1.000 | 5.470 | -4.038 | 0.999 |
| HGRFG |  | 3.949 | -2.857 | 0.995 | 8.290 | -6.024 | 0.868 | 5.656 | -4.007 | 0.997 |
| HGRFH |  | 6.350 | -4.688 | 0.952 | 5.660 | -4.134 | 0.996 | 7.224 | -5.283 | 1.000 |
| HGRFK |  | 4.937 | -3.976 | 0.980 | 4.653 | -3.723 | 1.000 | 5.318 | -4.163 | 0.999 |
| HGRFN |  | 5.673 | -4.320 | 0.994 | 5.496 | -4.137 | 0.999 | 4.873 | -3.522 | 0.997 |
| HGRFQ |  | 7.416 | -5.602 | 0.945 | 7.025 | -5.259 | 0.992 | 5.006 | -3.616 | 1.000 |
| HGRFR |  | 6.474 | -5.034 | 0.974 | 6.065 | -4.670 | 0.999 | 5.500 | -4.161 | 1.000 |
| HGRFS |  | 5.521 | -4.151 | 0.982 | 5.505 | -4.084 | 0.989 | 3.958 | -2.808 | 0.995 |
| HGRFT |  | 5.086 | -3.648 | 0.997 | 4.943 | -3.480 | 0.999 | 4.734 | -3.241 | 1.000 |
| AGRFG |  | 4.381 | -3.349 | 0.999 | 4.459 | -3.331 | 1.000 | 3.969 | -2.825 | 0.999 |
| DGRFG |  | 4.293 | -3.302 | 1.000 | 4.287 | -3.193 | 1.000 | 4.359 | -3.104 | 0.999 |
| EGRFG |  | 4.320 | -3.307 | 1.000 | 4.368 | -3.237 | 1.000 | 4.376 | -3.103 | 0.999 |
| GGRFG |  | 4.073 | -3.204 | 0.999 | 4.267 | -3.253 | 1.000 | 3.969 | -2.866 | 0.993 |
| KGRFG |  | 4.836 | -3.967 | 1.000 | 4.976 | -3.984 | 1.000 | 4.703 | -3.523 | 0.973 |
| NGRFG |  | 4.114 | -3.076 | 1.000 | 4.170 | -3.040 | 1.000 | 4.175 | -2.924 | 1.000 |
| PGRFG |  | 4.522 | -3.371 | 0.999 | 4.630 | -3.377 | 1.000 | 4.414 | -3.089 | 1.000 |
| QGRFG |  | 4.167 | -3.237 | 0.999 | 4.166 | -3.150 | 0.999 | 3.803 | -2.725 | 0.998 |
| RGRFG |  | 5.654 | -4.483 | 0.985 | 5.452 | -4.261 | 1.000 | 4.436 | -3.348 | 1.000 |
| SGRFG |  | 4.189 | -3.180 | 0.999 | 4.160 | -3.092 | 1.000 | 3.921 | -2.789 | 1.000 |
| TGRFG |  | 4.023 | -2.931 | 1.000 | 4.143 | -2.965 | 1.000 | 4.106 | -2.829 | 1.000 |
| VGRFG |  | 4.247 | -2.745 | 0.999 | 4.457 | -2.887 | 1.000 | 4.523 | -2.854 | 1.000 |
| NPNPC |  | 4.351 | -3.760 | 0.996 | 4.625 | -3.919 | 0.996 | 4.395 | -3.651 | 0.996 |
| NPNPI |  | 4.790 | -3.319 | 1.000 | 4.795 | -3.268 | 1.000 | 4.803 | -3.209 | 1.000 |
| NPNPM |  | 4.975 | -3.828 | 1.000 | 5.053 | -3.824 | 1.000 | 4.976 | -3.678 | 1.000 |
| NPNPP |  | 5.560 | -4.805 | 1.000 | 7.365 | -6.126 | 0.973 | 5.845 | -4.952 | 0.999 |
| NPNPV |  | 4.759 | -3.713 | 1.000 | 4.834 | -3.733 | 1.000 | 4.738 | -3.602 | 1.000 |
| NPNPY |  | 5.136 | -3.963 | 1.000 | 5.121 | -3.845 | 1.000 | 5.165 | -3.733 | 1.000 |
| CPNPT |  | 5.887 | -4.819 | 0.997 | 6.236 | -5.095 | 0.991 | 5.476 | -4.526 | 0.987 |
| IPNPT |  | 4.895 | -3.456 | 1.000 | 5.248 | -3.719 | 1.000 | 5.569 | -3.936 | 1.000 |
| LPNPT |  | 5.023 | -3.470 | 1.000 | 5.309 | -3.666 | 1.000 | 5.444 | -3.730 | 1.000 |
| MPNPT |  | 4.811 | -3.554 | 1.000 | 5.142 | -3.784 | 1.000 | 5.280 | -3.846 | 0.999 |
| QPNPT |  | 4.276 | -3.656 | 1.000 | 3.154 | -2.778 | 0.978 | 6.987 | -5.511 | 0.916 |
| VPNPT |  | 4.929 | -3.897 | 1.000 | 5.689 | -4.484 | 1.000 | 5.765 | -4.549 | 1.000 |
| YPNPT |  | 5.283 | -3.878 | 1.000 | 5.604 | -4.036 | 1.000 | 5.803 | -4.072 | 1.000 |
| APNPT | pH=7 | 5.046 | -4.322 | 0.997 | 5.434 | -4.640 | 0.997 | 5.416 | -4.590 | 0.996 |
| DPNPT |  | 12.195 | -11.336 | 0.883 | 9.077 | -8.511 | 1.000 | 7.627 | -7.178 | 1.000 |
| EPNPT |  | 10.761 | -10.089 | 0.944 | 9.907 | -9.272 | 0.999 | 8.962 | -8.419 | 0.993 |
| GPNPT |  | 4.534 | -3.975 | 0.996 | 5.012 | -4.360 | 0.996 | 4.995 | -4.291 | 0.996 |
| HPNPT |  | 6.600 | -5.438 | 0.999 | 7.029 | -5.714 | 0.998 | 7.116 | -5.665 | 0.995 |
| KPNPT |  | 4.047 | -3.314 | 0.972 | 4.545 | -3.738 | 0.982 | 4.433 | -3.593 | 0.968 |
| NPNPT |  | 4.898 | -4.447 | 0.983 | 6.148 | -5.479 | 0.995 | 6.358 | -5.573 | 0.998 |
| PPNPT |  | 5.307 | -4.325 | 0.995 | 5.775 | -4.702 | 0.995 | 5.940 | -4.799 | 0.995 |
| RPNPT |  | 5.297 | -4.232 | 0.995 | 5.621 | -4.468 | 0.999 | 5.373 | -4.167 | 0.995 |
| SPNPT |  | 5.152 | -4.618 | 0.994 | 5.894 | -5.211 | 0.996 | 5.851 | -5.092 | 0.996 |
| TPNPT |  | 6.435 | -5.529 | 0.989 | 6.859 | -5.838 | 0.997 | 6.718 | -5.651 | 0.998 |
| NPNPA |  | 4.112 | -3.743 | 0.915 | 4.866 | -4.347 | 0.995 | 5.308 | -4.659 | 0.997 |
| NPNPD |  | \ | \ | \ | \ | \ | \ | \ | \ | \ |
| NPNPE |  | \ | \ | \ | \ | \ | \ | \ | \ | \ |
| NPNPG |  | 5.343 | -4.939 | 0.980 | 5.402 | -4.931 | 0.984 | 5.714 | -5.116 | 0.995 |
| NPNPH |  | 5.464 | -4.810 | 0.996 | 6.093 | -5.273 | 0.995 | 5.929 | -4.997 | 0.998 |
| NPNPK |  | 4.249 | -3.682 | 0.994 | 4.639 | -4.005 | 0.994 | 4.582 | -3.871 | 0.995 |
| NPNPN |  | 5.875 | -5.494 | 0.993 | 6.234 | -5.747 | 0.987 | 5.171 | -4.724 | 0.978 |
| NPNPQ |  | 5.077 | -4.728 | 0.980 | 6.249 | -5.704 | 0.998 | 5.628 | -5.067 | 0.983 |
| NPNPR |  | 4.997 | -4.191 | 0.995 | 5.528 | -4.607 | 0.996 | 5.678 | -4.631 | 0.993 |
| NPNPS |  | 5.479 | -5.125 | 0.995 | 5.974 | -5.500 | 0.992 | 5.135 | -4.677 | 0.980 |
| HGRFA |  | 3.914 | -2.296 | 0.999 | 4.220 | -2.439 | 0.999 | 4.338 | -2.390 | 0.983 |
| HGRFD |  | 4.242 | -3.282 | 0.995 | 4.709 | -3.514 | 0.999 | 5.529 | -3.924 | 0.980 |
| HGRFE |  | 4.461 | -3.448 | 0.998 | 4.943 | -3.696 | 1.000 | 5.597 | -3.989 | 0.982 |
| HGRFG |  | 4.134 | -2.559 | 0.998 | 4.018 | -2.389 | 0.999 | 4.051 | -2.264 | 0.992 |
| HGRFH |  | 4.081 | -2.386 | 0.999 | 4.509 | -2.595 | 0.999 | 4.362 | -2.299 | 0.988 |
| HGRFK |  | 3.330 | -1.774 | 0.968 | 2.796 | -1.401 | 0.994 | 2.547 | -1.042 | 0.882 |
| HGRFN |  | 3.501 | -2.186 | 0.998 | 4.032 | -2.507 | 0.999 | 3.965 | -2.305 | 0.965 |
| HGRFQ |  | 3.615 | -2.240 | 0.999 | 4.225 | -2.617 | 0.998 | 4.160 | -2.407 | 0.980 |
| HGRFR |  | -1.685 | 1.892 | 0.195 | 2.872 | -1.296 | 0.997 | 2.945 | -1.170 | 0.915 |
| HGRFS |  | 3.461 | -2.124 | 1.000 | 4.032 | -2.471 | 0.999 | 3.976 | -2.277 | 0.986 |
| HGRFT |  | 3.843 | -2.281 | 1.000 | 4.309 | -2.546 | 0.999 | 4.295 | -2.389 | 0.990 |
| AGRFG |  | 3.017 | -1.775 | 0.998 | 3.248 | -1.882 | 1.000 | 3.146 | -1.708 | 0.984 |
| DGRFG |  | 4.014 | -3.129 | 0.997 | 4.489 | -3.358 | 0.999 | 4.359 | -3.099 | 1.000 |
| EGRFG |  | 4.077 | -3.117 | 1.000 | 4.201 | -3.095 | 1.000 | 4.276 | -2.986 | 0.997 |
| GGRFG |  | 2.662 | -1.613 | 0.994 | 2.959 | -1.758 | 1.000 | 2.903 | -1.580 | 0.991 |
| KGRFG |  | 1.110 | -0.074 | 0.978 | 2.248 | -0.859 | 0.998 | 1.471 | -0.148 | 0.949 |
| NGRFG |  | 3.361 | -2.253 | 0.999 | 3.682 | -2.417 | 1.000 | 3.782 | -2.367 | 0.997 |
| PGRFG |  | 2.736 | -1.282 | 0.999 | 3.089 | -1.513 | 0.999 | 3.068 | -1.433 | 0.975 |
| QGRFG |  | 2.576 | -1.510 | 0.994 | 2.933 | -1.700 | 1.000 | 4.572 | -2.836 | 0.993 |
| RGRFG |  | 2.656 | -1.571 | 0.997 | 2.966 | -1.730 | 1.000 | 2.935 | -1.592 | 0.989 |
| SGRFG |  | 3.179 | -2.084 | 0.999 | 0.825 | -0.262 | 0.118 | 3.548 | -2.158 | 0.995 |
| TGRFG |  | 3.524 | -2.229 | 0.998 | 3.822 | -2.369 | 0.999 | 3.881 | -2.301 | 0.998 |
| VGRFG |  | 4.260 | -2.251 | 1.000 | 4.383 | -2.285 | 1.000 | 4.351 | -2.199 | 0.997 |
| NPNPC |  | \ | \ | \ | \ | \ | \ | \ | \ | \ |
| NPNPI |  | 5.207 | -3.866 | 1.000 | 5.370 | -3.918 | 1.000 | 5.305 | -3.774 | 0.995 |
| NPNPM |  | 3.939 | -3.218 | 0.991 | 5.168 | -4.072 | 0.997 | 5.259 | -4.020 | 0.999 |
| NPNPP |  | 3.612 | -3.323 | 0.985 | 6.003 | -5.061 | 0.959 | 4.862 | -4.104 | 0.960 |
| NPNPV |  | 5.088 | -4.115 | 0.948 | 4.220 | -3.446 | 0.984 | 4.396 | -3.487 | 0.997 |
| NPNPY |  | 3.908 | -3.197 | 0.994 | 5.325 | -4.163 | 0.997 | 5.442 | -4.086 | 0.998 |
| CPNPT |  | 5.513 | -4.632 | 0.964 | 6.282 | -5.181 | 0.953 | 5.139 | -4.205 | 0.711 |
| IPNPT |  | 5.125 | -3.512 | 0.999 | 5.136 | -3.489 | 0.999 | 5.052 | -3.402 | 0.999 |
| LPNPT |  | 5.230 | -3.531 | 0.999 | 5.186 | -3.461 | 1.000 | 5.118 | -3.377 | 1.000 |
| MPNPT |  | 5.079 | -3.751 | 1.000 | 5.306 | -3.856 | 1.000 | 5.347 | -3.805 | 0.999 |
| QPNPT |  | 3.613 | -3.278 | 0.965 | 5.432 | -4.604 | 0.972 | 5.098 | -4.268 | 0.985 |
| VPNPT |  | 4.474 | -3.375 | 0.999 | 4.679 | -3.511 | 1.000 | 4.387 | -3.272 | 1.000 |
| YPNPT |  | 5.348 | -3.955 | 0.998 | 6.191 | -4.457 | 0.996 | 5.935 | -4.134 | 0.995 |
| APNPT | pH=8 | 6.029 | -5.088 | 0.997 | 6.685 | -5.616 | 0.998 | 7.023 | -5.921 | 0.999 |
| DPNPT |  | 8.279 | -7.790 | 0.895 | 8.057 | -7.504 | 0.997 | 8.634 | -8.007 | 0.983 |
| EPNPT |  | 10.374 | -9.671 | 0.978 | 8.009 | -7.498 | 0.986 | 7.991 | -7.555 | 0.974 |
| GPNPT |  | 5.589 | -4.892 | 0.997 | 6.468 | -5.631 | 0.999 | 6.835 | -5.940 | 0.999 |
| HPNPT |  | 7.108 | -5.796 | 0.998 | 7.777 | -6.230 | 0.998 | 8.183 | -6.497 | 0.998 |
| KPNPT |  | 5.396 | -4.621 | 0.993 | 6.802 | -5.908 | 0.999 | 7.264 | -6.292 | 1.000 |
| NPNPT |  | 6.215 | -5.497 | 0.999 | 7.147 | -6.229 | 0.999 | 7.457 | -6.449 | 0.998 |
| PPNPT |  | 6.150 | -4.808 | 0.992 | 6.414 | -5.086 | 0.997 | 6.509 | -5.191 | 0.996 |
| RPNPT |  | 6.282 | -5.209 | 0.998 | 7.389 | -6.165 | 0.998 | 7.818 | -6.452 | 0.998 |
| SPNPT |  | 6.096 | -5.367 | 1.000 | 6.845 | -5.952 | 0.999 | 7.138 | -6.175 | 0.999 |
| TPNPT |  | 6.396 | -5.391 | 0.998 | 7.248 | -6.053 | 0.998 | 7.565 | -6.307 | 0.998 |
| NPNPA |  | 5.635 | -4.986 | 0.999 | 6.717 | -5.860 | 0.999 | 6.797 | -5.893 | 0.998 |
| NPNPD |  | \ | \ | \ | \ | \ | \ | \ | \ | \ |
| NPNPE |  | \ | \ | \ | 20.122 | -18.371 | 0.786 | \ | \ | \ |
| NPNPG |  | 6.030 | -5.454 | 0.995 | 7.073 | -6.291 | 1.000 | 7.346 | -6.476 | 1.000 |
| NPNPH |  | 6.223 | -5.462 | 0.997 | 7.147 | -6.165 | 0.999 | 7.363 | -6.277 | 0.998 |
| NPNPK |  | 5.337 | -4.797 | 0.997 | 6.938 | -6.232 | 0.998 | 7.056 | -6.267 | 0.998 |
| NPNPN |  | 6.032 | -5.551 | 0.991 | 7.419 | -6.696 | 1.000 | 7.933 | -7.093 | 1.000 |
| NPNPQ |  | 6.483 | -5.887 | 0.998 | 7.678 | -6.856 | 1.000 | 7.859 | -6.955 | 1.000 |
| NPNPR |  | 5.849 | -5.078 | 0.995 | 7.321 | -6.344 | 0.999 | 7.594 | -6.480 | 0.998 |
| NPNPS |  | 5.962 | -5.462 | 0.995 | 7.267 | -6.537 | 1.000 | 7.620 | -6.794 | 1.000 |
| HGRFA |  | 4.591 | -2.870 | 0.998 | 4.921 | -3.060 | 0.999 | 4.997 | -2.995 | 0.999 |
| HGRFD |  | 4.826 | -3.629 | 0.996 | 4.635 | -3.399 | 0.998 | 5.163 | -3.686 | 0.999 |
| HGRFE |  | 4.409 | -3.308 | 1.000 | 4.941 | -3.616 | 0.998 | 5.263 | -3.766 | 0.999 |
| HGRFG |  | 4.420 | -2.847 | 0.999 | 4.826 | -3.099 | 1.000 | 4.978 | -3.083 | 0.999 |
| HGRFH |  | 4.928 | -3.123 | 0.999 | 5.453 | -3.434 | 0.999 | 5.651 | -3.422 | 0.998 |
| HGRFK |  | 3.694 | -2.204 | 0.998 | 3.872 | -2.599 | 0.998 | 4.140 | -2.691 | 0.999 |
| HGRFN |  | 4.236 | -2.815 | 0.999 | 4.803 | -3.211 | 0.999 | 4.904 | -3.155 | 0.999 |
| HGRFQ |  | 4.472 | -2.980 | 0.998 | 5.042 | -3.365 | 0.999 | 5.178 | -3.332 | 0.999 |
| HGRFR |  | 3.005 | -1.748 | 0.992 | 4.530 | -2.967 | 0.999 | 4.712 | -2.961 | 0.998 |
| HGRFS |  | 4.179 | -2.745 | 0.999 | 4.742 | -3.129 | 1.000 | 4.848 | -3.080 | 0.999 |
| HGRFT |  | 4.550 | -2.885 | 0.999 | 4.941 | -3.129 | 1.000 | 5.041 | -3.077 | 0.999 |
| AGRFG |  | 4.093 | -2.694 | 0.999 | 4.241 | -2.771 | 1.000 | 4.159 | -2.624 | 0.999 |
| DGRFG |  | 5.132 | -3.928 | 0.999 | 4.878 | -3.600 | 0.999 | 5.004 | -3.594 | 0.999 |
| EGRFG |  | 4.973 | -3.816 | 0.999 | 4.881 | -3.617 | 1.000 | 5.053 | -3.645 | 1.000 |
| GGRFG |  | 4.093 | -2.846 | 0.998 | 3.958 | -2.716 | 1.000 | 4.048 | -2.683 | 1.000 |
| KGRFG |  | 2.155 | -1.005 | 1.000 | 2.997 | -2.006 | 1.000 | 3.222 | -2.075 | 0.997 |
| NGRFG |  | 3.799 | -2.655 | 0.997 | 4.290 | -3.006 | 1.000 | 4.423 | -2.977 | 1.000 |
| PGRFG |  | 4.393 | -2.490 | 0.998 | 4.396 | -2.489 | 1.000 | 5.248 | -3.093 | 0.714 |
| QGRFG |  | 3.974 | -2.673 | 0.997 | 3.680 | -2.458 | 0.998 | 3.913 | -2.535 | 1.000 |
| RGRFG |  | 3.360 | -2.239 | 1.000 | 3.507 | -2.344 | 0.999 | 3.881 | -2.510 | 1.000 |
| SGRFG |  | 3.778 | -2.647 | 0.998 | 4.107 | -2.844 | 1.000 | 4.202 | -2.806 | 1.000 |
| TGRFG |  | 4.087 | -2.737 | 0.999 | 4.363 | -2.887 | 1.000 | 4.331 | -2.763 | 0.999 |
| VGRFG |  | 5.118 | -2.897 | 0.999 | 5.257 | -2.932 | 1.000 | 5.254 | -2.841 | 0.999 |
| NPNPC |  | 3.450 | -3.057 | 0.996 | 5.702 | -4.690 | 0.998 | 5.901 | -4.806 | 0.999 |
| NPNPI |  | 4.930 | -3.542 | 1.000 | 5.522 | -3.853 | 0.999 | 5.699 | -3.968 | 0.999 |
| NPNPM |  | 5.950 | -4.593 | 0.998 | 5.892 | -4.445 | 1.000 | 6.462 | -4.823 | 1.000 |
| NPNPP |  | 4.316 | -3.783 | 0.977 | 7.550 | -6.215 | 0.996 | 7.417 | -6.126 | 0.980 |
| NPNPV |  | 5.583 | -4.390 | 0.998 | 5.767 | -4.453 | 1.000 | 6.343 | -4.877 | 1.000 |
| NPNPY |  | 6.123 | -4.753 | 0.997 | 6.039 | -4.547 | 1.000 | 6.389 | -4.712 | 0.999 |
| CPNPT |  | 8.572 | -6.745 | 0.981 | 7.162 | -5.489 | 0.986 | 7.031 | -5.461 | 0.981 |
| IPNPT |  | 6.111 | -4.011 | 0.997 | 5.977 | -3.818 | 0.999 | 5.939 | -3.811 | 0.998 |
| LPNPT |  | 6.842 | -4.544 | 0.830 | 5.912 | -3.730 | 0.999 | 5.804 | -3.675 | 0.999 |
| MPNPT |  | 5.974 | -4.238 | 0.998 | 5.878 | -4.062 | 0.999 | 6.058 | -4.178 | 0.999 |
| QPNPT |  | 4.370 | -3.744 | 0.973 | 6.534 | -5.367 | 0.990 | 6.839 | -5.613 | 0.983 |
| VPNPT |  | 5.802 | -4.173 | 0.999 | 5.792 | -4.094 | 0.999 | 6.006 | -4.279 | 0.999 |
| YPNPT |  | 6.291 | -4.531 | 0.999 | 6.229 | -4.347 | 0.999 | 6.455 | -4.441 | 0.999 |

**Supplementary Table 3.** Correlation Coefficients (R^2^) of $\mathrm{pKa}$, $\log k_{\mathrm{HA}}$, and $\log k_{A}$ with respect to $1/T$.

| **Correlation Coefficients-R^2^** | | | | | |
| --- | --- | --- | --- | --- | --- |
| Mobile phase compositions | Pentapeptide sequence | Acid-base property | $pK_{a}\sim1/T$ | $\log k_{\mathrm{HA}}\sim1/T$ | $\log k_{A}\sim1/T$ |
| 8% | DPNPT | acidity | 1 | 0.9714 | 0.9987 |
| 8% | EPNPT | acidity | 0.75 | 0.9633 | 0.9987 |
| 8% | NPNPD | acidity | 0 | 0.9223 | 0.75 |
| 8% | NPNPE | acidity | 0 | 0.9404 | 0.75 |
| 10% | DPNPT | acidity | 0.75 | 0.9975 | 0.75 |
| 10% | EPNPT | acidity | 0.75 | 0.9994 | 1 |
| 10% | NPNPD | acidity | 1 | 0.9954 | 0.75 |
| 10% | NPNPE | acidity | 1 | 0.9901 | 0.9905 |
| 12% | DPNPT | acidity | 0.9643 | 0.984 | 0.75 |
| 12% | EPNPT | acidity | 1 | 0.9968 | 1 |
| 12% | NPNPD | acidity | 0.75 | 0.9923 | 0.75 |
| 12% | NPNPE | acidity | 1 | 0.9947 | 0.75 |
| 14% | DPNPT | acidity | 0.9643 | 0.9992 | 0.9735 |
| 14% | EPNPT | acidity | 0.75 | 0.9996 | 0.75 |
| 14% | NPNPD | acidity | 0.1071 | 0.9743 | 1 |
| 14% | NPNPE | acidity | 0.75 | 0.9975 | / |
| 8% | APNPT | neuter | 0 | 0.9692 | 0.9557 |
| 8% | GPNPT | neuter | 0.8929 | 0.6183 | 0.9528 |
| 8% | HPNPT | neuter | / | 0.927 | 0.9994 |
| 8% | NPNPT | neuter | 0.3553 | 0.0919 | 0.998 |
| 8% | PPNPT | neuter | / | 0.7751 | 0.9917 |
| 8% | SPNPT | neuter | 0.75 | 0.626 | 0.998 |
| 8% | TPNPT | neuter | 0.75 | 0.7659 | 0.9984 |
| 8% | NPNPA | neuter | 0.5192 | 0.1757 | 0.998 |
| 8% | NPNPG | neuter | 1 | 0.4397 | 0.9779 |
| 8% | NPNPH | neuter | 1 | 0.8257 | 0.9447 |
| 8% | NPNPN | neuter | 1 | 0.8684 | 0.9812 |
| 8% | NPNPQ | neuter | 0.0577 | 0.1861 | 0.9471 |
| 8% | NPNPS | neuter | 0.9231 | 0.9977 | 0.9987 |
| 10% | APNPT | neuter | / | 0.6818 | 0.75 |
| 10% | GPNPT | neuter | 0.1579 | 0.3859 | 0.999 |
| 10% | HPNPT | neuter | 0.75 | 0.5764 | 0.9994 |
| 10% | NPNPT | neuter | 0.5714 | 0.7967 | 0.9471 |
| 10% | PPNPT | neuter | 0 | 0.8999 | 0.9712 |
| 10% | SPNPT | neuter | 1 | 0.9592 | 0.999 |
| 10% | TPNPT | neuter | 0.75 | 0.7629 | 0.9545 |
| 10% | NPNPA | neuter | 0.75 | 0.7364 | 0.999 |
| 10% | NPNPG | neuter | 0.75 | 0.702 | 0.75 |
| 10% | NPNPH | neuter | 0.75 | 0.9968 | 0.9835 |
| 10% | NPNPN | neuter | 0.75 | 0.7622 | 0.9966 |
| 10% | NPNPQ | neuter | 0.7874 | 0.8458 | 0.9977 |
| 10% | NPNPS | neuter | 0.7915 | 0.845 | 0.75 |
| 12% | APNPT | neuter | 1 | 0.9307 | 0.75 |
| 12% | GPNPT | neuter | 0.75 | 0.7727 | 0.9983 |
| 12% | HPNPT | neuter | 1 | 0.9503 | 0.9972 |
| 12% | NPNPT | neuter | 0.6823 | 0.6939 | 0.9977 |
| 12% | PPNPT | neuter | 0.871 | 0.8999 | 0.75 |
| 12% | SPNPT | neuter | 0.5052 | 0.5476 | 0.75 |
| 12% | TPNPT | neuter | / | 0.7275 | 0.9992 |
| 12% | NPNPA | neuter | 0.75 | 0.7748 | 0.75 |
| 12% | NPNPG | neuter | 0.9959 | 0.9626 | 0.75 |
| 12% | NPNPH | neuter | 0.9643 | 0.9469 | 0.75 |
| 12% | NPNPN | neuter | 0.75 | 0.7299 | 0.75 |
| 12% | NPNPQ | neuter | 0.812 | 0.6585 | 0.75 |
| 12% | NPNPS | neuter | 0.1071 | 0.0006 | 0.75 |
| 14% | APNPT | neuter | 0.706 | 0.6963 | 0.75 |
| 14% | GPNPT | neuter | 0.8027 | 0.7996 | 0.75 |
| 14% | HPNPT | neuter | 0.6279 | 0.5579 | 0.9947 |
| 14% | NPNPT | neuter | 0.6515 | 0.7171 | 0.75 |
| 14% | PPNPT | neuter | 0.75 | 0.9897 | 0 |
| 14% | SPNPT | neuter | 0.865 | 0.822 | 0.75 |
| 14% | TPNPT | neuter | 0 | 0.3204 | 0.75 |
| 14% | NPNPA | neuter | 0.784 | 0.7857 | 0.75 |
| 14% | NPNPG | neuter | 0.75 | 0.7589 | 0.75 |
| 14% | NPNPH | neuter | 1 | 0.9734 | 0.75 |
| 14% | NPNPN | neuter | 0.9973 | 0.9917 | 0.75 |
| 14% | NPNPQ | neuter | 0.75 | 0.75 | 1 |
| 14% | NPNPS | neuter | 0.75 | 0.7399 | 1 |
| 20% | HGRFD | neuter | / | 0.9994 | 0.9218 |
| 20% | HGRFE | neuter | / | 0.9991 | 0.9472 |
| 20% | DGRFG | neuter | / | 0.992 | 0.9849 |
| 20% | EGRFG | neuter | / | 0.9869 | 0.9999 |
| 20% | NPNPC | neuter | 0.1071 | 0.1098 | 0.75 |
| 20% | NPNPI | neuter | 0 | 0.0282 | 0.9986 |
| 20% | NPNPM | neuter | 0.75 | 0.3976 | 0.9767 |
| 20% | NPNPP | neuter | 0.75 | 0.98 | 0.75 |
| 20% | NPNPV | neuter | 1 | 0.9617 | 0.999 |
| 20% | NPNPY | neuter | 0.0357 | 0.1236 | 0.9849 |
| 20% | CPNPT | neuter | 0.0577 | 0.0186 | 1 |
| 20% | IPNPT | neuter | 0 | 0.8787 | 0.9139 |
| 20% | LPNPT | neuter | / | 0.8794 | 0.8838 |
| 20% | MPNPT | neuter | 0.75 | 0.9936 | 0.9985 |
| 20% | QPNPT | neuter | 0.1071 | 0.908 | 0.75 |
| 20% | VPNPT | neuter | 1 | 0.6981 | 0.75 |
| 20% | YPNPT | neuter | 0.75 | 0.5378 | 0.9999 |
| 22% | HGRFD | neuter | / | 0.9998 | 0.9812 |
| 22% | HGRFE | neuter | / | 0.9999 | 0.9812 |
| 22% | DGRFG | neuter | / | 0.9616 | 0.9985 |
| 22% | EGRFG | neuter | / | 0.9751 | 0.9985 |
| 22% | NPNPC | neuter | 0.0357 | 0.1325 | 0.75 |
| 22% | NPNPI | neuter | / | 0.0788 | 0.9767 |
| 22% | NPNPM | neuter | 0.5192 | 0.664 | 0.9794 |
| 22% | NPNPP | neuter | 0.8421 | 0.3344 | 1 |
| 22% | NPNPV | neuter | 0.75 | 0.7791 | 0.9983 |
| 22% | NPNPY | neuter | 1 | 0.982 | 0.9958 |
| 22% | CPNPT | neuter | 0.9735 | 0.7835 | 0.75 |
| 22% | IPNPT | neuter | / | 0.882 | 0.9997 |
| 22% | LPNPT | neuter | / | 0.7404 | 0.913 |
| 22% | MPNPT | neuter | 1 | 0.8037 | 0.9757 |
| 22% | QPNPT | neuter | 0.25 | 0.9748 | 0 |
| 22% | VPNPT | neuter | 1 | 0.1179 | 0.75 |
| 22% | YPNPT | neuter | 0.75 | 0.9497 | 1 |
| 24% | HGRFD | neuter | 0.75 | 0.9356 | 0.9977 |
| 24% | HGRFE | neuter | 0.75 | 0.8996 | 0.75 |
| 24% | DGRFG | neuter | 0.75 | 0.6595 | 0.9502 |
| 24% | EGRFG | neuter | / | 0.9187 | 0.9997 |
| 24% | NPNPC | neuter | 0.9796 | 1 | 0.75 |
| 24% | NPNPI | neuter | 0.8421 | 0.8089 | 0.9983 |
| 24% | NPNPM | neuter | 0.7002 | 0.7249 | 0.9977 |
| 24% | NPNPP | neuter | 0.5714 | 0.75 | 1 |
| 24% | NPNPV | neuter | 0.75 | 0.7793 | 0.75 |
| 24% | NPNPY | neuter | 0.5714 | 0.5344 | 0.9931 |
| 24% | CPNPT | neuter | 0.9643 | 0.6146 | 0.75 |
| 24% | IPNPT | neuter | 0 | 0.9048 | 0.75 |
| 24% | LPNPT | neuter | 0.75 | 0.9092 | 0.9551 |
| 24% | MPNPT | neuter | 0.75 | 0.6398 | 0.999 |
| 24% | QPNPT | neuter | 0 | 0.9992 | 1 |
| 24% | VPNPT | neuter | 0.75 | 0.8342 | 0.75 |
| 24% | YPNPT | neuter | 0.75 | 0.5425 | 0.9966 |
| 26% | HGRFD | neuter | 0.25 | 0.8352 | 0.9966 |
| 26% | HGRFE | neuter | 0.4286 | 0.8216 | 0.75 |
| 26% | DGRFG | neuter | 0.0448 | 0.1061 | 0.9835 |
| 26% | EGRFG | neuter | 0.0174 | 0 | 0.9905 |
| 26% | NPNPC | neuter | 0.0577 | 0.6518 | 1 |
| 26% | NPNPI | neuter | 1 | 0.9812 | 0.9993 |
| 26% | NPNPM | neuter | 0.75 | 0.7754 | 0.9966 |
| 26% | NPNPP | neuter | 0.9643 | 0.7872 | 0 |
| 26% | NPNPV | neuter | 0.8176 | 0.7243 | 0.75 |
| 26% | NPNPY | neuter | 0.75 | 0.9772 | 0.9865 |
| 26% | CPNPT | neuter | 0.9356 | 0.75 | 1 |
| 26% | IPNPT | neuter | 0.75 | 0.7022 | 0.75 |
| 26% | LPNPT | neuter | 1 | 0.9242 | 0.75 |
| 26% | MPNPT | neuter | 0.75 | 0.922 | 0.9983 |
| 26% | QPNPT | neuter | 0.0056 | 0.9987 | 0.75 |
| 26% | VPNPT | neuter | 0.5192 | 0.4549 | 0.75 |
| 26% | YPNPT | neuter | 0.4808 | 0.349 | 0.9812 |
| 8% | KPNPT | basicity | / | 0.9042 | 0 |
| 8% | RPNPT | basicity | 0.75 | 0.6465 | 0.9357 |
| 8% | NPNPK | basicity | 0.75 | 0.7833 | 0.9983 |
| 8% | NPNPR | basicity | / | 0.9171 | 0.9411 |
| 10% | KPNPT | basicity | 0.75 | 0.4746 | 1 |
| 10% | RPNPT | basicity | / | 0.9739 | 0.9757 |
| 10% | NPNPK | basicity | 0.6048 | 0.6414 | 0.75 |
| 10% | NPNPR | basicity | 0.8421 | 0.7245 | 0.75 |
| 12% | KPNPT | basicity | 0.0242 | 0.0739 | 0.75 |
| 12% | RPNPT | basicity | / | 0.9615 | 0.9794 |
| 12% | NPNPK | basicity | 0.75 | 0.7408 | 0.75 |
| 12% | NPNPR | basicity | 0.75 | 0.853 | 0.75 |
| 14% | KPNPT | basicity | 0.0854 | 0.0308 | 1 |
| 14% | RPNPT | basicity | 0.75 | 0.6199 | 0.2094 |
| 14% | NPNPK | basicity | 1 | 0.8746 | 0.75 |
| 14% | NPNPR | basicity | 0.25 | 0.4672 | 0.75 |
| 20% | HGRFA | basicity | 0.75 | 0.8015 | 0.3098 |
| 20% | HGRFG | basicity | 0.75 | 0.8024 | 0.2765 |
| 20% | HGRFH | basicity | / | 0.9701 | 0.9723 |
| 20% | HGRFK | basicity | 0.75 | 0.9303 | 0.0195 |
| 20% | HGRFN | basicity | / | 0.9997 | 0.9078 |
| 20% | HGRFQ | basicity | 0.75 | 0.8295 | 0.5088 |
| 20% | HGRFR | basicity | 0.5192 | 0.9978 | 0.9817 |
| 20% | HGRFS | basicity | 0.7371 | 0.7684 | 0.2647 |
| 20% | HGRFT | basicity | 0.75 | 0.8113 | 0.3389 |
| 20% | AGRFG | basicity | 0.75 | 0.9004 | 0.9626 |
| 20% | GGRFG | basicity | 0.75 | 0.8295 | 0.9823 |
| 20% | KGRFG | basicity | 0.75 | 0.7977 | 0.5069 |
| 20% | NGRFG | basicity | 0.75 | 0.8929 | 0.9835 |
| 20% | PGRFG | basicity | / | 0.9905 | 0.75 |
| 20% | QGRFG | basicity | 0.75 | 0.9213 | 0.9812 |
| 20% | RGRFG | basicity | 1 | 0.9755 | 0.9127 |
| 20% | SGRFG | basicity | 0.75 | 0.7985 | 0.9835 |
| 20% | TGRFG | basicity | 0.75 | 0.8498 | 0.994 |
| 20% | VGRFG | basicity | 0.75 | 0.9994 | 0.9815 |
| 22% | HGRFA | basicity | 0.7627 | 0.8366 | 0.3931 |
| 22% | HGRFG | basicity | 0.7373 | 0.7536 | 0.2603 |
| 22% | HGRFH | basicity | / | 0.9947 | 0.9795 |
| 22% | HGRFK | basicity | 0.75 | 0.9594 | 0 |
| 22% | HGRFN | basicity | / | 0.9878 | 0.9012 |
| 22% | HGRFQ | basicity | 0.75 | 0.9992 | 0.9352 |
| 22% | HGRFR | basicity | / | 0.9623 | 0.6916 |
| 22% | HGRFS | basicity | 0.7373 | 0.7664 | 0.3641 |
| 22% | HGRFT | basicity | 0.75 | 0.812 | 0.3676 |
| 22% | AGRFG | basicity | 0.75 | 0.8271 | 0.9823 |
| 22% | GGRFG | basicity | 0.75 | 0.6077 | 0.9972 |
| 22% | KGRFG | basicity | 0.9643 | 0.9861 | 0.0043 |
| 22% | NGRFG | basicity | 1 | 0.9978 | 0.9427 |
| 22% | PGRFG | basicity | / | 0.9978 | 0.847 |
| 22% | QGRFG | basicity | 1 | 0.9899 | 0.9966 |
| 22% | RGRFG | basicity | 0.75 | 0.9184 | 0.9972 |
| 22% | SGRFG | basicity | 1 | 0.9988 | 0.9427 |
| 22% | TGRFG | basicity | 0.75 | 0.8139 | 0.9823 |
| 22% | VGRFG | basicity | 1 | 0.9999 | 0.9963 |
| 24% | HGRFA | basicity | 0.75 | 0.9727 | 0.954 |
| 24% | HGRFG | basicity | 0.7375 | 0.7528 | 0.8543 |
| 24% | HGRFH | basicity | 0.75 | 0.9835 | 0.9542 |
| 24% | HGRFK | basicity | 0.75 | 0.937 | 0.0527 |
| 24% | HGRFN | basicity | / | 0.9905 | 0.8472 |
| 24% | HGRFQ | basicity | 1 | 0.9997 | 0.8388 |
| 24% | HGRFR | basicity | 0.75 | 0.9905 | 0.6165 |
| 24% | HGRFS | basicity | 0 | 0.0144 | 0.1095 |
| 24% | HGRFT | basicity | 0.75 | 0.7776 | 0.3065 |
| 24% | AGRFG | basicity | 0.75 | 0.7863 | 0.3849 |
| 24% | GGRFG | basicity | 0.6279 | 0.4854 | 0.9447 |
| 24% | KGRFG | basicity | 0.75 | 0.9839 | 0 |
| 24% | NGRFG | basicity | 0.75 | 0.6611 | 0.9472 |
| 24% | PGRFG | basicity | 0.75 | 0.8276 | 0.7338 |
| 24% | QGRFG | basicity | 1 | 0.9986 | 0.9767 |
| 24% | RGRFG | basicity | 0.75 | 0.6487 | 0.9447 |
| 24% | SGRFG | basicity | 0.75 | 0.6694 | 0.9472 |
| 24% | TGRFG | basicity | 0.75 | 0.654 | 0.9972 |
| 24% | VGRFG | basicity | 1 | 0.9923 | 0.9902 |
| 26% | HGRFA | basicity | 0.75 | 0.9663 | 0.9352 |
| 26% | HGRFG | basicity | 0 | 0.0147 | 0.1273 |
| 26% | HGRFH | basicity | 0 | 0.9966 | 0.8985 |
| 26% | HGRFK | basicity | 0.75 | 0.75 | 0.0505 |
| 26% | HGRFN | basicity | 0 | 0.9861 | 0.75 |
| 26% | HGRFQ | basicity | 0.0002 | 0.0169 | 0.1042 |
| 26% | HGRFR | basicity | 0.75 | 0.9303 | 0.473 |
| 26% | HGRFS | basicity | 0.0002 | 0.0164 | 0.1107 |
| 26% | HGRFT | basicity | 0 | 0.0241 | 0.1332 |
| 26% | AGRFG | basicity | 0.6663 | 0.4885 | 0.3389 |
| 26% | GGRFG | basicity | 1 | 0.9757 | 0.9472 |
| 26% | KGRFG | basicity | 0.75 | 0.8811 | 0 |
| 26% | NGRFG | basicity | 1 | 0.9978 | 0.9812 |
| 26% | PGRFG | basicity | 0 | 0.9923 | 0.6352 |
| 26% | QGRFG | basicity | 0.75 | 0.5598 | 0.75 |
| 26% | RGRFG | basicity | 0.75 | 0.5506 | 0.9794 |
| 26% | SGRFG | basicity | 0.6279 | 0.5224 | 0.9447 |
| 26% | TGRFG | basicity | 0.6279 | 0.4399 | 0.9447 |
| 26% | VGRFG | basicity | 0.75 | 0.979 | 0.9773 |

**Supplementary Table 4.** Correlation Coefficients (R^2^) of $\mathrm{pKa}$, $\log k_{\mathrm{HA}}$, and $\log k_{A}$ with respect to φ or $P_{m}^{N}$.

| Correlation Coefficients-R^2^ | | | | | | | | |
| --- | --- | --- | --- | --- | --- | --- | --- | --- |
| Temperatures | Pentapeptide sequence | Acid-base property | $\mathrm{pKa}$~φ | $\mathrm{pKa}$~$P_{m}^{N}$ | $\log k_{\mathrm{HA}}$~φ | $\log k_{\mathrm{HA}}$~$P_{m}^{N}$ | $\log k_{A}$~φ | $\log k_{A}\sim P_{m}^{N}$ |
| 25℃ | DPNPT | acidity | 0.6 | 0.6 | 0.9974 | 0.9974 | 0.9667 | 0.9667 |
| 25℃ | EPNPT | acidity | 0.9 | 0.9 | 0.9961 | 0.9961 | 0.9934 | 0.9934 |
| 25℃ | NPNPD | acidity | 0.9657 | 0.9657 | 0.9961 | 0.9961 | 0.9884 | 0.9884 |
| 25℃ | NPNPE | acidity | 0.6 | 0.6 | 0.9986 | 0.9986 | 0.9957 | 0.9957 |
| 25℃ | KPNPT | basicity | 0.0667 | 0.0667 | 0.0994 | 0.0994 | 0.9748 | 0.9748 |
| 25℃ | RPNPT | basicity | 0.6 | 0.6 | 0.9954 | 0.9954 | 0.9956 | 0.9956 |
| 25℃ | NPNPK | basicity | 0.4566 | 0.4566 | 0.0347 | 0.0347 | 0.9744 | 0.9744 |
| 25℃ | NPNPR | basicity | 0.741 | 0.741 | 0.2059 | 0.2059 | 0.9999 | 0.9999 |
| 25℃ | APNPT | neuter | 0.7909 | 0.7909 | 0.9428 | 0.9428 | 0.9873 | 0.9873 |
| 25℃ | GPNPT | neuter | 0.3959 | 0.3959 | 0.1518 | 0.1518 | 0.9943 | 0.9943 |
| 25℃ | HPNPT | neuter | 0.8 | 0.8 | 0.985 | 0.985 | 0.9937 | 0.9937 |
| 25℃ | NPNPT | neuter | 0.5 | 0.5 | 0.1302 | 0.1302 | 0.9982 | 0.9982 |
| 25℃ | PPNPT | neuter | 0.0034 | 0.0034 | 0.7103 | 0.7103 | 0.9975 | 0.9975 |
| 25℃ | SPNPT | neuter | 0.6982 | 0.6982 | 0.3817 | 0.3817 | 0.9982 | 0.9982 |
| 25℃ | TPNPT | neuter | 0.2286 | 0.2286 | 0.8392 | 0.8392 | 0.9943 | 0.9943 |
| 25℃ | NPNPA | neuter | 0.3959 | 0.3959 | 0.1435 | 0.1435 | 0.966 | 0.966 |
| 25℃ | NPNPG | neuter | 0.3521 | 0.3521 | 0.4473 | 0.4473 | 0.9946 | 0.9946 |
| 25℃ | NPNPH | neuter | 0.8333 | 0.8333 | 0.5058 | 0.5058 | 0.9957 | 0.9957 |
| 25℃ | NPNPN | neuter | 0.9761 | 0.9761 | 0.9887 | 0.9887 | 0.994 | 0.994 |
| 25℃ | NPNPQ | neuter | 0.4663 | 0.4663 | 0.4478 | 0.4478 | 0.9901 | 0.9901 |
| 25℃ | NPNPS | neuter | 0.445 | 0.445 | 0.4355 | 0.4355 | 0.994 | 0.994 |
| 25℃ | HGRFA | basicity | 0.7998 | 0.7998 | 0.8382 | 0.8382 | 0.2747 | 0.2747 |
| 25℃ | HGRFG | basicity | 0.8 | 0.8 | 0.8542 | 0.8542 | 0.2904 | 0.2904 |
| 25℃ | HGRFH | basicity | 0.8 | 0.8 | 0.9943 | 0.9943 | 0.9933 | 0.9933 |
| 25℃ | HGRFK | basicity | / | / | 0.9827 | 0.9827 | 0.9475 | 0.9475 |
| 25℃ | HGRFN | basicity | 0.6 | 0.6 | 0.9784 | 0.9784 | 0.9913 | 0.9913 |
| 25℃ | HGRFQ | basicity | 0.6 | 0.6 | 0.7058 | 0.7058 | 0.0098 | 0.0098 |
| 25℃ | HGRFR | basicity | / | / | 0.9951 | 0.9951 | 0.9528 | 0.9528 |
| 25℃ | HGRFS | basicity | 0.8 | 0.8 | 0.8561 | 0.8561 | 0.3088 | 0.3088 |
| 25℃ | HGRFT | basicity | 0.5864 | 0.5864 | 0.729 | 0.729 | 0.02 | 0.02 |
| 25℃ | AGRFG | basicity | 0.8909 | 0.8909 | 0.9236 | 0.9236 | 0.9952 | 0.9952 |
| 25℃ | GGRFG | basicity | 0.784 | 0.784 | 0.439 | 0.439 | 0.9735 | 0.9735 |
| 25℃ | KGRFG | basicity | 0.614 | 0.614 | 0.7439 | 0.7439 | 0.4394 | 0.4394 |
| 25℃ | NGRFG | basicity | 0.691 | 0.691 | 0.3342 | 0.3342 | 0.9984 | 0.9984 |
| 25℃ | PGRFG | basicity | 0.0667 | 0.0667 | 0.0009 | 0.0009 | 0.3507 | 0.3507 |
| 25℃ | QGRFG | basicity | 0.6 | 0.6 | 0.9585 | 0.9585 | 0.9983 | 0.9983 |
| 25℃ | RGRFG | basicity | 0.8909 | 0.8909 | 0.9674 | 0.9674 | 0.9754 | 0.9754 |
| 25℃ | SGRFG | basicity | 0.6 | 0.6 | 0.955 | 0.955 | 0.9984 | 0.9984 |
| 25℃ | TGRFG | basicity | 0.8909 | 0.8909 | 0.9278 | 0.9278 | 0.9952 | 0.9952 |
| 25℃ | VGRFG | basicity | 0.6 | 0.6 | 0.9972 | 0.9972 | 0.9972 | 0.9972 |
| 25℃ | HGRFD | neuter | / | / | 0.9975 | 0.9975 | 0.9832 | 0.9832 |
| 25℃ | HGRFE | neuter | 0.6 | 0.6 | 0.9503 | 0.9503 | 0.9924 | 0.9924 |
| 25℃ | DGRFG | neuter | 0.8 | 0.8 | 0.9788 | 0.9788 | 0.9984 | 0.9984 |
| 25℃ | EGRFG | neuter | 0.6 | 0.6 | 0.9308 | 0.9308 | 0.9984 | 0.9984 |
| 25℃ | NPNPC | neuter | 0.2104 | 0.2104 | 0.824 | 0.824 | 0.8682 | 0.8682 |
| 25℃ | NPNPI | neuter | 0.3556 | 0.3556 | 0.6933 | 0.6933 | 0.9978 | 0.9978 |
| 25℃ | NPNPM | neuter | 0.4969 | 0.4969 | 0.3418 | 0.3418 | 0.976 | 0.976 |
| 25℃ | NPNPP | neuter | 0.8963 | 0.8963 | 0.9927 | 0.9927 | 0.8998 | 0.8998 |
| 25℃ | NPNPV | neuter | 0.0726 | 0.0726 | 0.0704 | 0.0704 | 0.9934 | 0.9934 |
| 25℃ | NPNPY | neuter | 0.5658 | 0.5658 | 0.8458 | 0.8458 | 0.9931 | 0.9931 |
| 25℃ | CPNPT | neuter | 0.9627 | 0.9627 | 0.9954 | 0.9954 | 0.982 | 0.982 |
| 25℃ | IPNPT | neuter | 0.8 | 0.8 | 0.971 | 0.971 | 0.9981 | 0.9981 |
| 25℃ | LPNPT | neuter | 0.9 | 0.9 | 0.9751 | 0.9751 | 0.9994 | 0.9994 |
| 25℃ | MPNPT | neuter | 0.7024 | 0.7024 | 0.2151 | 0.2151 | 0.9982 | 0.9982 |
| 25℃ | QPNPT | neuter | 0.8909 | 0.8909 | 0.982 | 0.982 | 0.9884 | 0.9884 |
| 25℃ | VPNPT | neuter | 0.282 | 0.282 | 0.4137 | 0.4137 | 0.9934 | 0.9934 |
| 25℃ | YPNPT | neuter | 0.8 | 0.8 | 0.9689 | 0.9689 | 0.9988 | 0.9988 |
| 35℃ | DPNPT | acidity | 0.8 | 0.8 | 0.9977 | 0.9977 | 0.9772 | 0.9772 |
| 35℃ | EPNPT | acidity | 0.8 | 0.8 | 0.9984 | 0.9984 | 0.9981 | 0.9981 |
| 35℃ | NPNPD | acidity | 0 | 0 | 0.9883 | 0.9883 | 0.9 | 0.9 |
| 35℃ | NPNPE | acidity | 0.6 | 0.6 | 0.9987 | 0.9987 | 0.9884 | 0.9884 |
| 35℃ | KPNPT | basicity | 0.7657 | 0.7657 | 0.3674 | 0.3674 | 0.9944 | 0.9944 |
| 35℃ | RPNPT | basicity | 0.691 | 0.691 | 0.0978 | 0.0978 | 0.9939 | 0.9939 |
| 35℃ | NPNPK | basicity | 0.2279 | 0.2279 | 0.0004 | 0.0004 | 0.9837 | 0.9837 |
| 35℃ | NPNPR | basicity | 0.9157 | 0.9157 | 0.7232 | 0.7232 | 0.9775 | 0.9775 |
| 35℃ | APNPT | neuter | 0.4102 | 0.4102 | 0.0306 | 0.0306 | 0.9961 | 0.9961 |
| 35℃ | GPNPT | neuter | 0.0448 | 0.0448 | 0.1415 | 0.1415 | 0.9953 | 0.9953 |
| 35℃ | HPNPT | neuter | 0.6983 | 0.6983 | 0.0618 | 0.0618 | 0.9933 | 0.9933 |
| 35℃ | NPNPT | neuter | 0.005 | 0.005 | 0.0952 | 0.0952 | 0.9995 | 0.9995 |
| 35℃ | PPNPT | neuter | 0.9 | 0.9 | 0.985 | 0.985 | 0.9896 | 0.9896 |
| 35℃ | SPNPT | neuter | 0.0308 | 0.0308 | 0.7171 | 0.7171 | 0.9781 | 0.9781 |
| 35℃ | TPNPT | neuter | 0.258 | 0.258 | 0.7343 | 0.7343 | 0.9994 | 0.9994 |
| 35℃ | NPNPA | neuter | 0.0139 | 0.0139 | 0.0627 | 0.0627 | 0.9953 | 0.9953 |
| 35℃ | NPNPG | neuter | 0.2286 | 0.2286 | 0.518 | 0.518 | 0.994 | 0.994 |
| 35℃ | NPNPH | neuter | 0.8393 | 0.8393 | 0.6182 | 0.6182 | 0.994 | 0.994 |
| 35℃ | NPNPN | neuter | 0.2599 | 0.2599 | 0.5607 | 0.5607 | 0.9944 | 0.9944 |
| 35℃ | NPNPQ | neuter | 0.1656 | 0.1656 | 0.428 | 0.428 | 0.994 | 0.994 |
| 35℃ | NPNPS | neuter | 0.0092 | 0.0092 | 0.2801 | 0.2801 | 0.9744 | 0.9744 |
| 35℃ | HGRFA | basicity | 0.8067 | 0.8067 | 0.8542 | 0.8542 | 0.2568 | 0.2568 |
| 35℃ | HGRFG | basicity | 0.784 | 0.784 | 0.4619 | 0.4619 | 0.9881 | 0.9881 |
| 35℃ | HGRFH | basicity | / | / | 0.9862 | 0.9862 | 0.9999 | 0.9999 |
| 35℃ | HGRFK | basicity | / | / | 0.9934 | 0.9934 | 0.998 | 0.998 |
| 35℃ | HGRFN | basicity | / | / | 0.9934 | 0.9934 | 0.9994 | 0.9994 |
| 35℃ | HGRFQ | basicity | 0.5879 | 0.5879 | 0.4937 | 0.4937 | 0.8485 | 0.8485 |
| 35℃ | HGRFR | basicity | 0.6 | 0.6 | 0.9765 | 0.9765 | 0.9927 | 0.9927 |
| 35℃ | HGRFS | basicity | 0.741 | 0.741 | 0.4459 | 0.4459 | 0.9772 | 0.9772 |
| 35℃ | HGRFT | basicity | 0.8909 | 0.8909 | 0.9075 | 0.9075 | 0.9929 | 0.9929 |
| 35℃ | AGRFG | basicity | 0.691 | 0.691 | 0.3975 | 0.3975 | 0.9984 | 0.9984 |
| 35℃ | GGRFG | basicity | 0.741 | 0.741 | 0.5018 | 0.5018 | 0.9907 | 0.9907 |
| 35℃ | KGRFG | basicity | 0.6 | 0.6 | 0.7066 | 0.7066 | 0.3076 | 0.3076 |
| 35℃ | NGRFG | basicity | 0.6 | 0.6 | 0.2352 | 0.2352 | 0.9915 | 0.9915 |
| 35℃ | PGRFG | basicity | 0.6 | 0.6 | 0.9975 | 0.9975 | 0.9995 | 0.9995 |
| 35℃ | QGRFG | basicity | 0.6 | 0.6 | 0.2434 | 0.2434 | 0.9931 | 0.9931 |
| 35℃ | RGRFG | basicity | 0.6983 | 0.6983 | 0.2892 | 0.2892 | 0.9789 | 0.9789 |
| 35℃ | SGRFG | basicity | 0.7024 | 0.7024 | 0.3692 | 0.3692 | 0.8978 | 0.8978 |
| 35℃ | TGRFG | basicity | 0.691 | 0.691 | 0.3841 | 0.3841 | 0.9984 | 0.9984 |
| 35℃ | VGRFG | basicity | / | / | 0.996 | 0.996 | 0.9993 | 0.9993 |
| 35℃ | HGRFD | neuter | 0.6 | 0.6 | 0.9588 | 0.9588 | 0.9951 | 0.9951 |
| 35℃ | HGRFE | neuter | 0.6 | 0.6 | 0.9565 | 0.9565 | 0.9765 | 0.9765 |
| 35℃ | DGRFG | neuter | 0.6983 | 0.6983 | 0.4101 | 0.4101 | 0.9924 | 0.9924 |
| 35℃ | EGRFG | neuter | 0.6 | 0.6 | 0.2861 | 0.2861 | 0.9924 | 0.9924 |
| 35℃ | NPNPC | neuter | 0.784 | 0.784 | 0.9154 | 0.9154 | 0.8998 | 0.8998 |
| 35℃ | NPNPI | neuter | 0.1724 | 0.1724 | 0.1294 | 0.1294 | 0.9999 | 0.9999 |
| 35℃ | NPNPM | neuter | 0 | 0 | 0.0162 | 0.0162 | 0.9934 | 0.9934 |
| 35℃ | NPNPP | neuter | 0.98 | 0.98 | 0.9855 | 0.9855 | 0.8501 | 0.8501 |
| 35℃ | NPNPV | neuter | 0.0201 | 0.0201 | 0.0269 | 0.0269 | 0.9653 | 0.9653 |
| 35℃ | NPNPY | neuter | 0.288 | 0.288 | 0.7697 | 0.7697 | 0.9865 | 0.9865 |
| 35℃ | CPNPT | neuter | 0.786 | 0.786 | 0.8412 | 0.8412 | 0.982 | 0.982 |
| 35℃ | IPNPT | neuter | 0.4545 | 0.4545 | 0.8804 | 0.8804 | 0.9983 | 0.9983 |
| 35℃ | LPNPT | neuter | 0.6 | 0.6 | 0.9205 | 0.9205 | 0.9937 | 0.9937 |
| 35℃ | MPNPT | neuter | 0.6 | 0.6 | 0.1472 | 0.1472 | 0.9931 | 0.9931 |
| 35℃ | QPNPT | neuter | 0.4027 | 0.4027 | 0.9918 | 0.9918 | 0.6 | 0.6 |
| 35℃ | VPNPT | neuter | 0.0054 | 0.0054 | 0.1497 | 0.1497 | 0.9934 | 0.9934 |
| 35℃ | YPNPT | neuter | 0.7024 | 0.7024 | 0.2222 | 0.2222 | 0.9969 | 0.9969 |
| 45℃ | DPNPT | acidity | 0.98 | 0.98 | 0.9976 | 0.9976 | 0.9944 | 0.9944 |
| 45℃ | EPNPT | acidity | 0.8909 | 0.8909 | 0.9925 | 0.9925 | 0.9944 | 0.9944 |
| 45℃ | NPNPD | acidity | 0.6 | 0.6 | 0.9827 | 0.9827 | 0.8909 | 0.8909 |
| 45℃ | NPNPE | acidity | 0.8526 | 0.8526 | 0.991 | 0.991 | 0.8914 | 0.8914 |
| 45℃ | KPNPT | basicity | 0.3913 | 0.3913 | 0.0178 | 0.0178 | 0.9898 | 0.9898 |
| 45℃ | RPNPT | basicity | 0.6 | 0.6 | 0.0195 | 0.0195 | 0.966 | 0.966 |
| 45℃ | NPNPK | basicity | 0.258 | 0.258 | 0.0117 | 0.0117 | 0.9957 | 0.9957 |
| 45℃ | NPNPR | basicity | 0.0748 | 0.0748 | 0.2218 | 0.2218 | 0.9898 | 0.9898 |
| 45℃ | APNPT | neuter | 0.7024 | 0.7024 | 0.0951 | 0.0951 | 0.9996 | 0.9996 |
| 45℃ | GPNPT | neuter | 0.1451 | 0.1451 | 0.2356 | 0.2356 | 0.9862 | 0.9862 |
| 45℃ | HPNPT | neuter | 0.7171 | 0.7171 | 0.0108 | 0.0108 | 0.9997 | 0.9997 |
| 45℃ | NPNPT | neuter | 0.0347 | 0.0347 | 0.2118 | 0.2118 | 0.9775 | 0.9775 |
| 45℃ | PPNPT | neuter | 0.8 | 0.8 | 0.9404 | 0.9404 | 0.9907 | 0.9907 |
| 45℃ | SPNPT | neuter | 0.0038 | 0.0038 | 0.0245 | 0.0245 | 0.9946 | 0.9946 |
| 45℃ | TPNPT | neuter | 0.1181 | 0.1181 | 0.7142 | 0.7142 | 0.9918 | 0.9918 |
| 45℃ | NPNPA | neuter | 0.1072 | 0.1072 | 0.1787 | 0.1787 | 0.9862 | 0.9862 |
| 45℃ | NPNPG | neuter | 0.1714 | 0.1714 | 0.4184 | 0.4184 | 0.9951 | 0.9951 |
| 45℃ | NPNPH | neuter | 0.8627 | 0.8627 | 0.6518 | 0.6518 | 0.9744 | 0.9744 |
| 45℃ | NPNPN | neuter | 0.455 | 0.455 | 0.6478 | 0.6478 | 0.9957 | 0.9957 |
| 45℃ | NPNPQ | neuter | 0.4962 | 0.4962 | 0.0497 | 0.0497 | 0.9943 | 0.9943 |
| 45℃ | NPNPS | neuter | 0.0007 | 0.0007 | 0.2508 | 0.2508 | 0.9 | 0.9 |
| 45℃ | HGRFA | basicity | 0.6 | 0.6 | 0.9755 | 0.9755 | 0.9967 | 0.9967 |
| 45℃ | HGRFG | basicity | 0.0608 | 0.0608 | 0.0053 | 0.0053 | 0.4655 | 0.4655 |
| 45℃ | HGRFH | basicity | 0.6 | 0.6 | 0.9931 | 0.9931 | 0.9947 | 0.9947 |
| 45℃ | HGRFK | basicity | 0.6 | 0.6 | 0.9895 | 0.9895 | 0.9901 | 0.9901 |
| 45℃ | HGRFN | basicity | 0.6 | 0.6 | 0.9943 | 0.9943 | 0.9953 | 0.9953 |
| 45℃ | HGRFQ | basicity | 0.8909 | 0.8909 | 0.9918 | 0.9918 | 0.9984 | 0.9984 |
| 45℃ | HGRFR | basicity | 0.2632 | 0.2632 | 0.9744 | 0.9744 | 0.3072 | 0.3072 |
| 45℃ | HGRFS | basicity | 0.2 | 0.2 | 0.9944 | 0.9944 | 0.9967 | 0.9967 |
| 45℃ | HGRFT | basicity | 0.8 | 0.8 | 0.9895 | 0.9895 | 0.9981 | 0.9981 |
| 45℃ | AGRFG | basicity | 0.8 | 0.8 | 0.8493 | 0.8493 | 0.4044 | 0.4044 |
| 45℃ | GGRFG | basicity | 0.4765 | 0.4765 | 0.0932 | 0.0932 | 0.9854 | 0.9854 |
| 45℃ | KGRFG | basicity | / | / | 0.9951 | 0.9951 | 0.9944 | 0.9944 |
| 45℃ | NGRFG | basicity | 0.731 | 0.731 | 0.4474 | 0.4474 | 0.9782 | 0.9782 |
| 45℃ | PGRFG | basicity | / | / | 0.9983 | 0.9983 | 0.9974 | 0.9974 |
| 45℃ | QGRFG | basicity | 0.6 | 0.6 | 0.2472 | 0.2472 | 0.9838 | 0.9838 |
| 45℃ | RGRFG | basicity | 0.8 | 0.8 | 0.5214 | 0.5214 | 0.9854 | 0.9854 |
| 45℃ | SGRFG | basicity | 0.731 | 0.731 | 0.4563 | 0.4563 | 0.9782 | 0.9782 |
| 45℃ | TGRFG | basicity | 0.8 | 0.8 | 0.55 | 0.55 | 0.9907 | 0.9907 |
| 45℃ | VGRFG | basicity | 0 | 0 | 0.9965 | 0.9965 | 0.9958 | 0.9958 |
| 45℃ | HGRFD | neuter | 0.4545 | 0.4545 | 0.9142 | 0.9142 | 0.9931 | 0.9931 |
| 45℃ | HGRFE | neuter | 0.4545 | 0.4545 | 0.9181 | 0.9181 | 0.9931 | 0.9931 |
| 45℃ | DGRFG | neuter | 0.0237 | 0.0237 | 0.02 | 0.02 | 0.9894 | 0.9894 |
| 45℃ | EGRFG | neuter | 0.6 | 0.6 | 0.4203 | 0.4203 | 0.9765 | 0.9765 |
| 45℃ | NPNPC | neuter | 0.741 | 0.741 | 0.9134 | 0.9134 | 0.8 | 0.8 |
| 45℃ | NPNPI | neuter | 0 | 0 | 0.1792 | 0.1792 | 0.9895 | 0.9895 |
| 45℃ | NPNPM | neuter | 0.0014 | 0.0014 | 0.0242 | 0.0242 | 0.9944 | 0.9944 |
| 45℃ | NPNPP | neuter | 0.569 | 0.569 | 0.272 | 0.272 | 0.8 | 0.8 |
| 45℃ | NPNPV | neuter | 0.0034 | 0.0034 | 0.1026 | 0.1026 | 0.9918 | 0.9918 |
| 45℃ | NPNPY | neuter | 0.0896 | 0.0896 | 0.0705 | 0.0705 | 0.9944 | 0.9944 |
| 45℃ | CPNPT | neuter | 0.5299 | 0.5299 | 0.3251 | 0.3251 | 0.8771 | 0.8771 |
| 45℃ | IPNPT | neuter | 0.6877 | 0.6877 | 0.3456 | 0.3456 | 0.9857 | 0.9857 |
| 45℃ | LPNPT | neuter | 0.0143 | 0.0143 | 0.4331 | 0.4331 | 0.9981 | 0.9981 |
| 45℃ | MPNPT | neuter | 0.731 | 0.731 | 0.4001 | 0.4001 | 0.9978 | 0.9978 |
| 45℃ | QPNPT | neuter | 0.9184 | 0.9184 | 0.9931 | 0.9931 | 0.8998 | 0.8998 |
| 45℃ | VPNPT | neuter | 0.0604 | 0.0604 | 0.2616 | 0.2616 | 0.9944 | 0.9944 |
| 45℃ | YPNPT | neuter | 0.6368 | 0.6368 | 0.1891 | 0.1891 | 0.9943 | 0.9943 |

**Supplementary Table 5** Correlation Coefficients (R^2^) under different chromatographic conditions in the six-parameter model of T and pH.

| **pH** | **R^2^** | **T** | **R^2^** | **φ** | **R^2^** |
| --- | --- | --- | --- | --- | --- |
| 2 | 0.7004 | 25℃ | 0.5655 | 8% | 0.7547 |
| 3 | 0.0938 | 35℃ | 0.4796 | 10% | 0.4913 |
| 4 | 0.0796 | 45℃ | 0.3618 | 12% | 0.3018 |
| 5 | 0.1304 |  |  | 14% | 0.1209 |
| 6 | 0.1656 |  |  | 20% | 0.4893 |
| 7 | 0.5001 |  |  | 22% | 0.4076 |
| 8 | 0.3465 |  |  | 24% | 0.2282 |
|  |  |  |  | 26% | 0.2703 |

**Supplementary Table 6** Correlation Coefficients (R^2^) under different chromatographic conditions in the six-parameter model of pH and φ or $P_{m}^{N}$.

| **pH** | **R^2^** | **T** | **R^2^** | **φ** | **R^2^** |
| --- | --- | --- | --- | --- | --- |
| 2 | 0.785 | 25℃ | 0.7166 | 8% | 0.9296 |
| 3 | 0.2159 | 35℃ | 0.7515 | 10% | 0.9194 |
| 4 | 0.2397 | 45℃ | 0.7881 | 12% | 0.8976 |
| 5 | 0.4147 |  |  | 14% | 0.8685 |
| 6 | 0.6512 |  |  | 20% | 0.679 |
| 7 | 0.8186 |  |  | 22% | 0.5969 |
| 8 | 0.7984 |  |  | 24% | 0.5793 |
|  |  |  |  | 26% | 0.5785 |

| **pH** | **R^2^** | **T** | **R^2^** | $\boldsymbol{P}_{\boldsymbol{m}}^{\boldsymbol{N}}$ | **R^2^** |
| --- | --- | --- | --- | --- | --- |
| 2 | 0.7868 | 25℃ | 0.7093 | 0.90 | 0.9303 |
| 3 | 0.2093 | 35℃ | 0.734 | 0.87 | 0.9188 |
| 4 | 0.2271 | 45℃ | 0.7877 | 0.85 | 0.896 |
| 5 | 0.41 |  |  | 0.83 | 0.8649 |
| 6 | 0.651 |  |  | 0.76 | 0.6886 |
| 7 | 0.82 |  |  | 0.73 | 0.5964 |
| 8 | 0.7957 |  |  | 0.71 | 0.574 |
|  |  |  |  | 0.69 | 0.5819 |

**Supplementary Table 7** The root mean square error (RMSE) of the six-parameter model in the 10-fold cross-validation.

| **Models** | $k=\frac{{10}^{a+\frac{b}{T}}+{10}^{c+\frac{d}{T}}{10}^{(pH-e-\frac{f}{T})}}{1+{10}^{(pH-e-\frac{f}{T})}}$ | | $k=\frac{{10}^{A+B\varphi}+{10}^{C+D\varphi}{10}^{(pH-E-F\varphi)}}{1+{10}^{(pH-E-F\varphi)}}$ | | | $k=\frac{{10}^{A+BP_{m}^{N}}+{10}^{C+DP_{m}^{N}}{10}^{(pH-E-FP_{m}^{N})}}{1+{10}^{(pH-E-FP_{m}^{N})}}$ | | |
| --- | --- | --- | --- | --- | --- | --- | --- | --- |
| **Group** | All pentapeptides | Acid pentapeptides | All pentapeptides | Acid pentapeptides | Neutral pentapeptides | All pentapeptides | Acid pentapeptides | Neutral pentapeptides |
| **RMSE** | 0.48 | 0.18 | 0.78 | 0.24 | 0.29 | 0.78 | 0.25 | 0.32 |
|  | 0.46 | 0.21 | 0.79 | 0.23 | 0.36 | 0.75 | 0.23 | 0.34 |
|  | 0.48 | 0.20 | 0.80 | 0.29 | 0.29 | 0.68 | 0.20 | 0.33 |
|  | 0.45 | 0.22 | 0.71 | 0.20 | 0.30 | 0.93 | 0.27 | 0.38 |
|  | 0.40 | 0.18 | 0.76 | 0.30 | 0.40 | 0.70 | 0.29 | 0.37 |
|  | 0.49 | 0.19 | 0.77 | 0.38 | 0.34 | 0.86 | 0.25 | 0.33 |
|  | 0.50 | 0.19 | 0.80 | 0.31 | 0.45 | 0.75 | 0.35 | 0.36 |
|  | 0.52 | 0.21 | 0.85 | 0.23 | 0.41 | 0.84 | 0.25 | 0.34 |
|  | 0.49 | 0.24 | 0.65 | 0.26 | 0.31 | 0.73 | 0.26 | 0.40 |
|  | 0.49 | 0.16 | 0.83 | 0.25 | 0.36 | 0.73 | 0.31 | 0.36 |
| **Ave** | 0.48 | 0.20 | 0.77 | 0.27 | 0.35 | 0.78 | 0.27 | 0.35 |


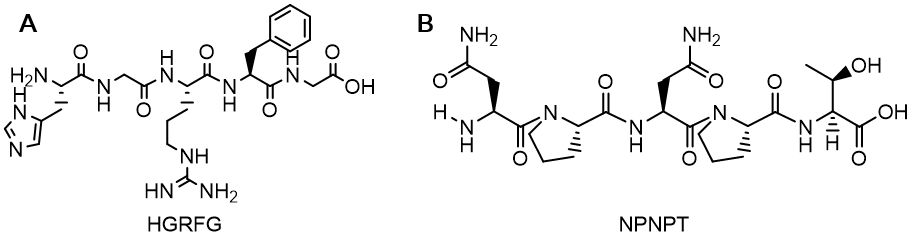


**Supplementary Figure 1** The chemical structure diagram of pentapeptides HGRFG (A), NPNPT (B).


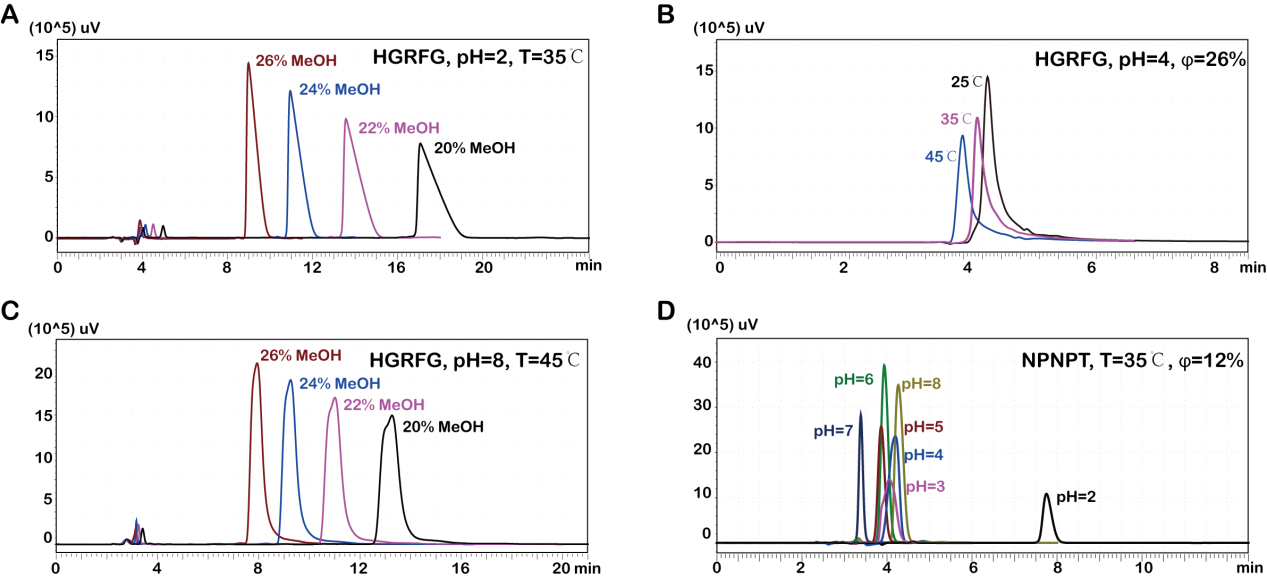


**Supplementary Figure 2** The comparative chromatograms of HGRFG at pH = 2 and T = 35℃ (A), pH = 4 and φ = 26% (B), pH = 8 and T = 45℃ (C), and NPNPT at T = 35℃ and φ = 12% (D).


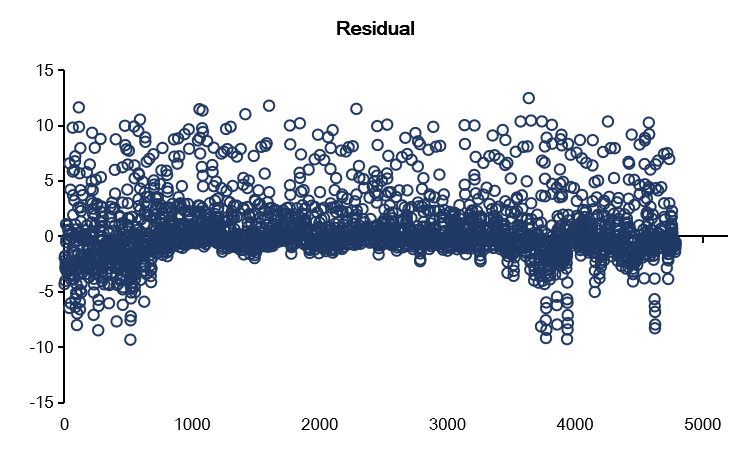


**Supplementary Figure 3** The residuals of predicted k-value and experimental k-value were symmetrical distribution around the y = 0 axis in the six-parameter model of pH and T.


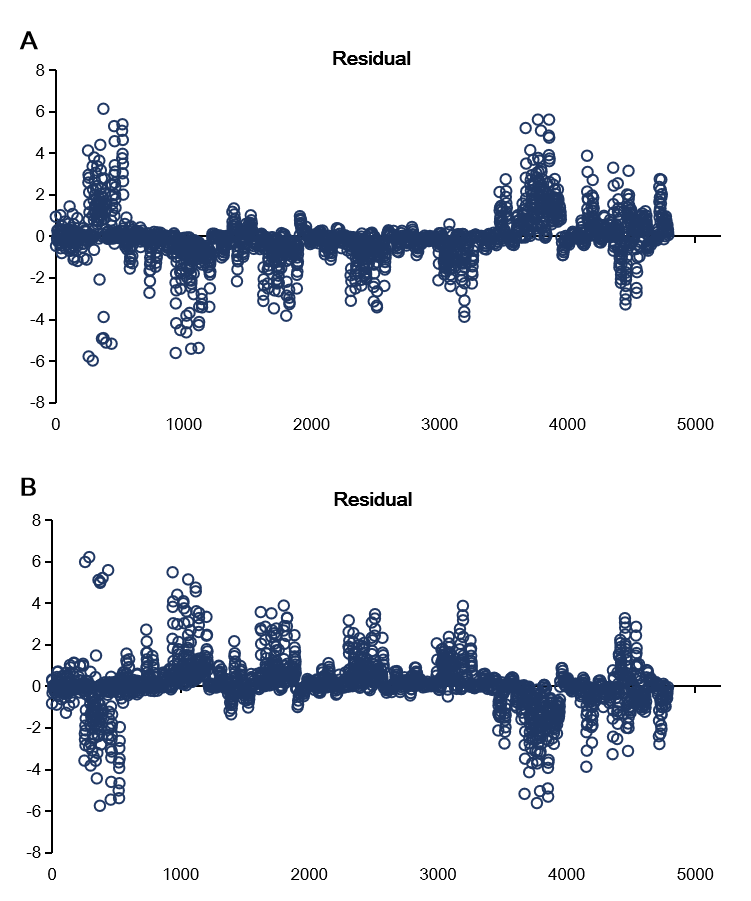


**Supplementary Figure 4** The residuals of predicted k-value and experimental k-value were symmetrical distribution around the y = 0 axis in the six-parameter model of pH and φ (A) or pH and $P_{m}^{N}$ (B).

**
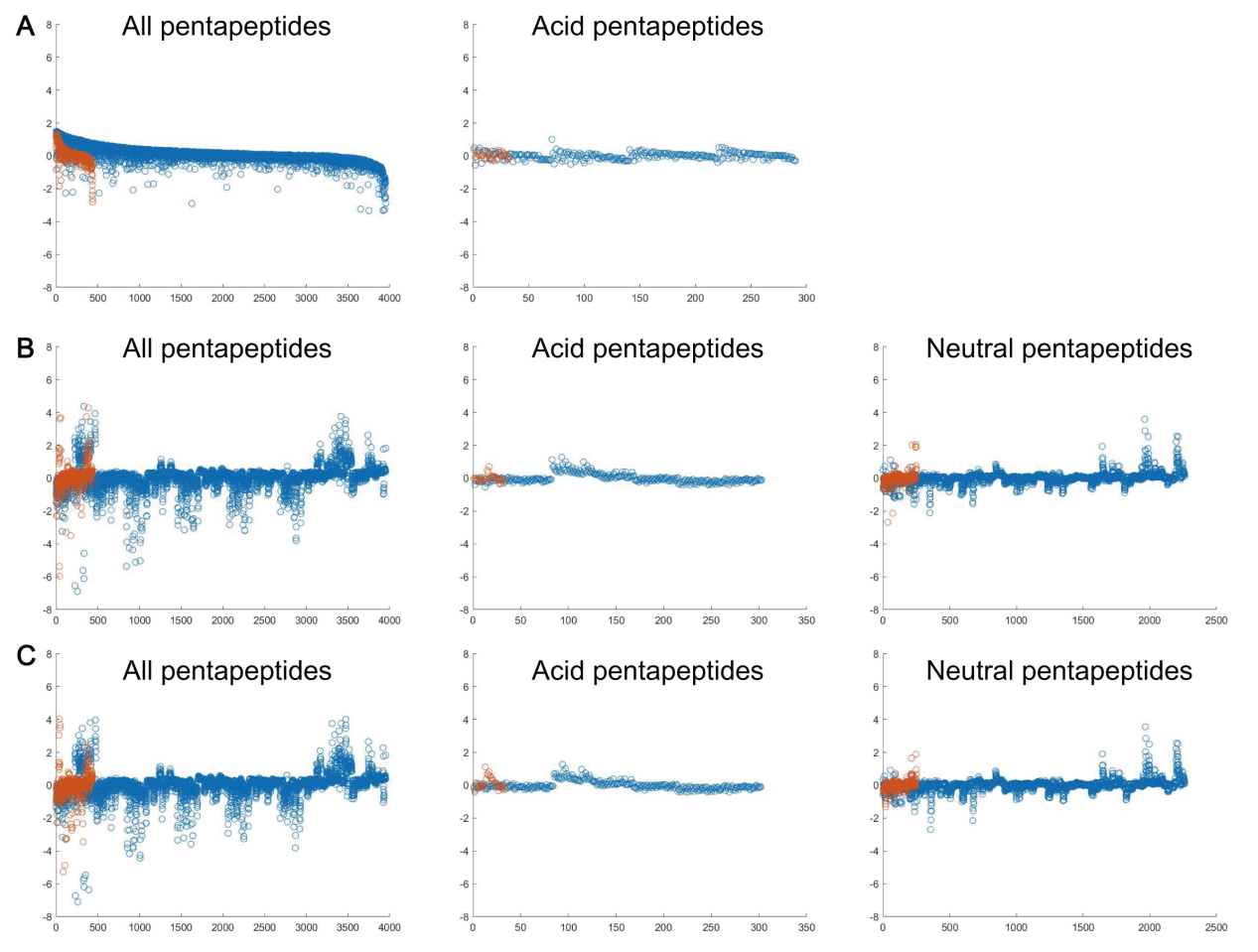
**

**Supplementary Figure 5** The residuals of training and testing sets in the 10-fold cross-validation in the six-parameter model of pH and T for all pentapeptides (left panel) and acid pentapeptides (right panel) (A), the six-parameter model of pH and φ for all pentapeptides (left panel), acid pentapeptides (middle panel) and neutral pentapeptides (right panel) (B), the six-parameter model of pH and $P_{m}^{N}$ for all pentapeptides (left panel), acid pentapeptides (middle panel) and neutral pentapeptides (right panel) (C). ( training sets, testing sets)
